# Supplementary material for: Safe handovers for every patient: an interrupted time series analysis to test the effect of a structured discharge bundle in Dutch hospitals
Source: BMJ Open. 2019 Jun 4;9(6):e023446. doi: 10.1136/bmjopen-2018-023446 (PMC6561436; doi:10.1136/bmjopen-2018-023446)
Supplement: Supplementary file 1 [file bmjopen-2018-023446supp001.pdf]

Supplement Table 1. Adherence to the Intervention Protocol

	Hospital 1	Hospital 2	Hospital 3	Hospital 4	Hospital 5	Hospital 6	Hospital 7	Hospital 8
Pre-intervention<br>Implementation<br>Post-intervention	Sep ’15 - Feb ’16<br>March ’16 - April ‘16<br>May ’16 – Oct ’16	Oct ’15 - March ’16<br>April ’16 - May ’16<br>June ’16 - Nov ’16	Jan ’16 - June ’16<br>July ’16 - Aug ’16<br>Sep ’16 – Feb ’17	Dec ’15 - May ’16<br>June ’16 - July ’16<br>Aug ’16 - Jan ’16	March ’16 – Aug ’16<br>Sep ’16 - Oct ’16<br>Nov ’16 – April ’17	April ’16 – Sep ’16<br>Oct ’16 - Nov ’16<br>Dec ’16 – May ’17	May ’16 – Oct ’16<br>Nov ’16 - Dec ’16<br>Jan ’17 – June ’17	April ’16 – Sep ’16<br>Oct ’16 - Nov ’16<br>Dec ’16 – May ’17
**Leadership and education of project leaders**								
Who were present at the kick off meeting February, 2016?	Hospital president; local project leader; 2 team leaders (nurses); 2 physicians; nurse; pharmacists	Local project leader; geriatrician; head of the liaison department; physician	2 local project leaders;<br>head of the liaison department	Chief of staff; local project leader; team leader surgery ward (nurse); head of the liaison department	Local project leader; head of the liaison department; manager patient logistics; 2 team leaders (nurses)	2 local project leaders	Local project leader	Local project leader; senior researcher transitional care; medical specialist,
Who were present at the first feedback session?	Project leader; head of the liaison department	Project leader; pharmacist; communication assistant	2 local project leaders; liaison nurse	Project leader; liaison nurse; nurse geriatrics	Local project leader	2 local project leaders	-	2 project leaders
Who were present at the second feedback session?	Project leader	Project leader	2 project leaders	-	Project leader	-	Project leader	2 project leaders
Implementation points	*10*	*8*	*8*	*7*	*7*	*4*	*2*	*7*
**Project group**								
Was there a local TIP project group, and who participated?	Yes, project leader; 2 senior nurses of participating wards, management assistant	Yes, project leader; geriatrician; head liaison department, physician; pharmacist; communication assistant; manager Security & Services	Yes, 2 project leaders; 2 residents; 2 medical specialists; nurse; liaison nurse; pharmacist; manager	Yes, chief of staff; project leader; 2 team leaders (nurses); head liaison department; orthopedist	Yes, project leader; head liaison department; 2 medical specialists, geriatrician	No	No	Yes, local project leader; 3 medical specialists; 2 residents, manager quality and safety; manager process optimization; medical director
How often did the local project group meet?<br>1 point per meeting	Monthly for 2 months, during pilot period every week (2 months).	Every five weeks during pre-intervention and pilot period	2 times, before pilot period.	Every two weeks during pre-intervention period	Every six weeks, during pre-intervention and pilot period	-	-	Monthly during pre-intervention, pilot period and first two months of post-

								intervention period
Implementation points	*10*	*12*	*10*	*18*	*10*	*0*	*0*	*17*
**Implementation of TIP elements**								
Was it policy to set a discharge date within 48h after admission?	Yes	Yes	Yes	Yes	Yes	Yes	Yes	Yes
Was the planned discharge date communicated to patients?	Yes	Yes	Yes	Yes	Yes	No	Yes	Yes
Was it policy to start with arrangement of required post-discharge care within 48h after admission?	No, liaison nurse has to wait for final discharge date	No, liaison nurse has to wait for final discharge date	Yes	Yes	No, liaison department is overloaded	Yes	No, liaison nurse has to wait for final discharge date	Yes
Was it policy to set up patient handovers within 48h after admission?	No	No	No	No	No	No	No	No
Did physicians hold discharge conversations, using a checklist during the pilot period?	No	No	No	No	No	No	No	No
Does the nurse holds a discharge conversation, using a checklist?	Yes	No	Yes	Yes	Yes	No	Yes	Yes
Was the patient discharge letter implemented?	Yes, but only for some diagnosis at internal medicine ward.	No	No	No	No	Yes , but only at internal medicine ward for frail older patients.	No	No, a discharge summary was implemented instead.
Implementation points	*3.5*	*2*	*4*	*4*	*3*	*2.5*	*3*	*4.5*
**Education of physicians and nurses**								

How were physicians and nurses informed about the TIP and how often?		Kick-off meeting at participating wards; during morning report; working instructions were sent by email to all physicians		Kick-off meeting; meeting at participating wards; E-learning; 1 feedback meeting		During morning reports; project leader informed every physician separately; intranet; email		During morning reports; intranet; email; posters & pocket cards; and project leaders went to participating wards to inform physicians and nurses		Email and project leader went to participating wards		Kick-off meeting, during several morning reports		Physicians were not educated with regard to the intervention		During several morning report; email; project leaders went to participating wards to inform physicians and nurses; medical specialists from project group informed physicians in person	
Did physicians and/or nurses receive feedback with regard to their discharge letters and if yes, how often?		No		No		No		No		No		Only for nurses		No		Yes, daily on internal medicine and monthly on surgery ward, via email.	
Implementation points		*3*		*4*		*4*		*5*		*2*		*2.5*		*0*		*5*	
**Total implementation points**		**26.5**		**26**		**26**		**34**		**22**		**9**		**5**		**33.5**	
**Pre-intervention vs. post-intervention period scores**																	
**Pre-intervention period**																	
median	% letters within 24h	8.15	9.0	0.90	47.3	6.71	23.5	10.48	13.1	0.79	50.0	6.79	9.2	14.21	7.6	5.83	20.9
**Post-intervention**																	
median	% letters within 24h	9.08	19.5	1.0	48.5	5.48	24.2	5.79	19.7	0.29	49.6	7.98	16.7	22.44	1.5	0.83	53.8
