# Supplementary material for: Safe handovers for every patient: an interrupted time series analysis to test the effect of a structured discharge bundle in Dutch hospitals
Source: BMJ Open. 2019 Jun 4;9(6):e023446. doi: 10.1136/bmjopen-2018-023446 (PMC6561436; doi:10.1136/bmjopen-2018-023446)
Supplement: Author's manuscript [file bmjopen-2018-023446.draft_revisions.pdf]

BMJ Open is committed to open peer review. As part of this commitment we make the peer review history of every article we publish publicly available.

When an article is published we post the peer reviewers' comments and the authors' responses online. We also post the versions of the paper that were used during peer review. These are the versions that the peer review comments apply to.

The versions of the paper that follow are the versions that were submitted during the peer review process. They are not the versions of record or the final published versions. They should not be cited or distributed as the published version of this manuscript.

BMJ Open is an open access journal and the full, final, typeset and author-corrected version of record of the manuscript is available on our site with no access controls, subscription charges or pay-per-view fees (http://bmjopen.bmj.com).

If you have any questions on BMJ Open's open peer review process please email info.bmjopen@bmj.com

BMJ Open

A safe handover for every patient: an interrupted time series analysis to test the effect of a structured discharge bundle

Journal:	*BMJ Open*
Manuscript ID	bmjopen-2018-023446
Article Type:	Research
Date Submitted by the Author:	09-Apr-2018

Complete List of Authors:	van Seben, Rosanne; AMC, Internal Medicine, section of Geriatric Medicine<br>Geerlings, Suzanne; AMC, Internal Medicine, Division of Infectious Diseases<br>Maaskant, Jolanda; AMC, Emma Children's Hospital<br>Buurman, Bianca; Academic Medical Center, Internal Medicine, section of Geriatric Medicine

Keywords:	Discharge Bundle, Patient Handovers, Discharge Letter, Patient Safety, Interrupted Time Series, Quality Improvement

SCHOLARONE™
Manuscripts

A safe handover for every patient: an interrupted time series analysis to test the effect of a structured discharge bundle

Rosanne van Seben (corresponding author)
Department of Internal Medicine, Division of Geriatric Medicine, Academic Medical Center
PO Box 22660, 1100 DD Amsterdam, The Netherlands
r.vanseben@amc.uva.nl, +312 56 61647

Suzanne E. Geerlings
Department of Internal Medicine, Division of Infectious Diseases, Academic Medical Center, Amsterdam, The Netherlands

Jolanda M. Maaskant
Emma Children's Hospital, Academic Medical Center Amsterdam, Amsterdam, The Netherlands.

Bianca M. Buurman
Department of Internal Medicine, Division of Geriatric Medicine, Academic Medical Center, Amsterdam, The Netherlands & ACHIEVE Centre of Expertise, Faculty of Health, Amsterdam University of Applied Sciences, Amsterdam, the Netherlands

On behalf of the TIP study group: Hanneke Pullens, Barbara van Munster, Bianka Mennema, Alie Haze-Visser, Agnes van ‘t Hof, Mariët Dirkzwager, Stella de Regt, Lisette Bruns, Suzan Vroomen, Jolanda Maaskant, Rosanne van Seben, Suzanne Geerlings and Bianca Buurman

Key words: Discharge Bundle, Patient Handovers, Patient Safety, Interrupted Time Series, Quality Improvement

Word count: 3214

For peer review only

For peer review only - http://bmjopen.bmj.com/site/about/guidelines.xhtml

Abstract

Objective Patient handovers are often delayed, patients are hardly involved in their discharge process and hospital-wide standardized discharge procedures are lacking. The aim of this study was to implement a structured discharge bundle and to test the effect on timeliness of medical and nursing handovers.

Design Interrupted time series with six pre-intervention and six post-intervention data collection points (September 2015 through June 2017).

Setting Internal medicine and surgical wards.

Participants Patients (≥18 years) admitted for more than 48h to surgical or internal medicine wards.

Intervention The Transfer Intervention Procedure (TIP), containing four elements: within 48h after admission planning discharge date, arrangement of post-discharge care, preparing handovers and personalized patient discharge letter; and a discharge conversation 12-24h before discharge.

Outcome measures The number of medical and nursing handovers sent within 24h. Secondary outcomes were the median time between discharge and medical handovers, length of hospital stay (LOS) and unplanned readmissions.

Results Pre-intervention 1039 and post-intervention 1052 patient records were reviewed. No significant change in levels and slopes was observed in the number of medical and nursing handovers sent within 24h. The median (interquartile range) time between discharge and medical handovers decreased from 6.15 (0.96-15.96) to 4.08 (0.33-13.67), but no significant difference in levels and slopes was found. No intervention-effect was observed for LOS and readmission. In subgroup analyses, a reduction of 5.6 days in the median time between discharge and medical handovers was observed in hospitals with high protocol adherence and much attention for implementation.

Conclusion Implementation of a structured discharge bundle did not lead to improved timeliness of patient handovers. However, large inter-hospital variation was observed and an intervention effect on the median time between discharge and medical handovers was seen in hospitals with high

For peer review only - http://bmjopen.bmj.com/site/about/guidelines.xhtml

protocol adherence. Future interventions should continue to create awareness of the importance of timely handovers.

Trial Registration: Dutch Trial Registry: NTR5951

Strengths and limitations of this study

- This study aimed to implement a structured discharge bundle to improve patient handovers for every patient.
- The study design, i.e. Interrupted Time Series Analysis, provided valuable information on pre-intervention trends, which strengthens the results.
- Sensitivity analysis provided important insight into the inter-hospital variation and differences in intervention effects among hospitals.
- Although it would have been informative, data on the content of medical handovers were not collected, as not on accurateness and timeliness of medication handovers.
- It was not possible to evaluate percentages of compliance with the study protocol and, although in line with the observed efforts, the process evaluation with the project leaders might have been an overestimation.

For peer review only

For peer review only - http://bmjopen.bmj.com/site/about/guidelines.xhtml

Introduction

As hospital stays have become shorter and full recovery often takes place at home,[1] a safe transition from hospital to home or nursing home has become more and more important. Besides, a rising number of older chronically ill patients who move along the care continuum, requires continuity of care[2 3]. However, transitions from hospital to primary care settings are still considered a high-risk process. Patients are discharged with little coordination or follow-up and are hardly involved in their own discharge process.[4 5]

Inadequate transitions may have serious implications for patient safety and quality of care. Post-discharge adverse events such as medication errors, can be the consequence of insufficient or lacking communication between hospital and primary care providers, thereby contributing to higher resource use and unplanned readmission rates[6-11]. In fact, unplanned readmission rates in the first month post-discharge are as high as 20%[12] and a recent study shows that half of them are deemed preventable.[11]

The root of a safe transition from hospital to home or nursing homes is a timely transfer of accurate medical discharge information[8 13]. The general practitioner (GP) can only take over responsibility for a patient safely, when receiving a medical handover containing accurate information on, e.g., medications, and follow-up.[13] Nonetheless, a review of Kripalani et al. showed that discharge letters are often not available, lack important information or are not sent in a timely manner[8]. Also, a more recent study performed in 20 Dutch hospitals showed that in 10% of cases discharge letters were missing and the remainder was on average sent after one week,[14] even though unplanned readmissions most frequently occur within the first week post-discharge[15].

Previous studies that aimed to improve patient handovers, mainly focused on specific high risk populations and targeted patient-related factors[16-18]. Although such interventions on individualized discharge planning or transitional care have been effective in reducing readmission[16 17] and post-discharge mortality rates,[18-20] organizational factors that form the basis of a safe handover

For peer review only - http://bmjopen.bmj.com/site/about/guidelines.xhtml

should also be optimally arranged[13 21]. In fact, in order to ensure patient safety and continuity of care, early discharge planning, a structured discharge process and timely handovers might be essential[13 21 22]. Besides, given that patients are often unprepared at time of discharge and uncertainties about aspects such as treatment or medication may exist,[5] patient education, e.g., in terms of a proper discharge conversation, should also be an important aspect of the discharge process[6 7].

The aim of this study was, therefore, to implement a structured discharge process, the Transfer Intervention Procedure (TIP), in eight hospitals. The TIP contains four elements: within 48h after admission planning the discharge date, arrangement of required post-discharge care, and preparing patient handovers and a personalized patient discharge letter; and holding a discharge conversation 12 tot 24h before discharge. We tested whether the TIP improved timeliness of medical and nursing handovers and investigated the effect of the TIP procedure on length of hospital stay and unplanned readmissions within 30-days post-discharge.

Methods

Study design and setting

We evaluated the implementation of the TIP discharge bundle in an interrupted time series (ITS), which is the strongest design when a randomized controlled trial is not feasible[23 24]. The trial protocol[25] was based on the recommendations for ITS studies,[23] and we adhered the SQUIRE guidelines for quality improvement reporting.[26] Outcomes before and after implementation of the TIP bundle were compared, by conducting six pre-intervention and six post-intervention measurements. During the implementation period (two months) no measurements were conducted. Throughout the Netherlands, one university and seven regional teaching hospitals participated and the TIP was implemented at one of their surgical and one of their internal medicine wards. February 2016, a kick off meeting was held. Between March 2016 and November 2016, hospitals started with implementation. During this period, regular meetings were held to provide feedback, discuss

For peer review only - http://bmjopen.bmj.com/site/about/guidelines.xhtml

implementation, and share experiences. Data collection started September 2015 and ended June 2017 (Supplement Table 1). All patients (aged ≥18 years) admitted for more than 48h were eligible for inclusion. The Medical Ethics Research Committee confirmed that the Medical Research Involving Human Subjects Act did not apply to this research project and official approval was not required.

Discharge procedures in the Netherlands

In the Netherlands, primary care standards are relatively high and basically every person has a general practitioner (GP). When a person is hospitalized, responsibility is taken over from the GP by the medical specialist. After discharge, patient care becomes responsibility of the GP again. It is policy for hospitals to provide patient handovers to the GP. However, there are no clear guidelines for hospitals how to arrange their discharge process. The Dutch healthcare inspectorate,[27] indicated that standardized discharge procedures are lacking and errors that occur during handovers are often resolved informally.

The current study was part of a large national program, initiated by the Dutch Ministry of Health, Welfare, and Sport (abbreviated in Dutch: VWS): 'Addressing Waste in Health Care'. This program was set up in order to reduce inefficiencies in the provision of health care. As part of this program, a TIP study group was established, comprising a study coordinator, two supervisors, one clinical epidemiologist, a policy officer from the Ministry of VWS and local project leaders from the eight participating Dutch hospitals that implemented the TIP bundle. Regular meetings were held to report results and provide feedback, to discuss implementation, share experience and learn from each other's practices.

Intervention

Figure 1 illustrates how the TIP bundle forms the basis of a safe handover from hospital to primary care for every patient, and if applicable, for patients discharged with post-discharge care or for

For peer review only - http://bmjopen.bmj.com/site/about/guidelines.xhtml

complex patients who require a case manager or transitional care. As previously described,[25 28] the TIP bundle was developed using input from focus group meetings with professionals, patient surveys and literature. The TIP discharge bundle consists of four elements: 1) planning the discharge date within 48 hours after admission and communication of the discharge date with the patient, 2) start with arrangement of required post-discharge care within 48 hours after admission; 3) prepare patient handovers (medical, medication, nurse) and personalized patient discharge letter (PPDL[29]) within 48 hours after admission, 4) plan a discharge conversation with the patient to explain information from the PPDL 12-24h before discharge. The TIP bundle was available on checklists for nurses and physicians.

Patient and Public Involvement

Our research question was developed from the perspective that patients are discharged with little coordination or follow-up and that they are often unprepared at time of discharge[4 5]. Patients were involved in the construction of the TIP discharge bundle, which was based on, among others, patient satisfaction surveys[25 28]. Further, in a previous study in which the PPDL was developed and implemented, patient satisfaction with the PPDL was also assessed[29].

Protocol adherence

A process evaluation was conducted with the project leaders to investigate protocol adherence, implementation strategies and attention paid to implementation. Elements that were considered included leadership and education of project leaders, projects group, extent of implementation of the discharge bundle, and education of physicians and nurses. Feedback points were awarded for all elements and for the extent to which the hospital complied to a certain element, e.g., for every person present at the kick off meeting or for every project meeting that was held. When a hospital partly complied to an element, e.g. discharge summaries were provided instead of a PPDL or

For peer review only - http://bmjopen.bmj.com/site/about/guidelines.xhtml

feedback on timely handovers was only provided to nurses, 0.5 feedback points were awarded. It was not possible to evaluate percentages of compliance with discharge conversations, planning discharge dates and arrangement of post-discharge care within 48h since these aspects were not reported in patient records. Hospital policies regarding these elements were assessed.

Outcome measures

Our primary outcome was the number of medical and nursing handovers sent within 24h. Medical handovers also include medication handovers and we considered the time that these handovers were sent to the GP. The median time between discharge and the medical handover was considered as secondary outcome. Further, secondary outcomes were length of hospital stay (LOS) and rates of unplanned readmission within 30 days. Variables were all collected from patient files. Data regarding patient characteristics included: demographics, admission ward and medical data (i.e. presence of polypharmacy, comorbidity,[30] number of hospitalization in the six months prior to current hospitalization). All data were reported and analyzed anonymously.

Sample size calculation

Based on the findings of a previous study[28] we expected to find a reduction of 78% in the time between discharge and medical handovers sent. We conducted a power analysis with a number of patients based on the number of hospital beds at the participating wards and feasibility with regards to data collection, which was set at 11 patients. In a simulation study with 16 wards, each contributing 65 patients, we estimated the power to be approximate 91% to demonstrate a reduction of 78% in time until sending the medical handover, assuming that the intraclass correlation coefficient does not exceed 0.05.

Statistical analysis

For peer review only - http://bmjopen.bmj.com/site/about/guidelines.xhtml

Descriptive characteristics of patients were calculated using proportions, means and standard deviations (SD), or medians and interquartile ranges (IQR), as appropriate. Chi-squared analysis and the Mann Whitney test were used to compare pre-intervention and post-intervention patient characteristics. To analyze the intervention-effect, generalized least square analysis were performed to assess changes in level and slope of the regression lines before and after implementation[24]. A change in level is defined as the difference between the observed level at the first post-intervention time point and that predicted by the pre-intervention time trend. A change in trend is defined as the difference between post- and pre-intervention slopes. We explored models with no, a first order autoregressive correlation between consecutive data collection periods, and longer autocorrelation structures. We used the Akaike Information Criterion (AIC) as an estimator of the relative quality of a model and we report the results from the best fitting model. Correction for baseline imbalances as potential confounders led to results with similar estimates and identical interpretation. Based on the extent of protocol adherence and the feedback points awarded, subgroup analyses were performed to assess the intervention effect on the number of medical handovers within 24h and the median time between discharge and medical handovers. Statistical analyses were performed using SPSS Statistics ©, version 24.0, and Rstudio, version 1.0.136 (© 2009 – 2016 Rstudion, Inc).

Results

A total of 2091 patient records (1039 pre- and 1052 post-intervention) were reviewed in order to investigate the effect of the Transfer Intervention Procedure (TIP) on the timeliness of medical and nursing handovers, length of hospital stay (LOS) and unplanned readmission within 30 days. Overall patients had a mean age (SD) of 68.1 (16.6) and 46.4% were male (table 1). There were significant differences between the pre-, and post-intervention group with regard to polypharmacy and the ratio of acute/elective hospitalizations and these variables were considered as potential

For peer review only - http://bmjopen.bmj.com/site/about/guidelines.xhtml

For peer review only

confounders. However, correction for these potential confounders did not provide better models than the presented models.

Protocol adherence

Implementation strategies and protocol adherence are summarized in Supplement Table 1. Based on the process evaluation, three subgroups were identified. Subgroup 1 (hospitals 4 and 8), >30 feedback points, paid considerable attention to implementation and there was relatively high protocol adherence. In subgroup 2 (hospitals 1-3, and 5), 20-30 feedback points, there was relatively high protocol adherence but moderate attention to implementation. In subgroup 3 (hospitals 6 and 7), <10 feedback points, nearly no attention was brought to implementation and there was low compliance.

Medical and nursing handovers

In the total study population, no intervention effect was found on the percentage of medical handovers being sent within 24h after hospital discharge to the GP: 22.7% medical handovers were sent within 24h pre-intervention, 29.1% post-intervention and no significant difference was observed in the levels and slopes between the pre-intervention and post-intervention period. The median (interquartile range, IQR) time between discharge and medical handovers decreased from 6.15 (0.96-15.96) pre-intervention to 4.08 (0.33-13.67) post-intervention. An absolute effect directly after the implementation of the intervention of -0.25 days was found. No significant difference in the levels and slopes was observed. The number of nursing handovers sent within 24h post-discharge was 92.8% pre-intervention and 93.1% post-intervention and no significant difference was observed between levels and slopes. The results are presented in Figure 2 and the parameters estimates are summarized in Table 2.

For peer review only - http://bmjopen.bmj.com/site/about/guidelines.xhtml

Length of hospital stay and unplanned readmission rates

No significant decline in the levels and slopes between the pre-, and post-intervention was found with regard to LOS (β 0.08, 95% CI -0.12 to 0.29 p=0.45) and unplanned readmission rates (β 1.11, 95% CI -2.55 to 0.33 p=0.17). Median (IQR) LOS was 8.17 (4.75-15.13) and 8.56 (4.88-15.91) days and readmissions rates were as high as 11.1% and 12.3% pre-intervention and post-intervention, respectively.

Subgroup analysis

In subgroup 1 (>30 feedback points), an absolute effect of 13.3% more medical handovers sent within 24h post-discharge was observed but this did not result in significant changes in level or slope (Figure 3). A reduction of 5.6 days in the median time between discharge and handovers with a significant change in level directly after the intervention was observed in subgroup 1 (β -5.29, 95% CI -8.70 to 1.87 p=0.02). Pre-intervention, group 2 (20-30 feedback points) had the highest rate of medical handovers sent within 24h and the lowest median time between discharge and medical handovers but no intervention effect was. Both pre- and post-intervention, subgroup 3 (<10 points) had the lowest rates of medical handovers sent within 24h, and the highest median time. No intervention effect was observed in subgroup 3.

Discussion

In the total study population, a structured discharge bundle, the Transfer Intervention Procedure (TIP), did not lead to improved timeliness of patient handovers. Although medical handovers were sent faster post-intervention (pre-intervention median 6.15; post-intervention 4.08 days), we were unable to show significant differences in level and slopes, both with regard to the median time and the number of medical handovers sent within 24h. However, large inter-hospital variation was observed and a significant intervention effect on the median time between discharge and medical

For peer review only

For peer review only - http://bmjopen.bmj.com/site/about/guidelines.xhtml

handovers was seen in those hospitals with relatively high protocol adherence and attention for implementation. Rates of nursing handovers sent within 24h were both pre- and post-intervention above 90%. No intervention effect was found for length of hospital stay (LOS) and readmissions.

Extensive research has been conducted to improve patient handovers from hospital to home[7 16]. Summarizing findings of earlier discharge interventions that aimed to improve coordination of care and communication between hospital and primary care providers, Hesselink et al.,[7] and Kripalani et al.,[8] showed that some studies were able to improve timeliness of discharge summaries. These interventions, however, were based on the introduction of fax, email or web-based transfers of information, which is increasingly becoming standard practice in Dutch hospitals. Yet, further improvement may lie in electronic sending systems that support the use of standardized formats that pull information from patient files into discharge letters or that send discharge summaries automatically.

While the observed median time between discharge and sending medical handovers at our first pre-intervention measurement point was consistent with a recent Dutch study,[14] a trend towards sending handovers faster was observed along the pre-intervention period. During this period, no interventions were implemented but attention was already brought to the discharge procedure, e.g. by establishing project groups and the kick-off meeting. Since education on the importance of the intervention is an important aspect of implementation,[13 31] this could explain why improvements were already observed.

Although positive trends in the pre-intervention period were less pronounced in the subgroup analysis, results of the separate analyses support the idea that attention is important. Whereas a significant reduction of six days in the median time between discharge and medical handovers was observed in hospitals that paid much attention to implementation, no intervention effect was observed in hospitals that paid moderate to nearly no attention. It should be noted that

For peer review only - http://bmjopen.bmj.com/site/about/guidelines.xhtml

the hospitals that paid moderate attention had relatively good pre-intervention scores. A smaller window for improvement in these hospitals might also explain a lack of intervention effect[32].

Implementation of the TIP procedure did not reveal a reduction of LOS. Although a possible explanation can be low overall compliance with our study protocol, it is also probable that over the past years, average LOS has decreased to a minimum[33]. Given current pressure on availability of hospital beds, patients are discharged as soon as possible. This may account for inadequate discharge processes, since physicians are forced to prioritize acute health care over discharge-related tasks[34 35].

Given increasingly shorter LOS[33] and the often complex care needs patient face, patient preparation should be an important aspect of the discharge process. In fact, the most effective discharge interventions seem to have educational components[36]. Unfortunately, given the workload among residents, implementation of a personalized patient discharge letter was unsuccessful. E.g., posing the question "do you feel ready to go home"[37] or post-discharge telephone contact,[7] might be less time-consuming ways to involve patients. However, to prevent readmissions more effort might be necessary. Previous interventions that revealed a reduction in readmission rates, consist of individualized discharge planning or continue post-discharge[16 38]. However, we believe that a structured discharge process such as the TIP should form the basis for a safe handover for every patient (Figure 1).

Implications for further research

Our study sheds light on the difficulties that come along with implementation of a discharge bundle. A comprehensive exploration of local barriers for each step in the TIP discharge procedure might be helpful in order to develop tailor made interventions on a local or department level[39]. However, given the positive pre-intervention trends and significant reduction in the median time between discharge and medical handovers in hospitals that paid much attention to implementation, further

For peer review only - http://bmjopen.bmj.com/site/about/guidelines.xhtml

improvement of the discharge process may lie in interventions that create more awareness of the importance of timely handovers and continuity of care[35].

Strengths and limitations

The design of the study was a strength of the study. A before-after design would probably have led to a significant intervention-effect, and ITS analysis provided valuable information on pre-intervention trends. Our study has some limitations. We only recorded the date of sending patient handovers. Knowing whether they were received by GPs would also have provided valuable information. Besides, we did not look at the content of handovers, while this might have given us important insights. This also accounts for medication handovers, particularly since medication changes are often missing[14]. Further, information on timeliness and accurateness of medication handovers sent to pharmacists would have been informative but these were mostly sent by fax and these data could not be collected from patient records. Lastly, it was not possible to evaluate percentages of protocol adherence and the process evaluation with the project leaders might have been an overestimation. However, the process evaluation was in line with the efforts observed during implementation.

Conclusion

Implementation of a structured discharge bundle, the Transfer Intervention Procedure (TIP), did not lead to more patient handovers sent within 24h post-discharge. Large inter-hospital variation was observed however, and a significant intervention effect on the median time between discharge and medical handovers was seen in those hospitals that brought much attention to implementation. We believe that future interventions should continue to create awareness of the importance of timely handovers and we hope that our study contributes to this, stimulating hospitals to further structure and improve their discharge process.

For peer review only - http://bmjopen.bmj.com/site/about/guidelines.xhtml

Contributors: BB, SG, and RVS designed the study. BB and SG, conceived the study and obtained funding. RVS collected data and JM and RVS performed statistical analysis. All authors contributed to drafting the manuscript or revised it critically and gave final approval for publication. The authors would like to acknowledge the contribution of the TIP study group, consisting in addition to the authors, of the following members Hanneke Pullens (Catharina Hospital Eindhoven), Barbara van Munster (Gelre Hospitals Apeldoorn), Annemarie van der Lugt (Haven Hospital Rotterdam), Alie Haze-Visser (Lange Land Hospital Zoetermeer), Agnes van 't Hof (Maxima Medical Center Veldhoven), Mariët Dirkzwager (OLVG Amsterdam), Stella de Regt (Reinier de Graaf Hospital Delft), Suzan Vroomen (Academic Medical Center Amsterdam) and Lisette Bruns (Dutch Ministry of Health, Welfare, and Sport). Furthermore, we thank Rachel de Vries en Vera van Miltenburg for their assistance with data collection.

Funding sources: This work was supported by the Dutch Ministry of Health, Welfare and Sport [grant number: 324798].

Conflicts of interest: None declared.

Data sharing statement: No additional data are available.

For peer review only

References

1. Eurostat Statistics Explained. Hospital discharges and length of stay statistics 2016 [Available from: http://ec.europa.eu/eurostat/statistics-explained/index.php/Hospital_discharges_and_length_of_stay_statistics accessed October 17 2017.
2. Jean Hall M, DeFrances CJ, Williams SN, Golonsinskiy MS, Schwartzman A. National Health Statistics Reports. *US Department of Health and Guman Services Centers for Disease Control and Prevention National Center for Health Statistics* 2010;29
3. Boyd CM, Darer J, Boult C, Fried LP, Boult L, Wu AW. Clinical practice guidelines and quality of care for older patients with multiple comorbid diseases: implications for pay for performance. *Jama* 2005;294(6):716-24. doi: 10.1001/jama.294.6.716 [published Online First: 2005/08/11]
4. Henderson A, Zernike W. A study of the impact of discharge information for surgical patients. *Journal of advanced nursing* 2001;35(3):435-41. [published Online First: 2001/08/08]
5. Holland DE, Mistiaen P, Bowles KH. Problems and unmet needs of patients discharged "home to self-care". *Professional case management* 2011;16(5):240-50; quiz 51-2. doi: 10.1097/NCM.0b013e31822361d8 [published Online First: 2011/08/19]
6. Greenwald JL, Jack BW. Preventing the preventable: reducing rehospitalizations through coordinated, patient-centered discharge processes. *Professional case management* 2009;14(3):135-40; quiz 41-2. doi: 10.1097/NCM.0b013e318198d4e1 [published Online First: 2009/05/29]
7. Hesselink G, Schoonhoven L, Barach P, et al. Improving patient handovers from hospital to primary care: a systematic review. *Annals of internal medicine* 2012;157(6):417-28. doi: 10.7326/0003-4819-157-6-201209180-00006 [published Online First: 2012/09/19]
8. Kripalani S, LeFevre F, Phillips CO, Williams MV, Basaviah P, Baker DW. Deficits in communication and information transfer between hospital-based and primary care physicians: implications for patient safety and continuity of care. *Jama* 2007;297(8):831-41. doi: 10.1001/jama.297.8.831 [published Online First: 2007/03/01]
9. Forster AJ, Murff HJ, Peterson JF, Gandhi TK, Bates DW. The incidence and severity of adverse events affecting patients after discharge from the hospital. *Annals of internal medicine* 2003;138(3):161-7. [published Online First: 2003/02/01]
10. Moore C, Wisnivesky J, Williams S, McGinn T. Medical errors related to discontinuity of care from an inpatient to an outpatient setting. *Journal of general internal medicine* 2003;18(8):646-51. [published Online First: 2003/08/13]
11. Fluitman KS, van Galen LS, Merten H, et al. Exploring the preventable causes of unplanned readmissions using root cause analysis: Coordination of care is the weakest link. *European journal of internal medicine* 2016;30:18-24. doi: 10.1016/j.ejim.2015.12.021 [published Online First: 2016/01/18]
12. Kansagara D, Englander H, Salanitro A, et al. Risk prediction models for hospital readmission: a systematic review. *Jama* 2011;306(15):1688-98. doi: 10.1001/jama.2011.1515 [published Online First: 2011/10/20]
13. Merten H, van Galen LS, Wagner C. Safe handover. *BMJ (Clinical research ed)* 2017;359:j4328. doi: 10.1136/bmj.j4328 [published Online First: 2017/10/11]
14. Langelaan M, Baines RJ, de Bruijne MC, Wagner C. Association of admission and patient characteristics with quality of discharge letters: posthoc analysis of a retrospective study. *BMC health services research* 2017;17(1):225. doi: 10.1186/s12913-017-2149-8 [published Online First: 2017/03/23]
15. Dharmarajan K, Hsieh AF, Lin Z, et al. Diagnoses and timing of 30-day readmissions after hospitalization for heart failure, acute myocardial infarction, or pneumonia. *Jama* 2013;309(4):355-63. doi: 10.1001/jama.2012.216476 [published Online First: 2013/01/24]

For peer review only - http://bmjopen.bmj.com/site/about/guidelines.xhtml

16. Verhaegh KJ, MacNeil-Vroomen JL, Eslami S, Geerlings SE, de Rooij SE, Buurman BM. Transitional care interventions prevent hospital readmissions for adults with chronic illnesses. *Health affairs (Project Hope)* 2014;33(9):1531-9. doi: 10.1377/hlthaff.2014.0160 [published Online First: 2014/09/10]
17. Prvu Bettger J, Alexander KP, Dolor RJ, et al. Transitional care after hospitalization for acute stroke or myocardial infarction: a systematic review. *Annals of internal medicine* 2012;157(6):407-16. doi: 10.7326/0003-4819-157-6-201209180-00004 [published Online First: 2012/09/19]
18. Shepperd S, Lannin NA, Clemson LM, McCluskey A, Cameron ID, Barras SL. Discharge planning from hospital to home. *The Cochrane database of systematic reviews* 2013;1:Cd000313. doi: 10.1002/14651858.CD000313.pub4 [published Online First: 2013/02/27]
19. Buurman BM, Parlevliet JL, Allore HG, et al. Comprehensive Geriatric Assessment and Transitional Care in Acutely Hospitalized Patients: The Transitional Care Bridge Randomized Clinical Trial. *JAMA internal medicine* 2016;176(3):302-9. doi: 10.1001/jamainternmed.2015.8042 [published Online First: 2016/02/18]
20. Naylor MD, Brooten DA, Campbell RL, Maislin G, McCauley KM, Schwartz JS. Transitional care of older adults hospitalized with heart failure: a randomized, controlled trial. *Journal of the American Geriatrics Society* 2004;52(5):675-84. doi: 10.1111/j.1532-5415.2004.52202.x [published Online First: 2004/04/17]
21. The Joint Commission. Hot Topics in Health Care. Transitions of Care: The need for a more effective approach to continuing patient care. *The Joint Commission* 2012
22. Kripalani S, Jackson AT, Schnipper JL, Coleman EA. Promoting effective transitions of care at hospital discharge: a review of key issues for hospitalists. *Journal of hospital medicine* 2007;2(5):314-23. doi: 10.1002/jhm.228 [published Online First: 2007/10/16]
23. Jandoc R, Burden AM, Mamdani M, Levesque LE, Cadarette SM. Interrupted time series analysis in drug utilization research is increasing: systematic review and recommendations. *Journal of clinical epidemiology* 2015;68(8):950-6. doi: 10.1016/j.jclinepi.2014.12.018 [published Online First: 2015/04/22]
24. Penfold RB, Zhang F. Use of interrupted time series analysis in evaluating health care quality improvements. *Academic pediatrics* 2013;13(6 Suppl):S38-44. doi: 10.1016/j.acap.2013.08.002 [published Online First: 2013/12/07]
25. van Seben R, Geerlings SE, Verhaegh KJ, Hilders CG, Buurman BM. Implementation of a Transfer Intervention Procedure (TIP) to improve handovers from hospital to home: interrupted time series analysis. *BMC health services research* 2016;16:479. doi: 10.1186/s12913-016-1730-x [published Online First: 2016/09/09]
26. Davidoff F, Batalden P, Stevens D, Ogrinc G, Mooney SE. Publication guidelines for quality improvement studies in health care: evolution of the SQUIRE project. *BMJ (Clinical research ed)* 2009;338:a3152. doi: 10.1136/bmj.a3152 [published Online First: 2009/01/21]
27. Dutch Health Care Inspectorate (IGZ). Continuïteit van zorg voor kwetsbare ouderen vanuit het ziekenhuis naar verpleeg- en verzorgingshuizen, thuiszorg en huisartsen niet gewaarborgd. . 2015
28. Verhaegh KJ, Buurman BM, Veenboer GC, de Rooij SE, Geerlings SE. The implementation of a comprehensive discharge bundle to improve the discharge process: a quasi-experimental study. *The Netherlands journal of medicine* 2014;72(6):318-25. [published Online First: 2014/10/17]
29. Buurman BM, Verhaegh KJ, Smeulers M, et al. Improving handoff communication from hospital to home: the development, implementation and evaluation of a personalized patient discharge letter. *International journal for quality in health care : journal of the International Society for Quality in Health Care / ISQua* 2016;28(3):384-90. doi: 10.1093/intqhc/mzw046 [published Online First: 2016/05/27]

For peer review only - http://bmjopen.bmj.com/site/about/guidelines.xhtml

30. Charlson ME, Pompei P, Ales KL, MacKenzie CR. A new method of classifying prognostic comorbidity in longitudinal studies: development and validation. *Journal of chronic diseases* 1987;40(5):373-83. [published Online First: 1987/01/01]
31. Michie S, van Stralen MM, West R. The behaviour change wheel: a new method for characterising and designing behaviour change interventions. *Implementation science : IS* 2011;6:42. doi: 10.1186/1748-5908-6-42 [published Online First: 2011/04/26]
32. Ivers N, Jamtvedt G, Flottorp S, et al. Audit and feedback: effects on professional practice and healthcare outcomes. *The Cochrane database of systematic reviews* 2012(6):Cd000259. doi: 10.1002/14651858.CD000259.pub3 [published Online First: 2012/06/15]
33. Coppa Consultancy. Ligduurmonitor Nederlandse ziekenhuizen 2013 & 2014. Trendbreuk in de ligduur. 2015 [Available from: http://www.coppa.nl/wp-content/uploads/2016/01/Ligduurmonitor-2013-2014.pdf accessed November, 2017.
34. Greysen SR, Schiliro D, Horwitz LI, Curry L, Bradley EH. "Out of sight, out of mind": housestaff perceptions of quality-limiting factors in discharge care at teaching hospitals. *Journal of hospital medicine* 2012;7(5):376-81. doi: 10.1002/jhm.1928 [published Online First: 2012/03/02]
35. Hesselink G, Vernooij-Dassen M, Pijnenborg L, et al. Organizational culture: an important context for addressing and improving hospital to community patient discharge. *Medical care* 2013;51(1):90-8. doi: 10.1097/MLR.0b013e31827632ec [published Online First: 2012/11/08]
36. Mistiaen P, Francke AL, Poot E. Interventions aimed at reducing problems in adult patients discharged from hospital to home: a systematic meta-review. *BMC health services research* 2007;7:47. doi: 10.1186/1472-6963-7-47 [published Online First: 2007/04/06]
37. van Galen LS, Brabrand M, Cooksley T, et al. Patients' and providers' perceptions of the preventability of hospital readmission: a prospective, observational study in four European countries. *BMJ quality & safety* 2017 doi: 10.1136/bmjqs-2017-006645 [published Online First: 2017/06/24]
38. Leppin AL, Gionfriddo MR, Kessler M, et al. Preventing 30-day hospital readmissions: a systematic review and meta-analysis of randomized trials. *JAMA internal medicine* 2014;174(7):1095-107. doi: 10.1001/jamainternmed.2014.1608 [published Online First: 2014/05/14]
39. Flottorp SA, Oxman AD, Krause J, et al. A checklist for identifying determinants of practice: a systematic review and synthesis of frameworks and taxonomies of factors that prevent or enable improvements in healthcare professional practice. *Implementation science : IS* 2013;8:35. doi: 10.1186/1748-5908-8-35 [published Online First: 2013/03/26]
40. van Seben R, Geerlings SE, Buurman BM. [10 tips for improving patient handovers from hospital to other health care settings]. *Ned Tijdschr Geneeskd* 2017;161(0):D1031. [published Online First: 2017/04/27]

For peer review only - http://bmjopen.bmj.com/site/about/guidelines.xhtml

Table 1. Baseline characteristics

Variable	Overall (N=2091)	Pre-intervention (N=1039)	Post-intervention (N=1052)
**Age in years,** mean (SD)[a]	68.07 (16.57)	67.66 (16.70)	68.48 (16.45)
**Male,** No. (%)	971 (46.4)	493 (47.4)	478 (45.4)
**Living arrangements before admission,** No.(%)			
Independent	1814 (86.7)	883 (84.9)	931 (88.5)
Nursing home	49 (2.3)	27 (2.6)	22 (2.1)
Senior residence/Assisted living	168 (8.1)	91 (8.8)	77 (7.3)
Missing	60 (2.9)	38 (3.7)	22 (2.1)
**Marital status,** No. **(%)**			
Married or living together	1125 (53.8)	556 (53.5)	569 (54.1)
Single or divorced	456 (21.8)	212 (20.4)	244 (23.2)
Widow/widower	435 (20.8)	224 (21.6)	211 (20.1)
Missing	75 (3.6)	47 (4.5)	28 (2.7)
**Charlson Comorbidity Index** [b] (mean, SD [a])	2.05 (2.05)	2.10 (2.08)	2.01 (2.03)
**Polypharmacy,** No. (%)[c, d, e]	1247 (59.6)	586 (56.4)	661 (62.8)
Missing	12 (.6)	8 (.8)	4 (.4)
**Hospitalization in past 6 months,** No. (%)	705 (33.7)	339 (32.6)	336 (34.8)
**Acute hospitalization,** No. (%)[c, f]	73.0 (73.0)	725 (69.8)	801 (76.1)
**Admission ward, internal medicine** No. (%)	1051 (50.3)	524 (50.4)	527 (50.1)
**Discharge destination,** No. (%)			
Home	1551 (74.2)	770 (74.1)	781 (74.2)
Other health care setting, of which	482 (23.1)	238 (23.0)	244 (23.2)
Rehabilitation center	268 (12.8)	120 (11.5)	148 (14.1)
Nursing home	158 (7.6)	80 (7.7)	78 (7.4)
Assisted living	34 (1.6)	26 (2.5)	8 (0.8)
Other hospital	22 (1.1)	12 (1.2)	10 (1.0)
Missing	58 (2.8)	31 (3.0)	27 (2.6)

[a] Standard Deviation, [b] Range of 0 to 31, with a higher score indicating more or more severe comorbidity[30], [c] Use of 5 or more different medications, [d] Chi-Square, [e] P-value = 0.004, [f] P-value = 0.001

For peer review only - http://bmjopen.bmj.com/site/about/guidelines.xhtml

Table 2. Interrupted time series analysis; medical and nursing handovers

	Medical handovers <24 hrs after discharge (%) [a]			*Time between discharge and medical letter (days)* [b]			*Nursing handovers <24 hrs after discharge (%)* [a]		
	**β (SE)**	**95% CI**	**p-value**	**β (SE)**	**95% CI**	**p-value**	**β (SE)**	**95% CI**	**p-value**
Intercept	17.51 (3.79)	10.08 to 24.93	<0.01	7.20 (0.29)	6.63 to 7.76	<0.01	91.85 (2.71)	86.53 to 97.16	<0.01
Trend pre-intervention (β1)	1.49 (0.97)	-0.42 to 3.40	0.16	-0.30 (0.07)	-0.45 to -0.16	<0.01	0.28 (0.70)	-1.09 to 1.64	0.70
Level change directly after intervention (β2)	6.43 (10.13)	-13.43 to 26.28	0.54	-0.62 (0.74)	-2.07 to 0.84	0.43	6.32 (7.25)	-7.89 to 20.53	0.41
Trend differences (β3)	-0.94 (1.38)	-3.64 to 1.75	0.51	0.05 (0.10)	-0.14 to 0.25	0.61	-0.81 (0.99)	-2.74 to 1.12	0.43
	Absolute effect directly after intervention: -0.17%			Absolute effect directly after intervention: -0.25 days			Absolute effect directly after intervention: 0.62%		

β1 estimates the pre-intervention slope.
β2 estimates the difference between the observed level just after the intervention started and that predicted by the pre-intervention slope.
β3 estimates the difference in trend/slopes between the pre-intervention and post-intervention period.
SE: Standard Error, CI: Confidence Interval

For peer review only

For peer review only - http://bmjopen.bmj.com/site/about/guidelines.xhtml

Figure 1. Pyramid for post-discharge care

A structured discharge process such as the TIP procedure should form the basis for every patient. For patients discharged with post-discharge care (20-25%), nursing handovers should be set up within 48h after admission and be sent within 24h post-discharge. Complex patients with a high readmission risk (10%) require a (nurse) case manager or transitional care in the transition from hospital to home. Adapted from van Seben et al.[40]

Figure 2.

Panel A The number of medical handovers sent within 24 hours.

Panel B median time in days between discharge and the medical handovers.

Figure 3. Hospital differences based on implementation score.

The inter-hospital differences in rates of medical discharge letters being sent within 24h in the pre- and post-intervention based on the extent of implementation and used implementation strategies. Group 1 received >30 feedback implementation points, group 2 received 20-30 implementation points, group 3 received <20 points.

For peer review only - http://bmjopen.bmj.com/site/about/guidelines.xhtml

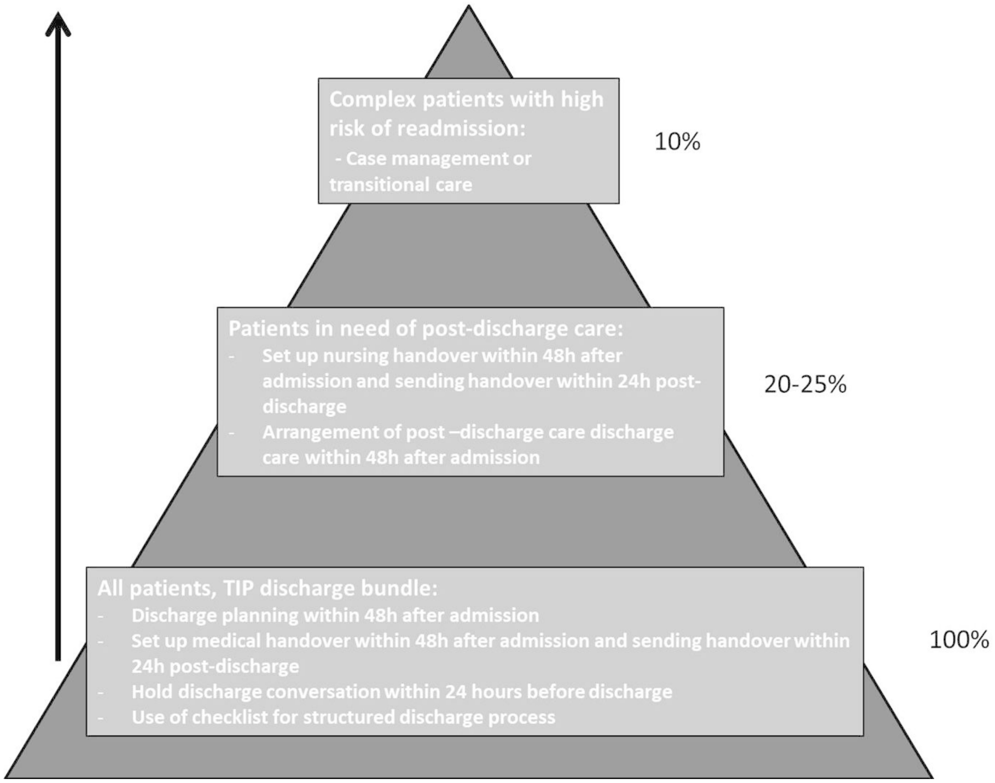

Figure 1. Pyramid for post-discharge care
A structured discharge process such as the TIP procedure should form the basis for every patient. For patients discharged with post-discharge care (20-25%), nursing handovers should be set up within 48h after admission and be sent within 24h post-discharge. Complex patients with a high readmission risk (10%) require a (nurse) case manager or transitional care in the transition from hospital to home. Adapted from van Seben et al.40

116x92mm (300 x 300 DPI)

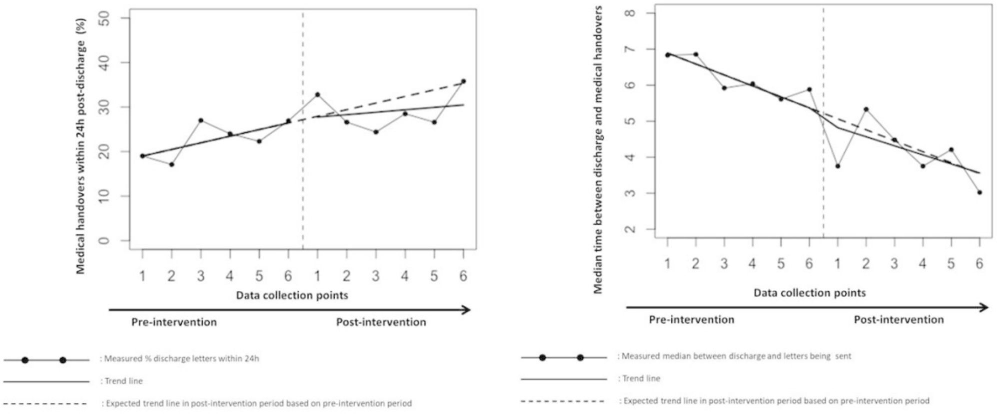

Figure 2.
Panel A The number of medical handovers sent within 24 hours.
Panel B median time in days between discharge and the medical handovers.

113x50mm (300 x 300 DPI)

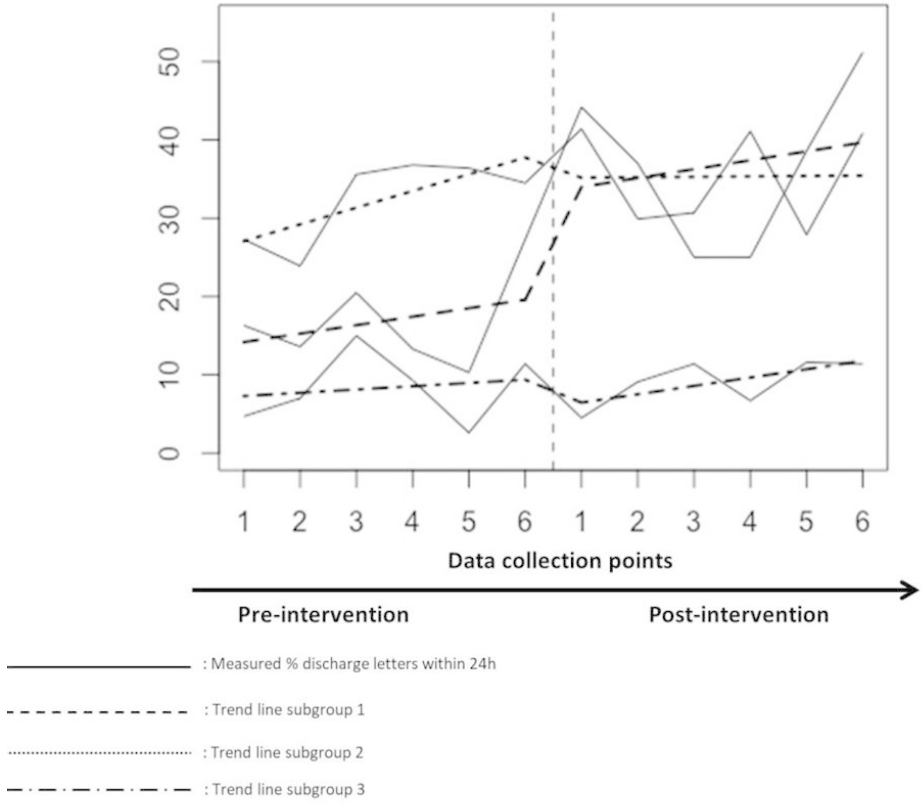

Figure 3. Hospital differences based on implementation score.
The inter-hospital differences in rates of medical discharge letters being sent within 24h in the pre- and post-intervention based on the extent of implementation and used implementation strategies. Group 1 received >30 feedback implementation points, group 2 received 20-30 implementation points, group 3 received <20 points.

82x69mm (300 x 300 DPI)

Supplement Table 1. Adherence to the Intervention Protocol

	Hospital 1	Hospital 2	Hospital 3	Hospital 4	Hospital 5	Hospital 6	Hospital 7	Hospital 8
Pre-intervention<br>Implementation<br>Post-intervention	Sep '15 - Feb '16<br>March '16 - April '16<br>May '16 – Oct '16	Oct '15 - March '16<br>April '16 - May '16<br>June '16 - Nov '16	Jan '16 - June '16<br>July '16 - Aug '16<br>Sep '16 – Feb '17	Dec '15 - May '16<br>June '16 - July '16<br>Aug '16 - Jan '16	March '16 – Aug '16<br>Sep '16 - Oct '16<br>Nov '16 – April '17	April '16 – Sep '16<br>Oct '16 - Nov '16<br>Dec '16 – May '17	May '16 – Oct '16<br>Nov '16 - Dec '16<br>Jan '17 – June '17	April '16 – Sep '16<br>Oct '16 - Nov '16<br>Dec '16 – May '17
**Leadership and education of project leaders**								
Who were present at the kick off meeting February, 2016?	Hospital president; local project leader; 2 team leaders (nurses); 2 physicians; nurse; pharmacists	Local project leader; geriatrician; head of the liaison department; physician	2 local project leaders; head of the liaison department	Chief of staff; local project leader; team leader surgery ward (nurse); head of the liaison department	Local project leader; head of the liaison department; manager patient logistics; 2 team leaders (nurses)	2 local project leaders	Local project leader	Local project leader; senior researcher transitional care; medical specialist,
Who were present at the first feedback session?	Project leader; head of the liaison department	Project leader; pharmacist; communication assistant	2 local project leaders; liaison nurse	Project leader; liaison nurse; nurse geriatrics	local project leader	2 local project leaders	-	2 project leaders
Who were present at the second feedback session?	Project leader	Project leader	2 project leaders	-	Project leader	-	Project leader	2 project leaders
Implementation points	*10*	*8*	*8*	*7*	*7*	*4*	*2*	*7*
**Project group**								
Was there a local TIP project group, and who participated?	Yes, project leader; 2 senior nurses of participating wards, management assistant	Yes, project leader; geriatrician; head liaison department, physician; pharmacist; communication assistant; manager Security & Services	Yes, 2 project leaders; 2 residents; 2 medical specialists; nurse; liaison nurse; pharmacist; manager	Yes, chief of staff; project leader; 2 team leaders (nurses); head liaison department; orthopedist	Yes, project leader; head liaison department; 2 medical specialists, geriatrician	No	No	Yes, local project leader; 3 medical specialists; 2 residents, manager quality and safety; manager process optimization; medical director
How often did the local project group meet?<br>1 point per meeting	Monthly for 2 months, during pilot period every week (2 months).	Every five weeks during pre-intervention and pilot period	2 times, before pilot period.	Every two weeks during pre-intervention period	Every six weeks, during pre-intervention and pilot period	-	-	Monthly during pre-intervention, pilot period and first two months of post-

For peer review only

								intervention period
Implementation points	*10*	*12*	*10*	*18*	*10*	*0*	*0*	*17*
**Implementation of TIP elements**								
Was it policy to set a discharge date within 48h after admission?	Yes	Yes	Yes	Yes	Yes	Yes	Yes	Yes
Was the planned discharge date communicated to patients?	Yes	Yes	Yes	Yes	Yes	No	Yes	Yes
Was it policy to start with arrangement of required post-discharge care within 48h after admission?	No, liaison nurse has to wait for final discharge date	No, liaison nurse has to wait for final discharge date	Yes	Yes	No, liaison department is overloaded	Yes	No, liaison nurse has to wait for final discharge date	Yes
Was it policy to set up patient handovers within 48h after admission?	No	No	No	No	No	No	No	No
Did physicians hold discharge conversations, using a checklist during the pilot period?	No	No	No	No	No	No	No	No
Does the nurse holds a discharge conversation, using a checklist?	Yes	No	Yes	Yes	Yes	No	Yes	Yes
Was the patient discharge letter implemented?	Yes, but only for some diagnosis at internal medicine ward.	No	No	No	No	Yes , but only at internal medicine ward for frail older patients.	No	No, a discharge summary was implemented instead.
Implementation points	*3.5*	*2*	*4*	*4*	*3*	*2.5*	*3*	*4.5*
**Education of physicians and nurses**								

For peer review only

How were physicians and nurses informed about the TIP and how often?	Kick-off meeting at participating wards; during morning report; working instructions were sent by email to all physicians		Kick-off meeting; meeting at participating wards; E-learning; 1 feedback meeting		During morning reports; project leader informed every physician separately; intranet; email		During morning reports; intranet; email; posters & pocket cards; and project leaders went to participating wards to inform physicians and nurses		Email and project leader went to participating wards		Kick-off meeting, during several morning reports		Physicians were not educated with regard to the intervention		During several morning report; email; project leaders went to participating wards to inform physicians and nurses; medical specialists from project group informed physicians in person	
Did physicians and/or nurses receive feedback with regard to their discharge letters and if yes, how often?	No.		No		No		No		No		Only for nurses		No		Yes, daily on internal medicine and monthly on surgery ward, via email.	
Implementation points	*3*		*4*		*4*		*5*		*2*		*2.5*		*0*		*5*	
**Total implementation points**	**26.5**		**26**		**26**		**34**		**22**		**9**		**5**		**33.5**	
**Pre-intervention vs. post-intervention period scores**																
**Pre-intervention period**																
median / % letters within 24h	8.15	9.0	0.90	47.3	6.71	23.5	10.48	13.1	0.79	50.0	6.79	9.2	14.21	7.6	5.83	20.9
**Post-intervention**																
median / % letters within 24h	9.08	19.5	1.0	48.5	5.48	24.2	5.79	19.7	0.29	49.6	7.98	16.7	22.44	1.5	0.83	53.8

For peer review only

Revised Standards for Quality Improvement Reporting Excellence (SQUIRE 2.0)
September 15, 2015

Text Section and Item Name	Section or Item Description
**Notes to authors**	• The SQUIRE guidelines provide a framework for reporting new knowledge about how to improve healthcare<br><br>• The SQUIRE guidelines are intended for reports that describe system level work to improve the quality, safety, and value of healthcare, and used methods to establish that observed outcomes were due to the intervention(s).<br><br>• A range of approaches exists for improving healthcare. SQUIRE may be adapted for reporting any of these.<br><br>• Authors should consider every SQUIRE item, but it may be inappropriate or unnecessary to include every SQUIRE element in a particular manuscript.<br><br>• The SQUIRE Glossary contains definitions of many of the key words in SQUIRE.<br><br>• The Explanation and Elaboration document provides specific examples of well-written SQUIRE items, and an in-depth explanation of each item.<br><br>• Please cite SQUIRE when it is used to write a manuscript.
**Title and Abstract**	
**1. Title**	Indicate that the manuscript concerns an initiative to improve healthcare (broadly defined to include the quality, safety, effectiveness, patient-centeredness, timeliness, cost, efficiency, and equity of healthcare)
**2. Abstract**	a. Provide adequate information to aid in searching and indexing<br>b. Summarize all key information from various sections of the text using the abstract format of the intended publication or a structured summary such as: background, local problem, methods, interventions, results, conclusions
**Introduction**	*Why did you start?*
**3. Problem Description**	Nature and significance of the local problem
**4. Available knowledge**	Summary of what is currently known about the problem, including relevant previous studies

5. Rationale	Informal or formal frameworks, models, concepts, and/or theories used to explain the problem, any reasons or assumptions that were used to develop the intervention(s), and reasons why the intervention(s) was expected to work
6. Specific aims	Purpose of the project and of this report
**Methods**	*What did you do?*
7. Context	Contextual elements considered important at the outset of introducing the intervention(s)
8. Intervention(s)	a. Description of the intervention(s) in sufficient detail that others could reproduce it<br>b. Specifics of the team involved in the work
9. Study of the Intervention(s)	a. Approach chosen for assessing the impact of the intervention(s)<br>b. Approach used to establish whether the observed outcomes were due to the intervention(s)
10. Measures	a. Measures chosen for studying processes and outcomes of the intervention(s), including rationale for choosing them, their operational definitions, and their validity and reliability<br>b. Description of the approach to the ongoing assessment of contextual elements that contributed to the success, failure, efficiency, and cost<br>c. Methods employed for assessing completeness and accuracy of data
11. Analysis	a. Qualitative and quantitative methods used to draw inferences from the data<br>b. Methods for understanding variation within the data, including the effects of time as a variable
12. Ethical Considerations	Ethical aspects of implementing and studying the intervention(s) and how they were addressed, including, but not limited to, formal ethics review and potential conflict(s) of interest
**Results**	*What did you find?*
13. Results	a. Initial steps of the intervention(s) and their evolution over time (*e.g.*, time-line diagram, flow chart, or table), including modifications made to the intervention during the project<br>b. Details of the process measures and outcome<br>c. Contextual elements that interacted with the intervention(s)<br>d. Observed associations between outcomes, interventions, and relevant contextual elements<br>e. Unintended consequences such as unexpected benefits, problems, failures, or costs associated with the intervention(s).<br>f. Details about missing data
**Discussion**	*What does it mean?*
14. Summary	a. Key findings, including relevance to the rationale and specific aims<br>b. Particular strengths of the project

For peer review only

**15. Interpretation**	a. Nature of the association between the intervention(s) and the outcomes<br>b. Comparison of results with findings from other publications<br>c. Impact of the project on people and systems<br>d. Reasons for any differences between observed and anticipated outcomes, including the influence of context<br>e. Costs and strategic trade-offs, including opportunity costs
**16. Limitations**	a. Limits to the generalizability of the work<br>b. Factors that might have limited internal validity such as confounding, bias, or imprecision in the design, methods, measurement, or analysis<br>c. Efforts made to minimize and adjust for limitations
**17. Conclusions**	a. Usefulness of the work<br>b. Sustainability<br>c. Potential for spread to other contexts<br>d. Implications for practice and for further study in the field<br>e. Suggested next steps
**Other information**	
**18. Funding**	Sources of funding that supported this work. Role, if any, of the funding organization in the design, implementation, interpretation, and reporting

For peer review only

Table 2. Glossary of key terms used in SQUIRE 2.0. This Glossary provides the intended meaning of selected words and phrases as they are used in the SQUIRE 2.0 Guidelines. They may, and often do, have different meanings in other disciplines, situations, and settings.

Assumptions
Reasons for choosing the activities and tools used to bring about changes in healthcare services at the system level.

Context
Physical and sociocultural makeup of the local environment (for example, external environmental factors, organizational dynamics, collaboration, resources, leadership, and the like), and the interpretation of these factors ("sense-making") by the healthcare delivery professionals, patients, and caregivers that can affect the effectiveness and generalizability of intervention(s).

Ethical aspects
The value of system-level initiatives relative to their potential for harm, burden, and cost to the stakeholders. Potential harms particularly associated with efforts to improve the quality, safety, and value of healthcare services include opportunity costs, invasion of privacy, and staff distress resulting from disclosure of poor performance.

Generalizability
The likelihood that the intervention(s) in a particular report would produce similar results in other settings, situations, or environments (also referred to as external validity).

Healthcare improvement
Any systematic effort intended to raise the quality, safety, and value of healthcare services, usually done at the system level. We encourage the use of this phrase rather than "quality improvement," which often refers to more narrowly defined approaches.

Inferences
The meaning of findings or data, as interpreted by the stakeholders in healthcare services – improvers, healthcare delivery professionals, and/or patients and families

Initiative
A broad term that can refer to organization-wide programs, narrowly focused projects, or the details of specific interventions (for example, planning, execution, and assessment)

Internal validity
Demonstrable, credible evidence for efficacy (meaningful impact or change) resulting from introduction of a specific intervention into a particular healthcare system.

Intervention(s)
The specific activities and tools introduced into a healthcare system with the aim of changing its performance for the better. Complete description of an intervention includes its inputs, internal activities, and outputs (in the form of a logic model, for example), and the mechanism(s) by which these components are expected to produce changes in a system's performance.

Opportunity costs

For peer review only

Loss of the ability to perform other tasks or meet other responsibilities resulting from the diversion of resources needed to introduce, test, or sustain a particular improvement initiative

Problem
Meaningful disruption, failure, inadequacy, distress, confusion or other dysfunction in a healthcare service delivery system that adversely affects patients, staff, or the system as a whole, or that prevents care from reaching its full potential

Process
The routines and other activities through which healthcare services are delivered

Rationale
Explanation of why particular intervention(s) were chosen and why it was expected to work, be sustainable, and be replicable elsewhere.

Systems
The interrelated structures, people, processes, and activities that together create healthcare services for and with individual patients and populations. For example, systems exist from the personal self-care system of a patient, to the individual provider-patient dyad system, to the microsystem, to the macrosystem, and all the way to the market/social/insurance system. These levels are nested within each other.

Theory or theories
Any "reason-giving" account that asserts causal relationships between variables (causal theory) or that makes sense of an otherwise obscure process or situation (explanatory theory). Theories come in many forms, and serve different purposes in the phases of improvement work. It is important to be explicit and well-founded about any informal and formal theory (or theories) that are used.

For peer review only

BMJ Open

A safe handover for every patient: an interrupted time series analysis to test the effect of a structured discharge bundle

Journal:	*BMJ Open*
Manuscript ID	bmjopen-2018-023446.R1
Article Type:	Research
Date Submitted by the Author:	10-Sep-2018
Complete List of Authors:	van Seben, Rosanne; AMC, Internal Medicine, section of Geriatric Medicine<br>Geerlings, Suzanne; AMC, Internal Medicine, Division of Infectious Diseases<br>Maaskant, Jolanda; AMC, Emma Children's Hospital<br>Buurman, Bianca; Academic Medical Center, Internal Medicine, section of Geriatric Medicine
**Primary Subject Heading**:	Health services research
Secondary Subject Heading:	Communication, Health services research
Keywords:	Discharge Bundle, Patient Handovers, Discharge Letter, Patient Safety, Interrupted Time Series, Quality Improvement

SCHOLARONE™
Manuscripts

A safe handover for every patient: an interrupted time series analysis to test the effect of a structured discharge bundle

Rosanne van Seben (corresponding author)

Amsterdam UMC, University of Amsterdam, Department of Internal Medicine, Section of Geriatric Medicine, Amsterdam Public Health research institute, Meibergdreef 9, Amsterdam, The Netherlands

r.vanseben@amc.uva.nl, +312 56 61647

Suzanne E. Geerlings

Amsterdam UMC, University of Amsterdam, Department of Internal Medicine, Section of Infectious Diseases, Meibergdreef 9, Amsterdam, The Netherlands

Jolanda M. Maaskant

Amsterdam UMC, University of Amsterdam, Emma Children's Hospital, Meibergdreef 9, Amsterdam, The Netherlands

Bianca M. Buurman

Amsterdam UMC, University of Amsterdam, Department of Internal Medicine, Section of Geriatric Medicine, Amsterdam Public Health research institute, Meibergdreef 9, Amsterdam, The Netherlands & ACHIEVE Centre of Expertise, Faculty of Health, Amsterdam University of Applied Sciences, Amsterdam, the Netherlands

On behalf of the TIP study group: Hanneke Pullens, Barbara van Munster, Bianka Mennema, Alie Haze-Visser, Agnes van 't Hof, Mariët Dirkzwager, Stella de Regt, Lisette Bruns, Suzan Vroomen, Jolanda Maaskant, Rosanne van Seben, Suzanne Geerlings and Bianca Buurman

Key words: Discharge Bundle, Patient Handovers, Patient Safety, Interrupted Time Series, Quality Improvement

Word count: 3214

For peer review only - http://bmjopen.bmj.com/site/about/guidelines.xhtml

Abstract

Objective Patient handovers are often delayed, patients are hardly involved in their discharge process and hospital-wide standardized discharge procedures are lacking. The aim of this study was to implement a structured discharge bundle and to test the effect on timeliness of medical and nursing handovers.

Design Interrupted time series with six pre-intervention and six post-intervention data collection points (September 2015 through June 2017).

Setting Internal medicine and surgical wards.

Participants Patients (≥18 years) admitted for more than 48h to surgical or internal medicine wards.

Intervention The Transfer Intervention Procedure (TIP), containing four elements: planning the discharge date within 48h post-admission, arrangements for post-discharge care, preparing handovers and personalized patient discharge letter; and a discharge conversation 12-24h before discharge.

Outcome measures The number of medical and nursing handovers sent within 24h. Secondary outcomes were median time between discharge and medical handovers, length of hospital stay (LOS) and unplanned readmissions.

Results Pre-intervention 1039 and post-intervention 1052 patient records were reviewed. No significant change in levels and slopes was observed in the number of medical and nursing handovers sent within 24h. The median (interquartile range) time between discharge and medical handovers decreased from 6.15 (0.96-15.96) to 4.08 (0.33-13.67) days, but no significant difference in levels and slopes was found. No intervention-effect was observed for LOS and readmission. In subgroup analyses, a reduction of 5.6 days in the median time between discharge and medical handovers was observed in hospitals with high protocol adherence and much attention for implementation.

Conclusion Implementation of a structured discharge bundle did not lead to improved timeliness of patient handovers. However, large inter-hospital variation was observed and an intervention effect

For peer review only - http://bmjopen.bmj.com/site/about/guidelines.xhtml

on the median time between discharge and medical handovers was seen in hospitals with high protocol adherence. Future interventions should continue to create awareness of the importance of timely handovers.

Trial Registration: Dutch Trial Registry: NTR5951

Strengths and limitations of this study

- This study aimed to implement a structured discharge bundle to improve patient handovers for every patient.
- The study design, i.e. Interrupted Time Series Analysis, provided valuable information on pre-intervention trends, which strengthens the results.
- Sensitivity analysis provided important insight into the inter-hospital variation and differences in intervention effects among hospitals.
- Only the date of sending patient handovers were recorded. Knowing whether the next care provider received information would have been informative.
- It was not possible to evaluate percentages of compliance with the study protocol. Therefore, the process evaluation with the project leaders might have been an overestimation.

For peer review only

For peer review only - http://bmjopen.bmj.com/site/about/guidelines.xhtml

Introduction

As hospital stays have become shorter and full recovery often takes place at home,[1] a safe transition from hospital to home or nursing home has become more and more important. Besides, a rising number of older chronically ill patients who move within the health care system, requires continuity of care[2 3]. However, transitions from hospital to primary care settings are still considered a high-risk process. Patients are discharged with little coordination or follow-up and are hardly involved in their own discharge process[4 5].

Inadequate transitions may have serious implications for patient safety and quality of care. Post-discharge adverse events such as medication errors, can be the consequence of insufficient or lacking communication between hospital and primary care providers, thereby contributing to higher resource use and unplanned readmission rates[6-11]. In fact, unplanned readmission rates in the first month post-discharge are as high as 20%[12] and a recent study shows that half of them are deemed preventable[11].

The root of a safe transition from hospital to home or nursing home is a timely transfer of the medical handover, that is a letter containing accurate medical discharge information for the next care provider[8 13]. The general practitioner (GP) can only take over responsibility for a patient safely, when receiving a medical handover containing accurate information on, e.g., medications, and follow-up[13]. Nonetheless, a review of Kripalani et al. showed that medical handover are often not available, lack important information or are not sent in a timely manner[8]. Also, a more recent study performed in 20 Dutch hospitals showed that in 10% of cases medical handover were missing and the remainder was on average sent after one week,[14] even though unplanned readmissions most frequently occur within the first week post-discharge[15].

Previous studies that aimed to improve patient handovers, mainly focused on specific high risk populations and targeted patient-related factors[16-18]. Although such interventions on individualized discharge planning or transitional care have been effective in reducing readmission[16 17]

For peer review only - http://bmjopen.bmj.com/site/about/guidelines.xhtml

and post-discharge mortality rates,[18-20] organizational factors that form the basis of a safe handover should also be optimally arranged[13 21]. In fact, in order to ensure patient safety and continuity of care, early discharge planning, a structured discharge process and timely handovers might be essential[13 21 22]. Besides, given that patients are often unprepared at time of discharge and uncertainties about aspects such as treatment or medication may exist,[5] patient education, e.g., in terms of a proper discharge conversation, should also be an important aspect of the discharge process[6 7].

The aim of this study was, therefore, to implement a structured discharge process, the Transfer Intervention Procedure (TIP), in eight hospitals. The TIP contains four elements: planning the discharge date within 48h after admission, arrangements for required post-discharge care, preparing medical, medication, and nursing handovers and a personalized discharge letter for the patient (PPDL) within 48 hours after admission; and holding a discharge conversation 12 to 24h before discharge. We tested whether the TIP improved timeliness of medical and nursing handovers and investigated the effect of the TIP procedure on length of hospital stay and unplanned readmissions within 30-days post-discharge.

Methods

Study design and setting

We evaluated the implementation of the TIP discharge bundle in an interrupted time series (ITS), which is the strongest design when a randomized controlled trial is not feasible[23 24]. The trial protocol[25] was based on the recommendations for ITS studies,[23] and we adhered to the SQUIRE guidelines for quality improvement reporting[26]. The current study was part of a large national program, initiated by the Dutch Ministry of Health, Welfare, and Sport (abbreviated in Dutch: VWS): 'Addressing Waste in Health Care'. This program was set up in order to reduce inefficiencies in the provision of health care. As part of this program, a TIP study group was established, comprising a study coordinator, two supervisors, one clinical epidemiologist, a policy officer from the Ministry of

For peer review only - http://bmjopen.bmj.com/site/about/guidelines.xhtml

VWS and local project leaders from the eight participating hospitals (one university and seven regional teaching throughout the Netherlands) that implemented the TIP bundle at one of their surgical and one of their internal medicine wards.

Outcomes before and after implementation of the TIP bundle were compared. Therefore, six pre-intervention measurements were conducted before implementation of the TIP and six post-intervention measurements after implementation. During the implementation period of two months no measurements were conducted. February 2016, a kick off meeting was held. Between March 2016 and November 2016, hospitals started with implementation. Data collection started September 2015 and ended June 2017 (Supplement Table 1). All patients (aged ≥18 years) admitted for more than 48h were eligible for inclusion. The Medical Ethics Research Committee (METC) confirmed that the Medical Research Involving Human Subjects Act did not apply to this research project and official approval was not required. Since the study involved a quality improvement intervention with negligible risk of harming patients, individual informed consent was waived for all participating hospitals. This trial was registered with the Dutch Trial Registry number NTR5951.

The discharge process in the Netherlands

In the Netherlands, primary care standards are relatively high and basically every person has a general practitioner (GP). When a person is hospitalized, responsibility is taken over from the GP by the medical specialist. After discharge, patient care becomes the responsibility of the GP again. It is policy for hospitals to provide patient handovers to the GP. However, there are no clear guidelines for hospitals how to arrange their discharge process. The Dutch healthcare inspectorate,[27] indicated that standardized discharge processes are lacking and errors that occur during handovers are often resolved informally.

After discharge from the hospital medical handovers, the hospital physician sends a medical handover to the primary care provider for every patient (e.g., nursing home physician or the GP).

For peer review only - http://bmjopen.bmj.com/site/about/guidelines.xhtml

Medical handovers include information on the reason for admission, diagnosis, comorbidity, the course of admission, medical examinations, treatment, medication, the health status of the patient at discharge, and instructions on follow-up[28]. Nursing handovers are only provided when the patient is discharged to a nursing home or discharged home with post-discharge care at home. Nursing handovers include information on the care provided during hospitalization, current nursing care problems, the reason why (nursing) home care is initiated, and the intended outcomes of the care that will be provided[29].

Intervention

Figure 1 (adapted from van Seben et al.[30]) illustrates how the TIP bundle forms the basis of a safe handover from hospital to primary care for every patient, and if applicable, for patients discharged with post-discharge care (e.g., home care or a nursing home) or for complex patients who require a case manager or transitional care. As described in two previous studies,[25 31] the TIP bundle was developed using input from focus group meetings with professionals, patient surveys and literature. The TIP discharge bundle consists of four elements: 1) planning the discharge date within 48 hours after admission and communication of the discharge date with the patient, 2) to start with arrangements for required post-discharge care within 48 hours after admission; 3) to prepare patient handovers (medical, medication, nurse) and personalized patient discharge letter (PPDL[32]) within 48 hours after admission, 4) to plan a discharge conversation with the patient to explain information from the PPDL 12-24h before discharge. The PPDL is a standardized document, containing understandable information for the patient on the reason for admission, hospital treatment, course of the disease, possible sustained consequences or complications, and information on medication. We constructed checklists based on the TIP, which served as remembering tool for nurses and physicians in the electronic system or on pocket cards.

For peer review only - http://bmjopen.bmj.com/site/about/guidelines.xhtml

Patient and Public Involvement

Our research question was developed from the perspective that patients are discharged with little coordination or follow-up and that they are often unprepared at time of discharge[4 5]. Patients were involved as participants in the construction of the TIP discharge bundle, which was based on, among others, patient satisfaction surveys[25 31]. Further, in a previous study in which the PPDL was developed and implemented, patient satisfaction with the PPDL was also assessed[32].

Protocol adherence

To enhance intervention fidelity and protocol adherence in the different hospitals, regular meetings were held with the TIP study group to report results and provide feedback, to discuss implementation, share experience and learn from each other's practices. A process evaluation was conducted with the project leaders to investigate protocol adherence, implementation strategies and attention paid to implementation. Elements that were considered included leadership and education of project leaders, projects group, extent of implementation of the discharge bundle, and education of physicians and nurses. Feedback points were awarded for all elements and for the extent to which the hospital complied to a certain element, e.g., for every person present at the kick off meeting or for every project meeting that was held. When a hospital partly complied to an element, e.g. automatically generated discharge summaries were provided to the patient instead of a PPDL or feedback on timely handovers was only provided to nurses, 0.5 feedback points were awarded. It was not possible to evaluate percentages of compliance with discharge conversations, planning discharge dates and arrangement of post-discharge care within 48h since these aspects were not reported in patient records. Hospital policies regarding these elements were assessed.

Outcome measures

For peer review only - http://bmjopen.bmj.com/site/about/guidelines.xhtml

Our primary outcome was the number of medical and nursing handovers sent within 24h. This time-frame was based on a report of the Dutch healthcare inspectorate (In Dutch: Inspectie voor de Gezondheidszorg en Jeugd (IGJ)) on the discharge process and handovers, in which it is stated that accurate information needs to be available as quick as possible, but certainly within 24 hours for the next care provider[27]. Medical handovers also include medication handovers and we considered the time that these handovers were sent to the GP. The median time between discharge and the medical handover was considered as secondary outcome. Further, secondary outcomes were length of hospital stay (LOS) and rates of unplanned readmission within 30 days.

Baseline data collection

Data regarding patient characteristics included: demographics, admission ward and medical data (i.e. presence of polypharmacy, comorbidity,[33] number of hospitalization in the six months prior to current hospitalization). Variables were all collected from patient files. All data were reported and analyzed anonymously.

Sample size calculation

Based on the findings of a previous study[31] we expected to find a reduction of 78% in the time between discharge and medical handovers sent. We conducted a power analysis with a number of patients based on the number of hospital beds at the participating wards and feasibility with regards to data collection, which was set at 11 patients. In a simulation study with 16 wards, each contributing 65 patients, we estimated the power to be approximate 91% to demonstrate a reduction of 78% in time until sending the medical handover, assuming that the intraclass correlation coefficient does not exceed 0.05.

Statistical analysis

For peer review only - http://bmjopen.bmj.com/site/about/guidelines.xhtml

Descriptive characteristics of patients were calculated using proportions, means and standard deviations (SD), or medians and interquartile ranges (IQR), as appropriate. Chi-squared analysis and the Mann Whitney test were used to compare pre-intervention and post-intervention patient characteristics. To analyze the intervention-effect, generalized least square analysis were performed to assess changes in level and slope of the regression lines before and after implementation[24]. A change in level is defined as the difference between the observed level at the first post-intervention time point and that predicted by the pre-intervention time trend. A change in trend is defined as the difference between post- and pre-intervention slopes. We explored models with no, a first order autoregressive correlation between consecutive data collection periods, and longer autocorrelation structures. We used the Akaike Information Criterion (AIC) as an estimator of the relative quality of a model and we report the results from the best fitting model. Correction for baseline imbalances as potential confounders led to results with similar estimates and identical interpretation. Based on the extent of protocol adherence and the feedback points awarded, subgroup analyses were performed to assess the intervention effect on the number of medical handovers within 24h and the median time between discharge and medical handovers. Statistical analyses were performed using SPSS Statistics ©, version 24.0, and Rstudio, version 1.0.136 (© 2009 – 2016 Rstudion, Inc).

Results

A total of 2091 patient records (1039 pre- and 1052 post-intervention) were reviewed in order to investigate the effect of the Transfer Intervention Procedure (TIP) on the timeliness of medical and nursing handovers, length of hospital stay (LOS) and unplanned readmission within 30 days. Overall patients had a mean age (SD) of 68.1 (16.6) and 46.4% were male (table 1). There were significant differences between the pre-, and post-intervention group with regard to polypharmacy and the ratio of acute/elective hospitalizations and these variables were considered as potential

For peer review only - http://bmjopen.bmj.com/site/about/guidelines.xhtml

confounders. However, correction for these potential confounders did not provide better models than the presented models.

Protocol adherence

Implementation strategies and protocol adherence are summarized in Supplement Table 1. Based on the process evaluation, three subgroups were identified. Subgroup 1 (hospitals 4 and 8), >30 feedback points, paid considerable attention to implementation and there was relatively high protocol adherence. In subgroup 2 (hospitals 1-3, and 5), 20-30 feedback points, there was relatively high protocol adherence but moderate attention to implementation. In subgroup 3 (hospitals 6 and 7), <10 feedback points, nearly no attention was brought to implementation and there was low compliance.

Medical and nursing handovers

In the total study population, no intervention effect was found on the percentage of medical handovers being sent within 24h after hospital discharge to the GP: 22.7% medical handovers were sent within 24h pre-intervention, 29.1% post-intervention and no significant difference was observed in the levels and slopes between the pre-intervention and post-intervention period. The median (interquartile range, IQR) time between discharge and medical handovers decreased from 6.15 (0.96-15.96) days, pre-intervention to 4.08 (0.33-13.67) days post-intervention. An absolute effect directly after the implementation of the intervention of -0.25 days was found. We observed no significant difference in the levels and slopes. The number of nursing handovers sent within 24h post-discharge was 92.8% pre-intervention and 93.1% post-intervention and no significant difference was observed between levels and slopes. The results are presented in Figure 2 and the parameters estimates are summarized in Table 2.

For peer review only - http://bmjopen.bmj.com/site/about/guidelines.xhtml

Length of hospital stay and unplanned readmission rates

No significant decline in the levels and slopes between the pre-, and post-intervention was found with regard to LOS (β 0.08, 95% CI -0.12 to 0.29 p=0.45) and unplanned readmission rates (β 1.11, 95% CI -2.55 to 0.33 p=0.17). Median (IQR) LOS was 8.17 (4.75-15.13) and 8.56 (4.88-15.91) days and readmissions rates were as high as 11.1% and 12.3% pre-intervention and post-intervention, respectively.

Subgroup analysis

In subgroup 1 (>30 feedback points), an absolute effect of 13.3% more medical handovers sent within 24h post-discharge was observed but this did not result in significant changes in level or slope (Figure 3). A reduction of 5.6 days in the median time between discharge and handovers with a significant change in level directly after the intervention was observed in subgroup 1 (β -5.29, 95% CI -8.70 to 1.87 p=0.02). Pre-intervention, group 2 (20-30 feedback points) had the highest rate of medical handovers sent within 24h and the lowest median time between discharge and medical handovers but no intervention effect was observed. Both pre- and post-intervention, subgroup 3 (<10 points) had the lowest rates of medical handovers sent within 24h, and the highest median time. We observed no intervention effect in subgroup 3.

Discussion

In the total study population, a structured discharge bundle, the Transfer Intervention Procedure (TIP), did not lead to improved timeliness of medical and nursing handovers. Although medical handovers were sent faster post-intervention (pre-intervention median 6.15; post-intervention 4.08 days), we were unable to show significant differences in level and slopes, both with regard to the median time and the number of medical handovers sent within 24h. However, large inter-hospital variation was observed and a significant intervention effect on the median time between discharge

For peer review only - http://bmjopen.bmj.com/site/about/guidelines.xhtml

and medical handovers was seen in those hospitals with relatively high protocol adherence and attention for implementation. Rates of nursing handovers sent within 24h were both pre- and post-intervention above 90%. No intervention effect was found for length of hospital stay (LOS) and readmissions.

Extensive research has been conducted to improve patient handovers from hospital to home[7 16]. Summarizing findings of earlier discharge interventions that aimed to improve coordination of care and communication between hospital and primary care providers, Hesselink et al.,[7] and Kripalani et al.,[8] showed that some studies were able to improve timeliness of medical handovers. These interventions, however, were based on the introduction of fax, email or web-based transfers of information, which is increasingly becoming standard practice in Dutch hospitals. Yet, further improvement may lie in electronic sending systems that support the use of standardized formats that pull information from patient files into (medical) handovers or that send information to the next care provider automatically.

Although a before-after design would probably have led to a significant intervention-effect, the ITS analysis provided valuable information on pre-intervention trends. The observed median time between discharge and sending medical handovers at our first pre-intervention measurement point was consistent with a recent Dutch study[14], but a trend towards sending handovers faster was already observed along the pre-intervention period. During the pre-intervention period, no interventions were implemented and the TIP was introduced and implemented during a two-month implementation period during which no measurements were conducted. However, in the pre-intervention period, attention was already brought to the discharge process, e.g. by establishing project groups and the kick-off meeting. Although these activities were not intended as implementation strategies, in hindsight they might explain why improvements were already observed during the pre-intervention period, particularly since education on the importance of the intervention is an important aspect of implementation[13 34 35].

For peer review only - http://bmjopen.bmj.com/site/about/guidelines.xhtml

Although positive trends in the pre-intervention period were less pronounced in the subgroup analysis, results of the separate analyses support the idea that attention is important. Whereas a significant reduction of six days in the median time between discharge and medical handovers was observed in hospitals that paid much attention to implementation, no intervention effect was observed in hospitals that paid moderate to nearly no attention. It should be noted that the hospitals that paid moderate attention had relatively good pre-intervention scores. A smaller window for improvement in these hospitals might also explain a lack of intervention effect[36].

Implementation of the TIP procedure did not reveal a reduction of LOS. Although a possible explanation can be low overall compliance with our study protocol, it is also probable that over the past years, average LOS has decreased to a minimum[37]. Given current pressure on availability of hospital beds, patients are discharged as soon as possible. This may account for inadequate discharge processes, since physicians are forced to prioritize acute health care over discharge-related tasks[38 39].

Given increasingly shorter LOS[37] and the often complex care needs patient face, patient preparation should be an important aspect of the discharge process. In fact, the most effective discharge interventions seem to have educational components[40]. Unfortunately, given the workload among residents, implementation of a personalized patient discharge letter was unsuccessful. E.g., posing the question “do you feel ready to go home”[41] or post-discharge telephone contact,[7] might be less time-consuming ways to involve patients. However, to prevent readmissions more effort might be necessary. Previous interventions that revealed a reduction in readmission rates, consist of individualized discharge planning or continue post-discharge[16 42]. However, we believe that a structured discharge process such as the TIP should form the basis for a safe handover for every patient (Figure 1).

Implications for further research

For peer review only - http://bmjopen.bmj.com/site/about/guidelines.xhtml

Our study shed light on the difficulties that come along with implementation of quality improvement collaboratives[43]. Given the positive pre-intervention trends and significant reduction in the median time between discharge and medical handovers in hospitals that paid much attention to implementation, further improvements may lie in interventions that create more awareness of the importance of timely handovers and hospital physicians' crucial role in the provision of continuity of care. This might stimulate physicians' intrinsic motivation to provide a structured discharge process and thereby timely handovers[7 39]. Furthermore, we might also want to focus on local factors that lead to insufficient discharge processes. A comprehensive exploration of local barriers for each step in the TIP discharge process might be helpful in order to develop tailor made interventions on a local or department level to improve the discharge process[44].

Limitations

Our study has some limitations. Firstly, we only recorded the date of sending medical handovers. Knowing whether they were received by GPs would also have provided valuable information. Secondly, we did not look at the content of handovers, while this might have given us important insights. Thirdly, medical staff was not blinded for the outcome measure, that is timely discharge letters. Knowing that timeliness of discharge letters was monitored might have altered our results. However, in most hospitals timeliness of discharge letters was already monitored before we started with our research project and the effect is likely to be minimal. Lastly, it was not possible to evaluate percentages of protocol adherence and the process evaluation with the project leaders might have been an overestimation. However, the process evaluation was in line with the efforts observed during implementation.

Conclusion

For peer review only - http://bmjopen.bmj.com/site/about/guidelines.xhtml

For peer review only

Implementation of a structured discharge bundle, the Transfer Intervention Procedure (TIP), did not lead to more medical and nursing handovers sent within 24h post-discharge. Large inter-hospital variation was observed however, and a significant intervention effect on the median time between discharge and medical handovers was seen in those hospitals with high protocol adherence and that brought much attention to implementation. We believe that future interventions should continue to create awareness of the importance of timely handovers and we hope that our study contributes to this, stimulating hospitals to further structure and improve their discharge process.

Contributors: BB, SG, and RVS designed the study. BB and SG, conceived the study and obtained funding. RVS collected data and JM and RVS performed statistical analysis. All authors contributed to drafting the manuscript or revised it critically and gave final approval for publication. The authors would like to acknowledge the contribution of the TIP study group, consisting in addition to the authors, of the following members Hanneke Pullens (Catharina Hospital Eindhoven), Barbara van Munster (Gelre Hospitals Apeldoorn), Annemarie van der Lugt (Haven Hospital Rotterdam), Alie Haze-Visser (Lange Land Hospital Zoetermeer), Agnes van 't Hof (Maxima Medical Center Veldhoven), Mariët Dirkzwager (OLVG Amsterdam), Stella de Regt (Reinier de Graaf Hospital Delft), Suzan Vroomen (Academic Medical Center Amsterdam) and Lisette Bruns (Dutch Ministry of Health, Welfare, and Sport). Furthermore, we thank Rachel de Vries en Vera van Miltenburg for their assistance with data collection.

Funding sources: This work was supported by the Dutch Ministry of Health, Welfare and Sport [grant number: 324798].

Conflicts of interest: None declared.

Data sharing statement: No additional data are available.

For peer review only - http://bmjopen.bmj.com/site/about/guidelines.xhtml

References

1. Eurostat Statistics Explained. Hospital discharges and length of stay statistics 2016 [Available from: http://ec.europa.eu/eurostat/statistics-explained/index.php/Hospital_discharges_and_length_of_stay_statistics accessed October 17 2017.
2. Jean Hall M, DeFrances CJ, Williams SN, Golonsinskiy MS, Schwartzman A. National Health Statistics Reports. *US Department of Health and Guman Services Centers for Disease Control and Prevention National Center for Health Statistics* 2010;29
3. Boyd CM, Darer J, Boult C, Fried LP, Boult L, Wu AW. Clinical practice guidelines and quality of care for older patients with multiple comorbid diseases: implications for pay for performance. *Jama* 2005;294(6):716-24. doi: 10.1001/jama.294.6.716 [published Online First: 2005/08/11]
4. Henderson A, Zernike W. A study of the impact of discharge information for surgical patients. *Journal of advanced nursing* 2001;35(3):435-41. [published Online First: 2001/08/08]
5. Holland DE, Mistiaen P, Bowles KH. Problems and unmet needs of patients discharged "home to self-care". *Professional case management* 2011;16(5):240-50; quiz 51-2. doi: 10.1097/NCM.0b013e31822361d8 [published Online First: 2011/08/19]
6. Greenwald JL, Jack BW. Preventing the preventable: reducing rehospitalizations through coordinated, patient-centered discharge processes. *Professional case management* 2009;14(3):135-40; quiz 41-2. doi: 10.1097/NCM.0b013e318198d4e1 [published Online First: 2009/05/29]
7. Hesselink G, Schoonhoven L, Barach P, et al. Improving patient handovers from hospital to primary care: a systematic review. *Annals of internal medicine* 2012;157(6):417-28. doi: 10.7326/0003-4819-157-6-201209180-00006 [published Online First: 2012/09/19]
8. Kripalani S, LeFevre F, Phillips CO, Williams MV, Basaviah P, Baker DW. Deficits in communication and information transfer between hospital-based and primary care physicians: implications for patient safety and continuity of care. *Jama* 2007;297(8):831-41. doi: 10.1001/jama.297.8.831 [published Online First: 2007/03/01]
9. Forster AJ, Murff HJ, Peterson JF, Gandhi TK, Bates DW. The incidence and severity of adverse events affecting patients after discharge from the hospital. *Annals of internal medicine* 2003;138(3):161-7. [published Online First: 2003/02/01]
10. Moore C, Wisnivesky J, Williams S, McGinn T. Medical errors related to discontinuity of care from an inpatient to an outpatient setting. *Journal of general internal medicine* 2003;18(8):646-51. [published Online First: 2003/08/13]
11. Fluitman KS, van Galen LS, Merten H, et al. Exploring the preventable causes of unplanned readmissions using root cause analysis: Coordination of care is the weakest link. *European journal of internal medicine* 2016;30:18-24. doi: 10.1016/j.ejim.2015.12.021 [published Online First: 2016/01/18]
12. Kansagara D, Englander H, Salanitro A, et al. Risk prediction models for hospital readmission: a systematic review. *Jama* 2011;306(15):1688-98. doi: 10.1001/jama.2011.1515 [published Online First: 2011/10/20]
13. Merten H, van Galen LS, Wagner C. Safe handover. *BMJ (Clinical research ed)* 2017;359:j4328. doi: 10.1136/bmj.j4328 [published Online First: 2017/10/11]
14. Langelaan M, Baines RJ, de Bruijne MC, Wagner C. Association of admission and patient characteristics with quality of discharge letters: posthoc analysis of a retrospective study. *BMC health services research* 2017;17(1):225. doi: 10.1186/s12913-017-2149-8 [published Online First: 2017/03/23]
15. Dharmarajan K, Hsieh AF, Lin Z, et al. Diagnoses and timing of 30-day readmissions after hospitalization for heart failure, acute myocardial infarction, or pneumonia. *Jama* 2013;309(4):355-63. doi: 10.1001/jama.2012.216476 [published Online First: 2013/01/24]

For peer review only - http://bmjopen.bmj.com/site/about/guidelines.xhtml

16. Verhaegh KJ, MacNeil-Vroomen JL, Eslami S, Geerlings SE, de Rooij SE, Buurman BM. Transitional care interventions prevent hospital readmissions for adults with chronic illnesses. *Health affairs (Project Hope)* 2014;33(9):1531-9. doi: 10.1377/hlthaff.2014.0160 [published Online First: 2014/09/10]
17. Prvu Bettger J, Alexander KP, Dolor RJ, et al. Transitional care after hospitalization for acute stroke or myocardial infarction: a systematic review. *Annals of internal medicine* 2012;157(6):407-16. doi: 10.7326/0003-4819-157-6-201209180-00004 [published Online First: 2012/09/19]
18. Shepperd S, Lannin NA, Clemson LM, McCluskey A, Cameron ID, Barras SL. Discharge planning from hospital to home. *The Cochrane database of systematic reviews* 2013;1:Cd000313. doi: 10.1002/14651858.CD000313.pub4 [published Online First: 2013/02/27]
19. Buurman BM, Parlevliet JL, Allore HG, et al. Comprehensive Geriatric Assessment and Transitional Care in Acutely Hospitalized Patients: The Transitional Care Bridge Randomized Clinical Trial. *JAMA internal medicine* 2016;176(3):302-9. doi: 10.1001/jamainternmed.2015.8042 [published Online First: 2016/02/18]
20. Naylor MD, Brooten DA, Campbell RL, Maislin G, McCauley KM, Schwartz JS. Transitional care of older adults hospitalized with heart failure: a randomized, controlled trial. *Journal of the American Geriatrics Society* 2004;52(5):675-84. doi: 10.1111/j.1532-5415.2004.52202.x [published Online First: 2004/04/17]
21. The Joint Commission. Hot Topics in Health Care. Transitions of Care: The need for a more effective approach to continuing patient care. *The Joint Commission* 2012
22. Kripalani S, Jackson AT, Schnipper JL, Coleman EA. Promoting effective transitions of care at hospital discharge: a review of key issues for hospitalists. *Journal of hospital medicine* 2007;2(5):314-23. doi: 10.1002/jhm.228 [published Online First: 2007/10/16]
23. Jandoc R, Burden AM, Mamdani M, Levesque LE, Cadarette SM. Interrupted time series analysis in drug utilization research is increasing: systematic review and recommendations. *Journal of clinical epidemiology* 2015;68(8):950-6. doi: 10.1016/j.jclinepi.2014.12.018 [published Online First: 2015/04/22]
24. Penfold RB, Zhang F. Use of interrupted time series analysis in evaluating health care quality improvements. *Academic pediatrics* 2013;13(6 Suppl):S38-44. doi: 10.1016/j.acap.2013.08.002 [published Online First: 2013/12/07]
25. van Seben R, Geerlings SE, Verhaegh KJ, Hilders CG, Buurman BM. Implementation of a Transfer Intervention Procedure (TIP) to improve handovers from hospital to home: interrupted time series analysis. *BMC health services research* 2016;16:479. doi: 10.1186/s12913-016-1730-x [published Online First: 2016/09/09]
26. Davidoff F, Batalden P, Stevens D, Ogrinc G, Mooney SE. Publication guidelines for quality improvement studies in health care: evolution of the SQUIRE project. *BMJ (Clinical research ed)* 2009;338:a3152. doi: 10.1136/bmj.a3152 [published Online First: 2009/01/21]
27. Dutch Health Care Inspectorate (IGZ). Continuïteit van zorg voor kwetsbare ouderen vanuit het ziekenhuis naar verpleeg- en verzorgingshuizen, thuiszorg en huisartsen niet gewaarborgd. . 2015
28. Joint Commission on Accreditation of Healthcare Organizations. Accreditation Standards for Hospitals. Standard ACC.3 Oakbrook Terrace, IL: The Joint Commission 2010.
29. V&VN. Richtlijn Verpleegkundige en verzorgende verslaglegging September 2011 [
30. van Seben R, Geerlings SE, Buurman BM. [10 tips for improving patient handovers from hospital to other health care settings]. *Ned Tijdschr Geneeskd* 2017;161(0):D1031. [published Online First: 2017/04/27]
31. Verhaegh KJ, Buurman BM, Veenboer GC, de Rooij SE, Geerlings SE. The implementation of a comprehensive discharge bundle to improve the discharge process: a quasi-experimental

For peer review only - http://bmjopen.bmj.com/site/about/guidelines.xhtml

study. *The Netherlands journal of medicine* 2014;72(6):318-25. [published Online First: 2014/10/17]
32. Buurman BM, Verhaegh KJ, Smeulers M, et al. Improving handoff communication from hospital to home: the development, implementation and evaluation of a personalized patient discharge letter. *International journal for quality in health care : journal of the International Society for Quality in Health Care / ISQua* 2016;28(3):384-90. doi: 10.1093/intqhc/mzw046 [published Online First: 2016/05/27]
33. Charlson ME, Pompei P, Ales KL, MacKenzie CR. A new method of classifying prognostic comorbidity in longitudinal studies: development and validation. *Journal of chronic diseases* 1987;40(5):373-83. [published Online First: 1987/01/01]
34. Michie S, van Stralen MM, West R. The behaviour change wheel: a new method for characterising and designing behaviour change interventions. *Implementation science : IS* 2011;6:42. doi: 10.1186/1748-5908-6-42 [published Online First: 2011/04/26]
35. Spoorenberg V, Geerlings SE, Geskus RB, de Reijke TM, Prins JM, Hulscher ME. Appropriate antibiotic use for patients with complicated urinary tract infections in 38 Dutch Hospital Departments: a retrospective study of variation and determinants. *BMC infectious diseases* 2015;15:505. doi: 10.1186/s12879-015-1257-5 [published Online First: 2015/11/11]
36. Ivers N, Jamtvedt G, Flottorp S, et al. Audit and feedback: effects on professional practice and healthcare outcomes. *The Cochrane database of systematic reviews* 2012(6):Cd000259. doi: 10.1002/14651858.CD000259.pub3 [published Online First: 2012/06/15]
37. Coppa Consultancy. Ligduurmonitor Nederlandse ziekenhuizen 2013 & 2014. Trendbreuk in de ligduur. 2015 [Available from: http://www.coppa.nl/wp-content/uploads/2016/01/Ligduurmonitor-2013-2014.pdf accessed November, 2017.
38. Greysen SR, Schiliro D, Horwitz LI, Curry L, Bradley EH. "Out of sight, out of mind": housestaff perceptions of quality-limiting factors in discharge care at teaching hospitals. *Journal of hospital medicine* 2012;7(5):376-81. doi: 10.1002/jhm.1928 [published Online First: 2012/03/02]
39. Hesselink G, Vernooij-Dassen M, Pijnenborg L, et al. Organizational culture: an important context for addressing and improving hospital to community patient discharge. *Medical care* 2013;51(1):90-8. doi: 10.1097/MLR.0b013e31827632ec [published Online First: 2012/11/08]
40. Mistiaen P, Francke AL, Poot E. Interventions aimed at reducing problems in adult patients discharged from hospital to home: a systematic meta-review. *BMC health services research* 2007;7:47. doi: 10.1186/1472-6963-7-47 [published Online First: 2007/04/06]
41. van Galen LS, Brabrand M, Cooksley T, et al. Patients' and providers' perceptions of the preventability of hospital readmission: a prospective, observational study in four European countries. *BMJ quality & safety* 2017 doi: 10.1136/bmjqs-2017-006645 [published Online First: 2017/06/24]
42. Leppin AL, Gionfriddo MR, Kessler M, et al. Preventing 30-day hospital readmissions: a systematic review and meta-analysis of randomized trials. *JAMA internal medicine* 2014;174(7):1095-107. doi: 10.1001/jamainternmed.2014.1608 [published Online First: 2014/05/14]
43. Hulscher ME, Schouten LM, Grol RP, Buchan H. Determinants of success of quality improvement collaboratives: what does the literature show? *BMJ quality & safety* 2013;22(1):19-31. doi: 10.1136/bmjqs-2011-000651 [published Online First: 2012/08/11]
44. Flottorp SA, Oxman AD, Krause J, et al. A checklist for identifying determinants of practice: a systematic review and synthesis of frameworks and taxonomies of factors that prevent or enable improvements in healthcare professional practice. *Implementation science : IS* 2013;8:35. doi: 10.1186/1748-5908-8-35 [published Online First: 2013/03/26]

For peer review only - http://bmjopen.bmj.com/site/about/guidelines.xhtml

Table 1. Baseline characteristics

Variable	Overall (N=2091)	Pre-intervention (N=1039)	Post-intervention (N=1052)
**Age in years,** mean (SD)[a]	68.07 (16.57)	67.66 (16.70)	68.48 (16.45)
**Male,** No. (%)	971 (46.4)	493 (47.4)	478 (45.4)
**Living arrangements before admission,** No.(%)			
Independent	1814 (86.7)	883 (84.9)	931 (88.5)
Nursing home	49 (2.3)	27 (2.6)	22 (2.1)
Senior residence/Assisted living	168 (8.1)	91 (8.8)	77 (7.3)
Missing	60 (2.9)	38 (3.7)	22 (2.1)
**Marital status,** No. **(%)**			
Married or living together	1125 (53.8)	556 (53.5)	569 (54.1)
Single or divorced	456 (21.8)	212 (20.4)	244 (23.2)
Widow/widower	435 (20.8)	224 (21.6)	211 (20.1)
Missing	75 (3.6)	47 (4.5)	28 (2.7)
**Charlson Comorbidity Index**[b] (mean, SD[a])	2.05 (2.05)	2.10 (2.08)	2.01 (2.03)
**Polypharmacy,** No. (%)[c, d, e]	1247 (59.6)	586 (56.4)	661 (62.8)
Missing	12 (.6)	8 (.8)	4 (.4)
**Hospitalization in past 6 months,** No. (%)	705 (33.7)	339 (32.6)	336 (34.8)
**Acute hospitalization,** No. (%)[c, f]	73.0 (73.0)	725 (69.8)	801 (76.1)
**Admission ward, internal medicine** No. (%)	1051 (50.3)	524 (50.4)	527 (50.1)
**Discharge destination,** No. (%)			
Home	1551 (74.2)	770 (74.1)	781 (74.2)
Other health care setting, of which	482 (23.1)	238 (23.0)	244 (23.2)
Rehabilitation center	268 (12.8)	120 (11.5)	148 (14.1)
Nursing home	158 (7.6)	80 (7.7)	78 (7.4)
Assisted living	34 (1.6)	26 (2.5)	8 (0.8)
Other hospital	22 (1.1)	12 (1.2)	10 (1.0)
Missing	58 (2.8)	31 (3.0)	27 (2.6)

[a] Standard Deviation, [b] Range of 0 to 31, with a higher score indicating more or more severe comorbidity[33], [c] Use of 5 or more different medications, [d] Chi-Square, [e] P-value = 0.004, [f] P-value = 0.001

For peer review only - http://bmjopen.bmj.com/site/about/guidelines.xhtml

Table 2. Interrupted time series analysis; medical and nursing handovers

	Medical handovers <24 hrs after discharge (%) [a]			***Time between discharge and medical letter (days)*** [b]			***Nursing handovers <24 hrs after discharge (%)*** [a]		
	**β (SE)**	**95% CI**	**p-value**	**β (SE)**	**95% CI**	**p-value**	**β (SE)**	**95% CI**	**p-value**
Intercept	17.51 (3.79)	10.08 to 24.93	<0.01	7.20 (0.29)	6.63 to 7.76	<0.01	91.85 (2.71)	86.53 to 97.16	<0.01
Trend pre-intervention (β1)	1.49 (0.97)	-0.42 to 3.40	0.16	-0.30 (0.07)	-0.45 to -0.16	<0.01	0.28 (0.70)	-1.09 to 1.64	0.70
Level change directly after intervention (β2)	6.43 (10.13)	-13.43 to 26.28	0.54	-0.62 (0.74)	-2.07 to 0.84	0.43	6.32 (7.25)	-7.89 to 20.53	0.41
Trend differences (β3)	-0.94 (1.38)	-3.64 to 1.75	0.51	0.05 (0.10)	-0.14 to 0.25	0.61	-0.81 (0.99)	-2.74 to 1.12	0.43
	Absolute effect directly after intervention: -0.17%			Absolute effect directly after intervention: -0.25 days			Absolute effect directly after intervention: 0.62%		

β1 estimates the pre-intervention slope.
β2 estimates the difference between the observed level just after the intervention started and that predicted by the pre-intervention slope.
β3 estimates the difference in trend/slopes between the pre-intervention and post-intervention period.
SE: Standard Error, CI: Confidence Interval

For peer review only - http://bmjopen.bmj.com/site/about/guidelines.xhtml

Figure 1. Pyramid for post-discharge care

A structured discharge process such as the TIP procedure should form the basis for every patient. For patients discharged with post-discharge care (20-25%), nursing handovers should be set up within 48h after admission and be sent within 24h post-discharge. Complex patients with a high readmission risk (10%) require a (nurse) case manager or transitional care in the transition from hospital to home.

Figure 2.

Panel A The number of medical handovers sent within 24 hours.

Panel B median time in days between discharge and the medical handovers.

Figure 3. Hospital differences based on implementation score.

The inter-hospital differences in rates of medical handovers being sent within 24h in the pre- and post-intervention based on the extent of implementation and used implementation strategies. Group 1 received >30 feedback implementation points, group 2 received 20-30 implementation points, group 3 received <20 points.

For peer review only

For peer review only - http://bmjopen.bmj.com/site/about/guidelines.xhtml

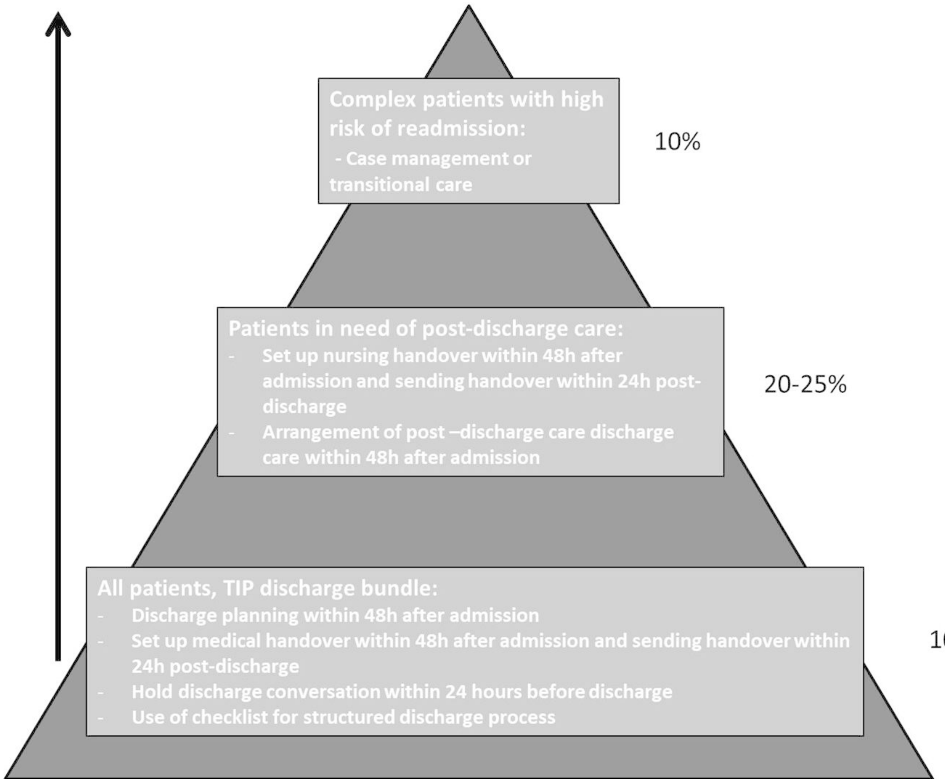

Figure 1. Pyramid for post-discharge care
A structured discharge process such as the TIP procedure should form the basis for every patient. For patients discharged with post-discharge care (20-25%), nursing handovers should be set up within 48h after admission and be sent within 24h post-discharge. Complex patients with a high readmission risk (10%) require a (nurse) case manager or transitional care in the transition from hospital to home. Adapted from van Seben et al.40

116x92mm (300 x 300 DPI)

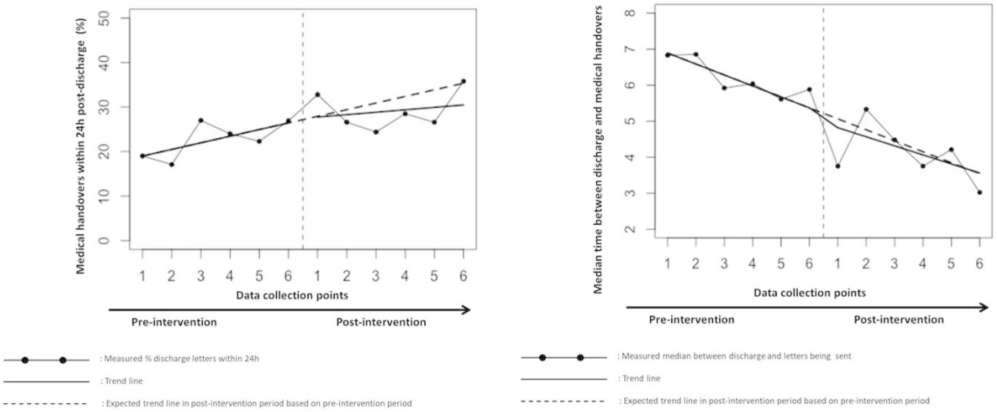

Figure 2.
Panel A The number of medical handovers sent within 24 hours.
Panel B median time in days between discharge and the medical handovers.

113x50mm (300 x 300 DPI)

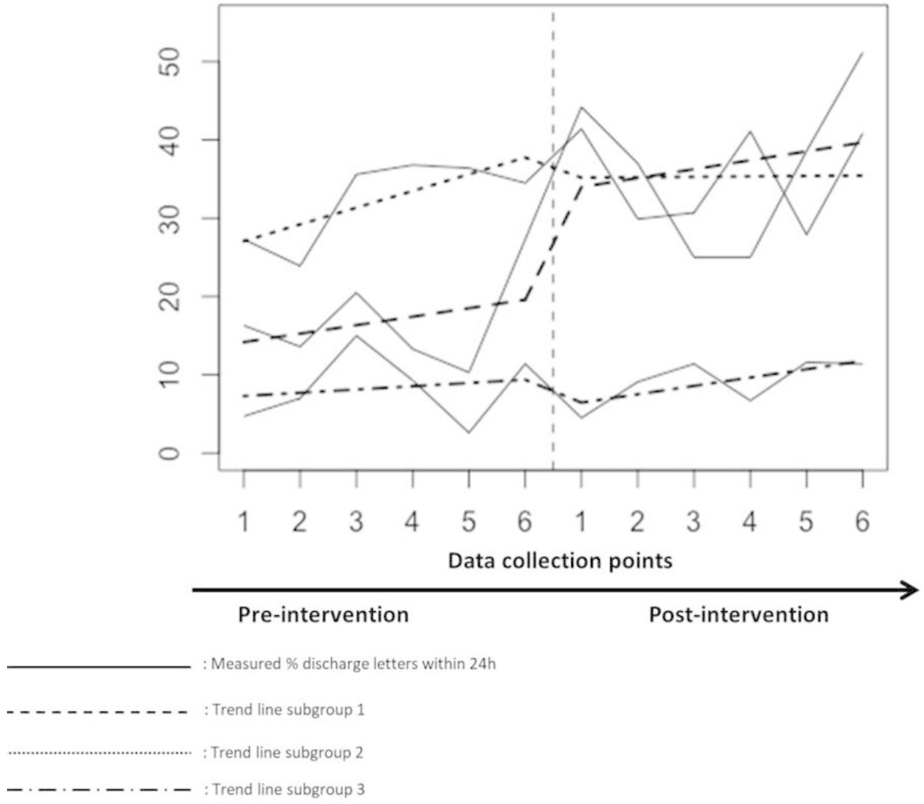

Figure 3. Hospital differences based on implementation score.
The inter-hospital differences in rates of medical discharge letters being sent within 24h in the pre- and post-intervention based on the extent of implementation and used implementation strategies. Group 1 received >30 feedback implementation points, group 2 received 20-30 implementation points, group 3 received <20 points.

82x69mm (300 x 300 DPI)

Supplement Table 1. Adherence to the Intervention Protocol

	Hospital 1	Hospital 2	Hospital 3	Hospital 4	Hospital 5	Hospital 6	Hospital 7	Hospital 8
Pre-intervention<br>Implementation<br>Post-intervention	Sep '15 - Feb '16<br>March '16 - April '16<br>May '16 – Oct '16	Oct '15 - March '16<br>April '16 - May '16<br>June '16 - Nov '16	Jan '16 - June '16<br>July '16 - Aug '16<br>Sep '16 – Feb '17	Dec '15 - May '16<br>June '16 - July '16<br>Aug '16 - Jan '16	March '16 – Aug '16<br>Sep '16 - Oct '16<br>Nov '16 – April '17	April '16 – Sep '16<br>Oct '16 - Nov '16<br>Dec '16 – May '17	May '16 – Oct '16<br>Nov '16 - Dec '16<br>Jan '17 – June '17	April '16 – Sep '16<br>Oct '16 - Nov '16<br>Dec '16 – May '17
**Leadership and education of project leaders**								
Who were present at the kick off meeting February, 2016?	Hospital president; local project leader; 2 team leaders (nurses); 2 physicians; nurse; pharmacists	Local project leader; geriatrician; head of the liaison department; physician	2 local project leaders; head of the liaison department	Chief of staff; local project leader; team leader surgery ward (nurse); head of the liaison department	Local project leader; head of the liaison department; manager patient logistics; 2 team leaders (nurses)	2 local project leaders	Local project leader	Local project leader; senior researcher transitional care; medical specialist,
Who were present at the first feedback session?	Project leader; head of the liaison department	Project leader; pharmacist; communication assistant	2 local project leaders; liaison nurse	Project leader; liaison nurse; nurse geriatrics	Local project leader	2 local project leaders	-	2 project leaders
Who were present at the second feedback session?	Project leader	Project leader	2 project leaders	-	Project leader	-	Project leader	2 project leaders
Implementation points	*10*	*8*	*8*	*7*	*7*	*4*	*2*	*7*
**Project group**								
Was there a local TIP project group, and who participated?	Yes, project leader; 2 senior nurses of participating wards, management assistant	Yes, project leader; geriatrician; head liaison department, physician; pharmacist; communication assistant; manager Security & Services	Yes, 2 project leaders; 2 residents; 2 medical specialists; nurse; liaison nurse; pharmacist; manager	Yes, chief of staff; project leader; 2 team leaders (nurses); head liaison department; orthopedist	Yes, project leader; head liaison department; 2 medical specialists, geriatrician	No	No	Yes, local project leader; 3 medical specialists; 2 residents, manager quality and safety; manager process optimization; medical director
How often did the local project group meet?<br>1 point per meeting	Monthly for 2 months, during pilot period every week (2 months).	Every five weeks during pre-intervention and pilot period	2 times, before pilot period.	Every two weeks during pre-intervention period	Every six weeks, during pre-intervention and pilot period	-	-	Monthly during pre-intervention, pilot period and first two months of post-

For peer review only

								intervention period
Implementation points	*10*	*12*	*10*	*18*	*10*	*0*	*0*	*17*
**Implementation of TIP elements**								
Was it policy to set a discharge date within 48h after admission?	Yes	Yes	Yes	Yes	Yes	Yes	Yes	Yes
Was the planned discharge date communicated to patients?	Yes	Yes	Yes	Yes	Yes	No	Yes	Yes
Was it policy to start with arrangement of required post-discharge care within 48h after admission?	No, liaison nurse has to wait for final discharge date	No, liaison nurse has to wait for final discharge date	Yes	Yes	No, liaison department is overloaded	Yes	No, liaison nurse has to wait for final discharge date	Yes
Was it policy to set up patient handovers within 48h after admission?	No	No	No	No	No	No	No	No
Did physicians hold discharge conversations, using a checklist during the pilot period?	No	No	No	No	No	No	No	No
Does the nurse holds a discharge conversation, using a checklist?	Yes	No	Yes	Yes	Yes	No	Yes	Yes
Was the patient discharge letter implemented?	Yes, but only for some diagnosis at internal medicine ward.	No	No	No	No	Yes , but only at internal medicine ward for frail older patients.	No	No, a discharge summary was implemented instead.
Implementation points	*3.5*	*2*	*4*	*4*	*3*	*2.5*	*3*	*4.5*
**Education of physicians and nurses**								

For peer review only

How were physicians and nurses informed about the TIP and how often?		Kick-off meeting at participating wards; during morning report; working instructions were sent by email to all physicians		Kick-off meeting; meeting at participating wards; E-learning; 1 feedback meeting		During morning reports; project leader informed every physician separately; intranet; email		During morning reports; intranet; email; posters & pocket cards; and project leaders went to participating wards to inform physicians and nurses		Email and project leader went to participating wards		Kick-off meeting, during several morning reports		Physicians were not educated with regard to the intervention		During several morning report; email; project leaders went to participating wards to inform physicians and nurses; medical specialists from project group informed physicians in person	
Did physicians and/or nurses receive feedback with regard to their discharge letters and if yes, how often?		No		No		No		No		No		Only for nurses		No		Yes, daily on internal medicine and monthly on surgery ward, via email.	
Implementation points		*3*		*4*		*4*		*5*		*2*		*2.5*		*0*		*5*	
**Total implementation points**		**26.5**		**26**		**26**		**34**		**22**		**9**		**5**		**33.5**	
**Pre-intervention vs. post-intervention period scores**																	
**Pre-intervention period**																	
median	% letters within 24h	8.15	9.0	0.90	47.3	6.71	23.5	10.48	13.1	0.79	50.0	6.79	9.2	14.21	7.6	5.83	20.9
**Post-intervention**																	
median	% letters within 24h	9.08	19.5	1.0	48.5	5.48	24.2	5.79	19.7	0.29	49.6	7.98	16.7	22.44	1.5	0.83	53.8

For peer review only

Revised Standards for Quality Improvement Reporting Excellence (SQUIRE 2.0)
September 15, 2015

Text Section and Item Name	Section or Item Description
**Notes to authors**	• The SQUIRE guidelines provide a framework for reporting new knowledge about how to improve healthcare<br>• The SQUIRE guidelines are intended for reports that describe system level work to improve the quality, safety, and value of healthcare, and used methods to establish that observed outcomes were due to the intervention(s).<br>• A range of approaches exists for improving healthcare. SQUIRE may be adapted for reporting any of these.<br>• Authors should consider every SQUIRE item, but it may be inappropriate or unnecessary to include every SQUIRE element in a particular manuscript.<br>• The SQUIRE Glossary contains definitions of many of the key words in SQUIRE.<br>• The Explanation and Elaboration document provides specific examples of well-written SQUIRE items, and an in-depth explanation of each item.<br>• Please cite SQUIRE when it is used to write a manuscript.
**Title and Abstract**	
**1. Title**	Indicate that the manuscript concerns an initiative to improve healthcare (broadly defined to include the quality, safety, effectiveness, patient-centeredness, timeliness, cost, efficiency, and equity of healthcare)
**2. Abstract**	a. Provide adequate information to aid in searching and indexing<br>b. Summarize all key information from various sections of the text using the abstract format of the intended publication or a structured summary such as: background, local problem, methods, interventions, results, conclusions
**Introduction**	*Why did you start?*
**3. Problem Description**	Nature and significance of the local problem
**4. Available knowledge**	Summary of what is currently known about the problem, including relevant previous studies

5. Rationale	Informal or formal frameworks, models, concepts, and/or theories used to explain the problem, any reasons or assumptions that were used to develop the intervention(s), and reasons why the intervention(s) was expected to work
6. Specific aims	Purpose of the project and of this report
**Methods**	*What did you do?*
7. Context	Contextual elements considered important at the outset of introducing the intervention(s)
8. Intervention(s)	a. Description of the intervention(s) in sufficient detail that others could reproduce it<br>b. Specifics of the team involved in the work
9. Study of the Intervention(s)	a. Approach chosen for assessing the impact of the intervention(s)<br>b. Approach used to establish whether the observed outcomes were due to the intervention(s)
10. Measures	a. Measures chosen for studying processes and outcomes of the intervention(s), including rationale for choosing them, their operational definitions, and their validity and reliability<br>b. Description of the approach to the ongoing assessment of contextual elements that contributed to the success, failure, efficiency, and cost<br>c. Methods employed for assessing completeness and accuracy of data
11. Analysis	a. Qualitative and quantitative methods used to draw inferences from the data<br>b. Methods for understanding variation within the data, including the effects of time as a variable
12. Ethical Considerations	Ethical aspects of implementing and studying the intervention(s) and how they were addressed, including, but not limited to, formal ethics review and potential conflict(s) of interest
**Results**	*What did you find?*
13. Results	a. Initial steps of the intervention(s) and their evolution over time (*e.g.*, time-line diagram, flow chart, or table), including modifications made to the intervention during the project<br>b. Details of the process measures and outcome<br>c. Contextual elements that interacted with the intervention(s)<br>d. Observed associations between outcomes, interventions, and relevant contextual elements<br>e. Unintended consequences such as unexpected benefits, problems, failures, or costs associated with the intervention(s).<br>f. Details about missing data
**Discussion**	*What does it mean?*
14. Summary	a. Key findings, including relevance to the rationale and specific aims<br>b. Particular strengths of the project

For peer review only

**15. Interpretation**	a. Nature of the association between the intervention(s) and the outcomes<br>b. Comparison of results with findings from other publications<br>c. Impact of the project on people and systems<br>d. Reasons for any differences between observed and anticipated outcomes, including the influence of context<br>e. Costs and strategic trade-offs, including opportunity costs
**16. Limitations**	a. Limits to the generalizability of the work<br>b. Factors that might have limited internal validity such as confounding, bias, or imprecision in the design, methods, measurement, or analysis<br>c. Efforts made to minimize and adjust for limitations
**17. Conclusions**	a. Usefulness of the work<br>b. Sustainability<br>c. Potential for spread to other contexts<br>d. Implications for practice and for further study in the field<br>e. Suggested next steps
**Other information**	
**18. Funding**	Sources of funding that supported this work. Role, if any, of the funding organization in the design, implementation, interpretation, and reporting

For peer review only

Table 2. Glossary of key terms used in SQUIRE 2.0. This Glossary provides the intended meaning of selected words and phrases as they are used in the SQUIRE 2.0 Guidelines. They may, and often do, have different meanings in other disciplines, situations, and settings.

Assumptions
Reasons for choosing the activities and tools used to bring about changes in healthcare services at the system level.

Context
Physical and sociocultural makeup of the local environment (for example, external environmental factors, organizational dynamics, collaboration, resources, leadership, and the like), and the interpretation of these factors ("sense-making") by the healthcare delivery professionals, patients, and caregivers that can affect the effectiveness and generalizability of intervention(s).

Ethical aspects
The value of system-level initiatives relative to their potential for harm, burden, and cost to the stakeholders. Potential harms particularly associated with efforts to improve the quality, safety, and value of healthcare services include opportunity costs, invasion of privacy, and staff distress resulting from disclosure of poor performance.

Generalizability
The likelihood that the intervention(s) in a particular report would produce similar results in other settings, situations, or environments (also referred to as external validity).

Healthcare improvement
Any systematic effort intended to raise the quality, safety, and value of healthcare services, usually done at the system level. We encourage the use of this phrase rather than "quality improvement," which often refers to more narrowly defined approaches.

Inferences
The meaning of findings or data, as interpreted by the stakeholders in healthcare services – improvers, healthcare delivery professionals, and/or patients and families

Initiative
A broad term that can refer to organization-wide programs, narrowly focused projects, or the details of specific interventions (for example, planning, execution, and assessment)

Internal validity
Demonstrable, credible evidence for efficacy (meaningful impact or change) resulting from introduction of a specific intervention into a particular healthcare system.

Intervention(s)
The specific activities and tools introduced into a healthcare system with the aim of changing its performance for the better. Complete description of an intervention includes its inputs, internal activities, and outputs (in the form of a logic model, for example), and the mechanism(s) by which these components are expected to produce changes in a system's performance.

Opportunity costs

For peer review only

Loss of the ability to perform other tasks or meet other responsibilities resulting from the diversion of resources needed to introduce, test, or sustain a particular improvement initiative

Problem
Meaningful disruption, failure, inadequacy, distress, confusion or other dysfunction in a healthcare service delivery system that adversely affects patients, staff, or the system as a whole, or that prevents care from reaching its full potential

Process
The routines and other activities through which healthcare services are delivered

Rationale
Explanation of why particular intervention(s) were chosen and why it was expected to work, be sustainable, and be replicable elsewhere.

Systems
The interrelated structures, people, processes, and activities that together create healthcare services for and with individual patients and populations. For example, systems exist from the personal self-care system of a patient, to the individual provider-patient dyad system, to the microsystem, to the macrosystem, and all the way to the market/social/insurance system. These levels are nested within each other.

Theory or theories
Any "reason-giving" account that asserts causal relationships between variables (causal theory) or that makes sense of an otherwise obscure process or situation (explanatory theory). Theories come in many forms, and serve different purposes in the phases of improvement work. It is important to be explicit and well-founded about any informal and formal theory (or theories) that are used.

For peer review only

BMJ Open

A safe handover for every patient: an interrupted time series analysis to test the effect of a structured discharge bundle

Journal:	*BMJ Open*
Manuscript ID	bmjopen-2018-023446.R2
Article Type:	Research
Date Submitted by the Author:	13-Feb-2019
Complete List of Authors:	van Seben, Rosanne; AMC, Internal Medicine, section of Geriatric Medicine<br>Geerlings, Suzanne; AMC, Internal Medicine, Division of Infectious Diseases<br>Maaskant, Jolanda; AMC, Emma Children's Hospital<br>Buurman, Bianca; Academic Medical Center, Internal Medicine, section of Geriatric Medicine
**Primary Subject Heading**:	Health services research
Secondary Subject Heading:	Communication, Health services research
Keywords:	Discharge Bundle, Patient Handovers, Discharge Letter, Patient Safety, Interrupted Time Series, Quality Improvement

SCHOLARONE™
Manuscripts

A safe handover for every patient: an interrupted time series analysis to test the effect of a structured discharge bundle

Rosanne van Seben (corresponding author)

Amsterdam UMC, University of Amsterdam, Department of Internal Medicine, Section of Geriatric Medicine, Amsterdam Public Health research institute, Meibergdreef 9, Amsterdam, The Netherlands

r.vanseben@amc.uva.nl, +312 56 61647

Suzanne E. Geerlings

Amsterdam UMC, University of Amsterdam, Department of Internal Medicine, Section of Infectious Diseases, Meibergdreef 9, Amsterdam, The Netherlands

Jolanda M. Maaskant

Amsterdam UMC, University of Amsterdam, Emma Children's Hospital, Meibergdreef 9, Amsterdam, The Netherlands

Bianca M. Buurman

Amsterdam UMC, University of Amsterdam, Department of Internal Medicine, Section of Geriatric Medicine, Amsterdam Public Health research institute, Meibergdreef 9, Amsterdam, The Netherlands & ACHIEVE Centre of Expertise, Faculty of Health, Amsterdam University of Applied Sciences, Amsterdam, the Netherlands

On behalf of the TIP study group: Hanneke Pullens, Barbara van Munster, Bianka Mennema, Alie Haze-Visser, Agnes van 't Hof, Mariët Dirkzwager, Stella de Regt, Lisette Bruns, Suzan Vroomen, Jolanda Maaskant, Rosanne van Seben, Suzanne Geerlings and Bianca Buurman

Key words: Discharge Bundle, Patient Handovers, Patient Safety, Interrupted Time Series, Quality Improvement

Word count: 3214

For peer review only - http://bmjopen.bmj.com/site/about/guidelines.xhtml

Abstract

Objective Patient handovers are often delayed, patients are hardly involved in their discharge process and hospital-wide standardized discharge procedures are lacking. The aim of this study was to implement a structured discharge bundle and to test the effect on timeliness of medical and nursing handovers, length of hospital stay (LOS) and unplanned readmissions.

Design Interrupted time series with six pre-intervention and six post-intervention data collection points (September 2015 through June 2017).

Setting Internal medicine and surgical wards.

Participants Patients (≥18 years) admitted for more than 48h to surgical or internal medicine wards.

Intervention The Transfer Intervention Procedure (TIP), containing four elements: planning the discharge date within 48h post-admission, arrangements for postdischarge care, preparing handovers and personalized patient discharge letter; and a discharge conversation 12-24h before discharge.

Outcome measures The number of medical and nursing handovers sent within 24h. Secondary outcomes were median time between discharge and medical handovers, LOS and unplanned readmissions.

Results Pre-intervention 1039 and post-intervention 1052 patient records were reviewed. No significant change was observed in the number of medical and nursing handovers sent within 24h. The median (interquartile range) time between discharge and medical handovers decreased from 6.15 (0.96-15.96) to 4.08 (0.33-13.67) days, but no significant difference was found. No intervention-effect was observed for LOS and readmission. In subgroup analyses, a reduction of 5.6 days in the median time between discharge and medical handovers was observed in hospitals with high protocol adherence and much attention for implementation.

Conclusion Implementation of a structured discharge bundle did not lead to improved timeliness of patient handovers. However, large inter-hospital variation was observed and an intervention effect

For peer review only - http://bmjopen.bmj.com/site/about/guidelines.xhtml

on the median time between discharge and medical handovers was seen in hospitals with high protocol adherence. Future interventions should continue to create awareness of the importance of timely handovers.

Trial Registration: Dutch Trial Registry: NTR5951

Strengths and limitations of this study

- This study aimed to implement a structured discharge bundle to improve patient handovers for every patient.
- The study design, i.e. Interrupted Time Series Analysis, provided valuable information on pre-intervention trends, which strengthens the results.
- Sensitivity analysis provided important insight into the inter-hospital variation and differences in intervention effects among hospitals.
- Only the date of sending patient handovers were recorded. Knowing whether the next care provider received information would have been informative.
- It was not possible to evaluate percentages of compliance with the study protocol. Therefore, the process evaluation with the project leaders might have been an overestimation.

For peer review only

For peer review only - http://bmjopen.bmj.com/site/about/guidelines.xhtml

Introduction

As hospital stays have become shorter and full recovery often takes place at home,[1] a safe transition from hospital to home or nursing home has become more and more important. Besides, a rising number of older chronically ill patients who move within the health care system, requires continuity of care[2 3]. However, transitions from hospital to primary care settings are still considered a high-risk process. Patients are discharged with little coordination or follow-up and are hardly involved in their own discharge process[4 5].

Inadequate transitions may have serious implications for patient safety and quality of care. Postdischarge adverse events such as medication errors, can be the consequence of insufficient or lacking communication between hospital and primary care providers, thereby contributing to higher resource use and unplanned readmission rates[6-11]. In fact, unplanned readmission rates in the first month postdischarge are as high as 20%[12] and a recent study shows that half of them are deemed preventable[11].

The root of a safe transition from hospital to home or nursing home is a timely transfer of the medical handover, that is a letter containing accurate medical discharge information for the next care provider[8 13]. The general practitioner (GP) can only take over responsibility for a patient safely, when receiving a medical handover containing accurate information on, e.g., medications, and follow-up[13]. Nonetheless, a review of Kripalani et al. showed that medical handovers are often not available, lack important information or are not sent in a timely manner[8]. Also, a more recent study performed in 20 Dutch hospitals showed that in 10% of cases medical handover were missing and the remainder was on average sent after one week,[14] even though unplanned readmissions most frequently occur within the first week postdischarge[15].

Previous studies that aimed to improve patient handovers, mainly focused on specific high risk populations and targeted patient-related factors[16-18]. Although such interventions on individualized discharge planning or transitional care have been effective in reducing readmission[16 17]

For peer review only - http://bmjopen.bmj.com/site/about/guidelines.xhtml

and postdischarge mortality rates,[18-20] organizational factors that form the basis of a safe handover should also be optimally arranged[13 21]. In fact, in order to ensure patient safety and continuity of care, early discharge planning, a structured discharge process and timely handovers might be essential[13 21 22]. Besides, given that patients are often unprepared at time of discharge and uncertainties about aspects such as treatment or medication may exist,[5] patient education, e.g., in terms of a proper discharge conversation, should also be an important aspect of the discharge process[6 7].

The aim of this study was, therefore, to implement a structured discharge process, the Transfer Intervention Procedure (TIP), in eight hospitals. The TIP contains four elements: planning the discharge date within 48h after admission, arrangements for required postdischarge care, preparing medical, medication, and nursing handovers and a personalized discharge letter for the patient (PPDL) within 48h after admission; and holding a discharge conversation 12 to 24h before discharge. We tested whether the TIP improved timeliness of medical and nursing handovers and investigated the effect of the TIP procedure on length of hospital stay and unplanned readmissions within 30-days postdischarge.

Methods

Study design and setting

We evaluated the implementation of the TIP discharge bundle in an interrupted time series (ITS), which is the strongest design when a randomized controlled trial is not feasible[23 24]. The trial protocol[25] was based on the recommendations for ITS studies,[23] and we adhered to the SQUIRE guidelines for quality improvement reporting[26]. The current study was part of a large national program, initiated by the Dutch Ministry of Health, Welfare, and Sport (abbreviated in Dutch: VWS): 'Addressing Waste in Health Care'. This program was set up in order to reduce inefficiencies in the provision of health care. As part of this program, a TIP study group was established, comprising a study coordinator, two supervisors, one clinical epidemiologist, a policy officer from the Ministry of

For peer review only - http://bmjopen.bmj.com/site/about/guidelines.xhtml

VWS and local project leaders from the eight participating hospitals (one university and seven regional teaching throughout the Netherlands) that implemented the TIP bundle at one of their surgical and one of their internal medicine wards.

Within an interrupted time series, repeated observations are collected over time and divided into two segments, one before and one after implementation. Therefore, at six pre-intervention data collection points, measurements were conducted before implementation of the TIP and at six post-intervention data collection points measurements were conducted after implementation. During the implementation period of two months no measurements were conducted. February 2016, a kick off meeting was held. Between March 2016 and November 2016, hospitals started with implementation. Data collection started September 2015 and ended June 2017 (Supplement Table 1). All patients (aged ≥18 years) admitted for more than 48h were eligible for inclusion. The Medical Ethics Research Committee (METC) confirmed that the Medical Research Involving Human Subjects Act did not apply to this research project and official approval was not required. Since the study involved a quality improvement intervention with negligible risk of harming patients, individual informed consent was waived for all participating hospitals by the legal department research support of the Amsterdam UMC, location AMC. This trial was registered with the Dutch Trial Registry number NTR5951.

The discharge process in the Netherlands

In the Netherlands, primary care standards are relatively high and basically every person has a general practitioner (GP). When a person is hospitalized, responsibility is taken over from the GP by the medical specialist. After discharge, patient care becomes the responsibility of the GP again. It is policy for hospitals to provide patient handovers to the GP. However, there are no clear guidelines for hospitals how to arrange their discharge process. The Dutch healthcare inspectorate,[27] indicated that standardized discharge processes are lacking and errors that occur during handovers are often resolved informally.

For peer review only - http://bmjopen.bmj.com/site/about/guidelines.xhtml

After discharge from the hospital, the hospital physician sends a medical handover to the primary care provider for every patient (e.g., nursing home physician or the GP). Medical handovers include information on the reason for admission, diagnosis, comorbidity, the course of admission, medical examinations, treatment, medication, the health status of the patient at discharge, and instructions on follow-up[28]. Nursing handovers are only provided when the patient is discharged to a nursing home or with postdischarge care at the patient's own home. Nursing handovers include information on the care provided during hospitalization, current nursing care problems, the reason why (nursing) home care is initiated, and the intended outcomes of the care that will be provided[29].

Intervention

Figure 1 (adapted from van Seben et al.[30]) illustrates how the TIP bundle forms the basis of a safe handover from hospital to primary care for every patient, and if applicable, for patients discharged with postdischarge care (e.g., home care or a nursing home) or for complex patients who require a case manager or transitional care. As described in two previous studies,[25 31] the TIP bundle was developed using input from focus group meetings with professionals, patient surveys and literature. The TIP discharge bundle consists of four elements: 1) planning the discharge date within 48h after admission and communication of the discharge date with the patient, 2) starting with arrangements for required postdischarge care within 48h after admission; 3) preparing patient handovers (medical, medication, nurse) and personalized patient discharge letter (PPDL[32]) within 48h after admission, 4) planning a discharge conversation with the patient to explain information from the PPDL 12-24h before discharge. The PPDL is a standardized document, containing understandable information for the patient on the reason for admission, hospital treatment, course of the disease, possible sustained consequences or complications, and information on medication. We constructed checklists based on the TIP, which served as remembering tool for nurses and physicians in the electronic system or on pocket cards.

For peer review only - http://bmjopen.bmj.com/site/about/guidelines.xhtml

Patient and public Involvement

Our research question was developed from the perspective that patients are discharged with little coordination or follow-up and that they are often unprepared at time of discharge[4 5]. Patients were involved as participants in the construction of the TIP discharge bundle, which was based on, among others, patient satisfaction surveys[25 31]. Further, in a previous study in which the PPDL was developed and implemented, patient satisfaction with the PPDL was also assessed[32].

Protocol adherence

To enhance intervention fidelity and protocol adherence in the different hospitals, regular meetings were held with the TIP study group to report results and provide feedback, to discuss implementation, share experience and learn from each other's practices. A process evaluation was conducted with the project leaders to investigate protocol adherence, implementation strategies and attention paid to implementation. Elements that were considered included leadership and education of project leaders, projects group, extent of implementation of the discharge bundle, and education of physicians and nurses. Feedback points were awarded for all elements and for the extent to which the hospital complied to a certain element, e.g., for every person present at the kick off meeting or for every project meeting that was held. When a hospital partly complied to an element, e.g. automatically generated discharge summaries were provided to the patient instead of a PPDL or feedback on timely handovers was only provided to nurses, 0.5 feedback points were awarded. It was not possible to evaluate percentages of compliance with discharge conversations, planning discharge dates and arrangement of postdischarge care within 48h since these aspects were not reported in patient records. Hospital policies regarding these elements were assessed.

Outcome measures

For peer review only - http://bmjopen.bmj.com/site/about/guidelines.xhtml

Our primary outcome was the number of medical and nursing handovers sent within 24h. This time-frame was based on a report of the Dutch healthcare inspectorate (In Dutch: Inspectie voor de Gezondheidszorg en Jeugd (IGJ)) on the discharge process and handovers, in which it is stated that accurate information needs to be available as quick as possible, but certainly within 24h, for the next care provider[27]. Medical handovers also include medication handovers and we considered the time that these handovers were sent to the GP. The median time between discharge and the medical handover was considered as secondary outcome. Further, secondary outcomes were length of hospital stay (LOS) and rates of unplanned readmission within 30 days.

Baseline data collection

Data regarding patient characteristics included: demographics, admission ward and medical data (i.e. presence of polypharmacy, comorbidity,[33] number of hospitalization in the six months prior to current hospitalization). Variables were all collected from patient files. All data were reported and analyzed anonymously.

Sample size calculation

Based on the findings of a previous study[31] we expected to find a reduction of 78% in the time between discharge and medical handovers sent. We conducted a power analysis with a number of patients based on the number of hospital beds at the participating wards and feasibility with regards to data collection, which was set at 11 patients. In a simulation study with 16 wards, each contributing 65 patients, we estimated the power to be approximate 91% to demonstrate a reduction of 78% in time until sending the medical handover, assuming that the intraclass correlation coefficient does not exceed 0.05.

Statistical analysis

For peer review only - http://bmjopen.bmj.com/site/about/guidelines.xhtml

Descriptive characteristics of patients were calculated using proportions, means and standard deviations (SD), or medians and interquartile ranges (IQR), as appropriate. Chi-squared analysis and the Mann Whitney test were used to compare pre-intervention and post-intervention patient characteristics. Our time series was divided into two segments, one before and one after implementation of the TIP and we used segmented regression analysis to detect post-intervention level changes (i.e., an immediate change in the observed outcome after implementation) and changes in post-intervention trends relative to pre-intervention trends (i.e., a change in slopes of the regression lines after implementation). A least square regression line was fitted to the two segments of the continuous time variable. The segmented regression helped us to estimate the change in the intercept and the slope coefficients between the pre-intervention and post-intervention period using the following model: $Y_t = \alpha + \beta_1\ time_t + \beta_2\ intervention_t + \beta_3\ time\ after\ intervention_t + \varepsilon_t$. Since observations over time are correlated, we explored models with no, a first order autoregressive correlation between consecutive data collection periods, and longer autocorrelation structures.[24] We used the Akaike Information Criterion (AIC) as an estimator of the relative quality of a model and we report the results from the best fitting model. Correction for baseline imbalances as potential confounders led to results with similar estimates and identical interpretation. Based on the extent of protocol adherence and the feedback points awarded, subgroup analyses were performed to assess the intervention effect on the number of medical handovers within 24h and the median time between discharge and medical handovers. Statistical analyses were performed using SPSS Statistics ©, version 24.0, and Rstudio, version 1.0.136 (© 2009 – 2016 Rstudion, Inc).

Results

A total of 2091 patient records (1039 pre- and 1052 post-intervention) were reviewed in order to investigate the effect of the Transfer Intervention Procedure (TIP) on the timeliness of medical and nursing handovers, length of hospital stay (LOS) and unplanned readmission within 30 days. Overall

For peer review only - http://bmjopen.bmj.com/site/about/guidelines.xhtml

patients had a mean age (SD) of 68.1 (16.6) and 46.4% were male (table 1). There were significant differences between the pre-, and post-intervention group with regard to polypharmacy and the ratio of acute/elective hospitalizations and these variables were considered as potential confounders. However, correction for these potential confounders did not provide better models than the presented models.

Protocol adherence

Implementation strategies and protocol adherence are summarized in Supplement Table 1. Based on the process evaluation, three subgroups were identified. Subgroup 1 (hospitals 4 and 8), >30 feedback points, paid considerable attention to implementation and there was relatively high protocol adherence. In subgroup 2 (hospitals 1-3, and 5), 20-30 feedback points, there was relatively high protocol adherence but moderate attention to implementation. In subgroup 3 (hospitals 6 and 7), <10 feedback points, nearly no attention was brought to implementation and there was low compliance.

Medical and nursing handovers

In the total study population, no intervention effect was found on the percentage of medical handovers being sent within 24h after hospital discharge to the GP: 22.7% medical handovers were sent within 24h pre-intervention, 29.1% post-intervention and no significant difference was observed in the levels and trends between the pre-intervention and post-intervention period. The median (interquartile range, IQR) time between discharge and medical handovers decreased from 6.15 (0.96-15.96) days, pre-intervention to 4.08 (0.33-13.67) days post-intervention. An absolute effect directly after the implementation of the intervention of -0.25 days was found (i.e., de difference in time between discharge and medical handovers between the sixth pre-intervention data collection point and first post-intervention data collection point). We observed no significant difference in the levels

For peer review only - http://bmjopen.bmj.com/site/about/guidelines.xhtml

and trends. The number of nursing handovers sent within 24h postdischarge was 92.8% pre-intervention and 93.1% post-intervention and no significant difference was observed between levels and trends. The results are presented in Figure 2 and the parameters estimates are summarized in Table 2.

Length of hospital stay and unplanned readmission rates

No significant decline in the levels and trends between the pre-, and post-intervention was found with regard to LOS (β 0.08, 95% CI -0.12 to 0.29 p=0.45) and unplanned readmission rates (β 1.11, 95% CI -2.55 to 0.33 p=0.17). Median (IQR) LOS was 8.17 (4.75-15.13) and 8.56 (4.88-15.91) days and readmissions rates were as high as 11.1% and 12.3% pre-intervention and post-intervention, respectively. With regard to LOS, the results are adjusted for autocorrelation (AIC 22.64 versus 33.75, p=0.01), but not for potential confounders (AIC 43.08 versus 33.75, p=0.07). With regard to unplanned readmission rates, the results are unadjusted for autocorrelation (AIC 57.18 versus 54.45, p=0.10) and potential confounders (AIC 57.47 versus 54.45, p=0.61).

Subgroup analysis

In subgroup 1 (>30 feedback points), an absolute effect of 13.3% more medical handovers sent within 24h postdischarge was observed but this did not result in significant changes in level or trends (Figure 3). A reduction of 5.6 days in the median time between discharge and handovers with a significant change in level directly after the intervention was observed in subgroup 1 (β -5.29, 95% CI -8.70 to 1.87 p=0.02). Pre-intervention, group 2 (20-30 feedback points) had the highest rate of medical handovers sent within 24h and the lowest median time between discharge and medical handovers but no intervention effect was observed. Both pre- and post-intervention, subgroup 3 (<10 points) had the lowest rates of medical handovers sent within 24h, and the highest median time. We observed no intervention effect in subgroup 3.

For peer review only - http://bmjopen.bmj.com/site/about/guidelines.xhtml

Discussion

In the total study population, a structured discharge bundle, the Transfer Intervention Procedure (TIP), did not lead to improved timeliness of medical and nursing handovers. Although medical handovers were sent faster post-intervention (pre-intervention median 6.15; post-intervention 4.08 days), we were unable to show significant differences in level and trends, both with regard to the median time and the number of medical handovers sent within 24h. However, large inter-hospital variation was observed and a significant intervention effect on the median time between discharge and medical handovers was seen in those hospitals with relatively high protocol adherence and attention for implementation. Rates of nursing handovers sent within 24h were both pre- and post-intervention above 90%. No intervention effect was found for length of hospital stay (LOS) and readmissions.

Extensive research has been conducted to improve patient handovers from hospital to home[7-16]. Summarizing findings of earlier discharge interventions that aimed to improve coordination of care and communication between hospital and primary care providers, Hesselink et al.,[7] and Kripalani et al.,[8] showed that some studies were able to improve timeliness of medical handovers. These interventions, however, were based on the introduction of fax, email or web-based transfers of information, which is increasingly becoming standard practice in Dutch hospitals. Yet, further improvement may lie in electronic sending systems that support the use of standardized formats that pull information from patient files into (medical) handovers or that send information to the next care provider automatically.

Although a before-after design would probably have led to a significant intervention-effect, the ITS analysis provided valuable information on pre-intervention trends. The observed median time between discharge and sending medical handovers at our first pre-intervention measurement point was consistent with a recent Dutch study[14], but a trend towards sending handovers faster was

For peer review only - http://bmjopen.bmj.com/site/about/guidelines.xhtml

already observed along the pre-intervention period. During the pre-intervention period, no interventions were implemented and the TIP was introduced and implemented during a two-month implementation period during which no measurements were conducted. However, in the pre-intervention period, attention was already brought to the discharge process, e.g. by establishing project groups and the kick-off meeting. Although these activities were not intended as implementation strategies, in hindsight they might explain why improvements were already observed during the pre-intervention period, particularly since education on the importance of the intervention is an important aspect of implementation[13 34 35].

Although positive trends in the pre-intervention period were less pronounced in the subgroup analysis, results of the separate analyses support the idea that attention is important. Whereas a significant reduction of six days in the median time between discharge and medical handovers was observed in hospitals that paid much attention to implementation, no intervention effect was observed in hospitals that paid moderate to nearly no attention. It should be noted that the hospitals that paid moderate attention had relatively good pre-intervention scores. A smaller window for improvement in these hospitals might also explain a lack of intervention effect[36].

Implementation of the TIP procedure did not reveal a reduction of LOS. Although a possible explanation can be low overall compliance with our study protocol, it is also plausible that over the past years, average LOS has decreased to a minimum[37]. Given current pressure on availability of hospital beds, patients are discharged as soon as possible. This may account for inadequate discharge processes, since physicians are forced to prioritize acute health care over discharge-related tasks[38 39].

Given increasingly shorter LOS[37] and the often complex care needs patient face, patient preparation should be an important aspect of the discharge process. In fact, the most effective discharge interventions seem to have educational components[40]. Unfortunately, given the workload among residents, implementation of a personalized patient discharge letter was unsuccessful. E.g., posing the question “do you feel ready to go home”[41] or postdischarge telephone contact,[7] might be

For peer review only - http://bmjopen.bmj.com/site/about/guidelines.xhtml

less time-consuming ways to involve patients. However, to prevent readmissions more effort might be necessary. Previous interventions that revealed a reduction in readmission rates, consist of individualized discharge planning or continue postdischarge[16 42]. However, we believe that a structured discharge process such as the TIP should form the basis for a safe handover for every patient (Figure 1).

Implications for further research

Our study shed light on the difficulties that come along with implementation of quality improvement collaboratives[43]. Given the positive pre-intervention trends and significant reduction in the median time between discharge and medical handovers in hospitals that paid much attention to implementation, further improvements may lie in interventions that create more awareness of the importance of timely handovers and hospital physicians' crucial role in the provision of continuity of care. This might stimulate physicians' intrinsic motivation to provide a structured discharge process and thereby timely handovers[7 39]. Furthermore, we might also want to focus on local factors that lead to insufficient discharge processes. A comprehensive exploration of local barriers for each step in the TIP discharge process might be helpful in order to develop tailor made interventions on a local or department level to improve the discharge process[44].

Limitations

An interrupted time series provides a strong quasi-experimental design to evaluate the impact of an intervention aimed at quality improvement. However, this study design also has limitations. First of all, a positive trend towards sending handovers faster along the pre-intervention period, which was probably due to the attention that was already brought to the discharge process before implementation of the discharge bundle. In fact, an important limitation of ITS is that it is more difficult to determine whether the observed effect is a direct effect of the intervention, in contrast to

For peer review only - http://bmjopen.bmj.com/site/about/guidelines.xhtml

e.g., clustered trials. Second, medical staff was not blinded for the outcome measure, that is timely discharge letters. Knowing that timeliness of discharge letters was monitored might have altered our results. However, in most hospitals timeliness of discharge letters was already monitored before we started with our research project and the effect is likely to be minimal. Third, we only recorded the date of sending medical handovers. Knowing whether they were received by GPs would also have provided valuable information. Fourth, we did not look at the content of handovers, while this might have given us important insights. Lastly, it was not possible to evaluate percentages of protocol adherence and the process evaluation with the project leaders might have been an overestimation. However, the process evaluation was in line with the efforts observed during implementation.

Conclusion

Implementation of a structured discharge bundle, the Transfer Intervention Procedure (TIP), did not lead to more medical and nursing handovers sent within 24h postdischarge. Large inter-hospital variation was observed however, and a significant intervention effect on the median time between discharge and medical handovers was seen in those hospitals with high protocol adherence and that brought much attention to implementation. We believe that future interventions should continue to create awareness of the importance of timely handovers and we hope that our study contributes to this, stimulating hospitals to further structure and improve their discharge process.

Contributors: BB, SG, and RVS designed the study. BB and SG, conceived the study and obtained funding. RVS collected data and JM and RVS performed statistical analysis. All authors contributed to drafting the manuscript or revised it critically and gave final approval for publication. The authors would like to acknowledge the contribution of the TIP study group, consisting in addition to the authors, of the following members Hanneke Pullens (Catharina Hospital Eindhoven), Barbara van Munster (Gelre Hospitals Apeldoorn), Annemarie van der Lugt (Haven Hospital Rotterdam), Alie

For peer review only - http://bmjopen.bmj.com/site/about/guidelines.xhtml

Haze-Visser (Lange Land Hospital Zoetermeer), Agnes van ‘t Hof (Maxima Medical Center Veldhoven), Mariët Dirkzwager (OLVG Amsterdam), Stella de Regt (Reinier de Graaf Hospital Delft), Suzan Vroomen (Amsterdam UMC, location AMC) and Lisette Bruns (Dutch Ministry of Health, Welfare, and Sport). Furthermore, we thank Rachel de Vries en Vera van Miltenburg for their assistance with data collection.

Funding sources: This work was supported by the Dutch Ministry of Health, Welfare and Sport [grant number: 324798].

Conflicts of interest: None declared.

Data sharing statement: No additional data are available.

For peer review only

For peer review only - http://bmjopen.bmj.com/site/about/guidelines.xhtml

References

1. Eurostat Statistics Explained. Hospital discharges and length of stay statistics 2016 [Available from: http://ec.europa.eu/eurostat/statistics-explained/index.php/Hospital_discharges_and_length_of_stay_statistics accessed October 17 2017.
2. Jean Hall M, DeFrances CJ, Williams SN, Golonsinskiy MS, Schwartzman A. National Health Statistics Reports. *US Department of Health and Guman Services Centers for Disease Control and Prevention National Center for Health Statistics* 2010;29
3. Boyd CM, Darer J, Boult C, Fried LP, Boult L, Wu AW. Clinical practice guidelines and quality of care for older patients with multiple comorbid diseases: implications for pay for performance. *Jama* 2005;294(6):716-24. doi: 10.1001/jama.294.6.716 [published Online First: 2005/08/11]
4. Henderson A, Zernike W. A study of the impact of discharge information for surgical patients. *Journal of advanced nursing* 2001;35(3):435-41. [published Online First: 2001/08/08]
5. Holland DE, Mistiaen P, Bowles KH. Problems and unmet needs of patients discharged "home to self-care". *Professional case management* 2011;16(5):240-50; quiz 51-2. doi: 10.1097/NCM.0b013e31822361d8 [published Online First: 2011/08/19]
6. Greenwald JL, Jack BW. Preventing the preventable: reducing rehospitalizations through coordinated, patient-centered discharge processes. *Professional case management* 2009;14(3):135-40; quiz 41-2. doi: 10.1097/NCM.0b013e318198d4e1 [published Online First: 2009/05/29]
7. Hesselink G, Schoonhoven L, Barach P, et al. Improving patient handovers from hospital to primary care: a systematic review. *Annals of internal medicine* 2012;157(6):417-28. doi: 10.7326/0003-4819-157-6-201209180-00006 [published Online First: 2012/09/19]
8. Kripalani S, LeFevre F, Phillips CO, Williams MV, Basaviah P, Baker DW. Deficits in communication and information transfer between hospital-based and primary care physicians: implications for patient safety and continuity of care. *Jama* 2007;297(8):831-41. doi: 10.1001/jama.297.8.831 [published Online First: 2007/03/01]
9. Forster AJ, Murff HJ, Peterson JF, Gandhi TK, Bates DW. The incidence and severity of adverse events affecting patients after discharge from the hospital. *Annals of internal medicine* 2003;138(3):161-7. [published Online First: 2003/02/01]
10. Moore C, Wisnivesky J, Williams S, McGinn T. Medical errors related to discontinuity of care from an inpatient to an outpatient setting. *Journal of general internal medicine* 2003;18(8):646-51. [published Online First: 2003/08/13]
11. Fluitman KS, van Galen LS, Merten H, et al. Exploring the preventable causes of unplanned readmissions using root cause analysis: Coordination of care is the weakest link. *European journal of internal medicine* 2016;30:18-24. doi: 10.1016/j.ejim.2015.12.021 [published Online First: 2016/01/18]
12. Kansagara D, Englander H, Salanitro A, et al. Risk prediction models for hospital readmission: a systematic review. *Jama* 2011;306(15):1688-98. doi: 10.1001/jama.2011.1515 [published Online First: 2011/10/20]
13. Merten H, van Galen LS, Wagner C. Safe handover. *BMJ (Clinical research ed)* 2017;359:j4328. doi: 10.1136/bmj.j4328 [published Online First: 2017/10/11]
14. Langelaan M, Baines RJ, de Bruijne MC, Wagner C. Association of admission and patient characteristics with quality of discharge letters: posthoc analysis of a retrospective study. *BMC health services research* 2017;17(1):225. doi: 10.1186/s12913-017-2149-8 [published Online First: 2017/03/23]
15. Dharmarajan K, Hsieh AF, Lin Z, et al. Diagnoses and timing of 30-day readmissions after hospitalization for heart failure, acute myocardial infarction, or pneumonia. *Jama* 2013;309(4):355-63. doi: 10.1001/jama.2012.216476 [published Online First: 2013/01/24]

For peer review only - http://bmjopen.bmj.com/site/about/guidelines.xhtml

16. Verhaegh KJ, MacNeil-Vroomen JL, Eslami S, Geerlings SE, de Rooij SE, Buurman BM. Transitional care interventions prevent hospital readmissions for adults with chronic illnesses. *Health affairs (Project Hope)* 2014;33(9):1531-9. doi: 10.1377/hlthaff.2014.0160 [published Online First: 2014/09/10]
17. Prvu Bettger J, Alexander KP, Dolor RJ, et al. Transitional care after hospitalization for acute stroke or myocardial infarction: a systematic review. *Annals of internal medicine* 2012;157(6):407-16. doi: 10.7326/0003-4819-157-6-201209180-00004 [published Online First: 2012/09/19]
18. Shepperd S, Lannin NA, Clemson LM, McCluskey A, Cameron ID, Barras SL. Discharge planning from hospital to home. *The Cochrane database of systematic reviews* 2013;1:Cd000313. doi: 10.1002/14651858.CD000313.pub4 [published Online First: 2013/02/27]
19. Buurman BM, Parlevliet JL, Allore HG, et al. Comprehensive Geriatric Assessment and Transitional Care in Acutely Hospitalized Patients: The Transitional Care Bridge Randomized Clinical Trial. *JAMA internal medicine* 2016;176(3):302-9. doi: 10.1001/jamainternmed.2015.8042 [published Online First: 2016/02/18]
20. Naylor MD, Brooten DA, Campbell RL, Maislin G, McCauley KM, Schwartz JS. Transitional care of older adults hospitalized with heart failure: a randomized, controlled trial. *Journal of the American Geriatrics Society* 2004;52(5):675-84. doi: 10.1111/j.1532-5415.2004.52202.x [published Online First: 2004/04/17]
21. The Joint Commission. Hot Topics in Health Care. Transitions of Care: The need for a more effective approach to continuing patient care. *The Joint Commission* 2012
22. Kripalani S, Jackson AT, Schnipper JL, Coleman EA. Promoting effective transitions of care at hospital discharge: a review of key issues for hospitalists. *Journal of hospital medicine* 2007;2(5):314-23. doi: 10.1002/jhm.228 [published Online First: 2007/10/16]
23. Jandoc R, Burden AM, Mamdani M, Levesque LE, Cadarette SM. Interrupted time series analysis in drug utilization research is increasing: systematic review and recommendations. *Journal of clinical epidemiology* 2015;68(8):950-6. doi: 10.1016/j.jclinepi.2014.12.018 [published Online First: 2015/04/22]
24. Penfold RB, Zhang F. Use of interrupted time series analysis in evaluating health care quality improvements. *Academic pediatrics* 2013;13(6 Suppl):S38-44. doi: 10.1016/j.acap.2013.08.002 [published Online First: 2013/12/07]
25. van Seben R, Geerlings SE, Verhaegh KJ, Hilders CG, Buurman BM. Implementation of a Transfer Intervention Procedure (TIP) to improve handovers from hospital to home: interrupted time series analysis. *BMC health services research* 2016;16:479. doi: 10.1186/s12913-016-1730-x [published Online First: 2016/09/09]
26. Davidoff F, Batalden P, Stevens D, Ogrinc G, Mooney SE. Publication guidelines for quality improvement studies in health care: evolution of the SQUIRE project. *BMJ (Clinical research ed)* 2009;338:a3152. doi: 10.1136/bmj.a3152 [published Online First: 2009/01/21]
27. Dutch Health Care Inspectorate (IGZ). Continuïteit van zorg voor kwetsbare ouderen vanuit het ziekenhuis naar verpleeg- en verzorgingshuizen, thuiszorg en huisartsen niet gewaarborgd. . 2015
28. Joint Commission on Accreditation of Healthcare Organizations. Accreditation Standards for Hospitals. Standard ACC.3 Oakbrook Terrace, IL: The Joint Commission 2010.
29. V&VN. Richtlijn Verpleegkundige en verzorgende verslaglegging September 2011 [
30. van Seben R, Geerlings SE, Buurman BM. [10 tips for improving patient handovers from hospital to other health care settings]. *Ned Tijdschr Geneeskd* 2017;161(0):D1031. [published Online First: 2017/04/27]
31. Verhaegh KJ, Buurman BM, Veenboer GC, de Rooij SE, Geerlings SE. The implementation of a comprehensive discharge bundle to improve the discharge process: a quasi-experimental

For peer review only - http://bmjopen.bmj.com/site/about/guidelines.xhtml

study. *The Netherlands journal of medicine* 2014;72(6):318-25. [published Online First: 2014/10/17]
32. Buurman BM, Verhaegh KJ, Smeulers M, et al. Improving handoff communication from hospital to home: the development, implementation and evaluation of a personalized patient discharge letter. *International journal for quality in health care : journal of the International Society for Quality in Health Care / ISQua* 2016;28(3):384-90. doi: 10.1093/intqhc/mzw046 [published Online First: 2016/05/27]
33. Charlson ME, Pompei P, Ales KL, MacKenzie CR. A new method of classifying prognostic comorbidity in longitudinal studies: development and validation. *Journal of chronic diseases* 1987;40(5):373-83. [published Online First: 1987/01/01]
34. Michie S, van Stralen MM, West R. The behaviour change wheel: a new method for characterising and designing behaviour change interventions. *Implementation science : IS* 2011;6:42. doi: 10.1186/1748-5908-6-42 [published Online First: 2011/04/26]
35. Spoorenberg V, Geerlings SE, Geskus RB, de Reijke TM, Prins JM, Hulscher ME. Appropriate antibiotic use for patients with complicated urinary tract infections in 38 Dutch Hospital Departments: a retrospective study of variation and determinants. *BMC infectious diseases* 2015;15:505. doi: 10.1186/s12879-015-1257-5 [published Online First: 2015/11/11]
36. Ivers N, Jamtvedt G, Flottorp S, et al. Audit and feedback: effects on professional practice and healthcare outcomes. *The Cochrane database of systematic reviews* 2012(6):Cd000259. doi: 10.1002/14651858.CD000259.pub3 [published Online First: 2012/06/15]
37. Coppa Consultancy. Ligduurmonitor Nederlandse ziekenhuizen 2013 & 2014. Trendbreuk in de ligduur. 2015 [Available from: http://www.coppa.nl/wp-content/uploads/2016/01/Ligduurmonitor-2013-2014.pdf accessed November, 2017.
38. Greysen SR, Schiliro D, Horwitz LI, Curry L, Bradley EH. "Out of sight, out of mind": housestaff perceptions of quality-limiting factors in discharge care at teaching hospitals. *Journal of hospital medicine* 2012;7(5):376-81. doi: 10.1002/jhm.1928 [published Online First: 2012/03/02]
39. Hesselink G, Vernooij-Dassen M, Pijnenborg L, et al. Organizational culture: an important context for addressing and improving hospital to community patient discharge. *Medical care* 2013;51(1):90-8. doi: 10.1097/MLR.0b013e31827632ec [published Online First: 2012/11/08]
40. Mistiaen P, Francke AL, Poot E. Interventions aimed at reducing problems in adult patients discharged from hospital to home: a systematic meta-review. *BMC health services research* 2007;7:47. doi: 10.1186/1472-6963-7-47 [published Online First: 2007/04/06]
41. van Galen LS, Brabrand M, Cooksley T, et al. Patients' and providers' perceptions of the preventability of hospital readmission: a prospective, observational study in four European countries. *BMJ quality & safety* 2017 doi: 10.1136/bmjqs-2017-006645 [published Online First: 2017/06/24]
42. Leppin AL, Gionfriddo MR, Kessler M, et al. Preventing 30-day hospital readmissions: a systematic review and meta-analysis of randomized trials. *JAMA internal medicine* 2014;174(7):1095-107. doi: 10.1001/jamainternmed.2014.1608 [published Online First: 2014/05/14]
43. Hulscher ME, Schouten LM, Grol RP, Buchan H. Determinants of success of quality improvement collaboratives: what does the literature show? *BMJ quality & safety* 2013;22(1):19-31. doi: 10.1136/bmjqs-2011-000651 [published Online First: 2012/08/11]
44. Flottorp SA, Oxman AD, Krause J, et al. A checklist for identifying determinants of practice: a systematic review and synthesis of frameworks and taxonomies of factors that prevent or enable improvements in healthcare professional practice. *Implementation science : IS* 2013;8:35. doi: 10.1186/1748-5908-8-35 [published Online First: 2013/03/26]

For peer review only

For peer review only - http://bmjopen.bmj.com/site/about/guidelines.xhtml

Table 1. Baseline characteristics

**Variable**	**Overall (N=2091)**	**Pre-intervention (N=1039)**	**Post-intervention (N=1052)**
**Age in years,** mean (SD)[a]	68.07 (16.57)	67.66 (16.70)	68.48 (16.45)
**Male,** No. (%)	971 (46.4)	493 (47.4)	478 (45.4)
**Living arrangements before admission,** No.(%)			
Independent	1814 (86.7)	883 (84.9)	931 (88.5)
Nursing home	49 (2.3)	27 (2.6)	22 (2.1)
Senior residence/Assisted living	168 (8.1)	91 (8.8)	77 (7.3)
Missing	60 (2.9)	38 (3.7)	22 (2.1)
**Marital status,** No. **(%)**			
Married or living together	1125 (53.8)	556 (53.5)	569 (54.1)
Single or divorced	456 (21.8)	212 (20.4)	244 (23.2)
Widow/widower	435 (20.8)	224 (21.6)	211 (20.1)
Missing	75 (3.6)	47 (4.5)	28 (2.7)
**Charlson Comorbidity Index** [b] (mean, SD [a])	2.05 (2.05)	2.10 (2.08)	2.01 (2.03)
**Polypharmacy,** No. (%)[c, d, e]	1247 (59.6)	586 (56.4)	661 (62.8)
Missing	12 (.6)	8 (.8)	4 (.4)
**Hospitalization in past 6 months,** No. (%)	705 (33.7)	339 (32.6)	336 (34.8)
**Acute hospitalization,** No. (%)[c, f]	73.0 (73.0)	725 (69.8)	801 (76.1)
**Admission ward, internal medicine** No. (%)	1051 (50.3)	524 (50.4)	527 (50.1)
**Discharge destination,** No. (%)			
Home	1551 (74.2)	770 (74.1)	781 (74.2)
Other health care setting, of which	482 (23.1)	238 (23.0)	244 (23.2)
Rehabilitation center	268 (12.8)	120 (11.5)	148 (14.1)
Nursing home	158 (7.6)	80 (7.7)	78 (7.4)
Assisted living	34 (1.6)	26 (2.5)	8 (0.8)
Other hospital	22 (1.1)	12 (1.2)	10 (1.0)
Missing	58 (2.8)	31 (3.0)	27 (2.6)

[a] Standard Deviation, [b] Range of 0 to 31, with a higher score indicating more or more severe comorbidity[33], [c] Use of 5 or more different medications, [d] Chi-Square, [e] P-value = 0.004, [f] P-value = 0.001

For peer review only

For peer review only - http://bmjopen.bmj.com/site/about/guidelines.xhtml

Table 2. Interrupted time series analysis; medical and nursing handovers

	Medical handovers <24 hrs after discharge (%) [a]			***Time between discharge and medical letter (days)*** [b]			***Nursing handovers <24 hrs after discharge (%)*** [c]		
	**β (SE)**	**95% CI**	**p-value**	**β (SE)**	**95% CI**	**p-value**	**β (SE)**	**95% CI**	**p-value**
Intercept	17.51 (3.79)	10.08 to 24.93	<0.01	7.20 (0.29)	6.63 to 7.76	<0.01	91.85 (2.71)	86.53 to 97.16	<0.01
Trend pre-intervention (β1)	1.49 (0.97)	-0.42 to 3.40	0.16	-0.30 (0.07)	-0.45 to -0.16	<0.01	0.28 (0.70)	-1.09 to 1.64	0.70
Level change directly after intervention (β2)	6.43 (10.13)	-13.43 to 26.28	0.54	-0.62 (0.74)	-2.07 to 0.84	0.43	6.32 (7.25)	-7.89 to 20.53	0.41
Trend differences (β3)	-0.94 (1.38)	-3.64 to 1.75	0.51	0.05 (0.10)	-0.14 to 0.25	0.61	-0.81 (0.99)	-2.74 to 1.12	0.43
	Absolute effect directly after intervention: -0.17%			Absolute effect directly after intervention: -0.25 days			Absolute effect directly after intervention: 0.62%		

β1 estimates the pre-intervention trend.
β2 estimates the difference between the observed level just after the intervention started and that predicted by the pre-intervention trend.
β3 estimates the difference in trend between the pre-intervention and post-intervention period.
SE: Standard Error, CI: Confidence Interval
[a] Correction for autocorrelation did not provide a better model compared to the presented model (AIC 74.17 versus 72.88, p=0.40), nor did correction for potential confounders ('polypharmacy' and 'acute admission') (AIC 74.98 versus 72.88, p=0.39). All models led to results with similar estimates and identical interpretation.
[b] The results are adjusted for autocorrelation, but not for potential confounders. Correction for autocorrelation (AR1) provided a better model compared to the presented model (AIC 21.52 versus 25.72, p=0.01). Correction for potential confounders ('polypharmacy' and 'acute admission') did not provide a better model compared to the presented model (AIC 29.23 versus 25.72, p=0.78). Correction for autocorrelation (AR1) changed β1 into a significant result. Correction for potential confounders did not alter the results.
[c] Correction for autocorrelation did not provide a better model compared to the presented model (AIC 66.05 versus 59.13, p=0.02), nor did correction for potential confounders ('polypharmacy' and 'acute admission') (AIC 59.03 versus 59.13, p=0.13). All models led to results with similar estimates and identical interpretation.

For peer review only

For peer review only - http://bmjopen.bmj.com/site/about/guidelines.xhtml

Figure 1. Pyramid for postdischarge care

A structured discharge process such as the TIP procedure should form the basis for every patient. For patients discharged with postdischarge care (20-25%), nursing handovers should be set up within 48h after admission and be sent within 24h postdischarge. Complex patients with a high readmission risk (10%) require a (nurse) case manager or transitional care in the transition from hospital to home.

Figure 2.

Panel A The number of medical handovers sent within 24 hours.

Panel B median time in days between discharge and the medical handovers.

Figure 3. Hospital differences based on implementation score.

The inter-hospital differences in rates of medical handovers being sent within 24h in the pre- and post-intervention based on the extent of implementation and used implementation strategies. Group 1 received >30 feedback implementation points, group 2 received 20-30 implementation points, group 3 received <20 points.

For peer review only

For peer review only - http://bmjopen.bmj.com/site/about/guidelines.xhtml

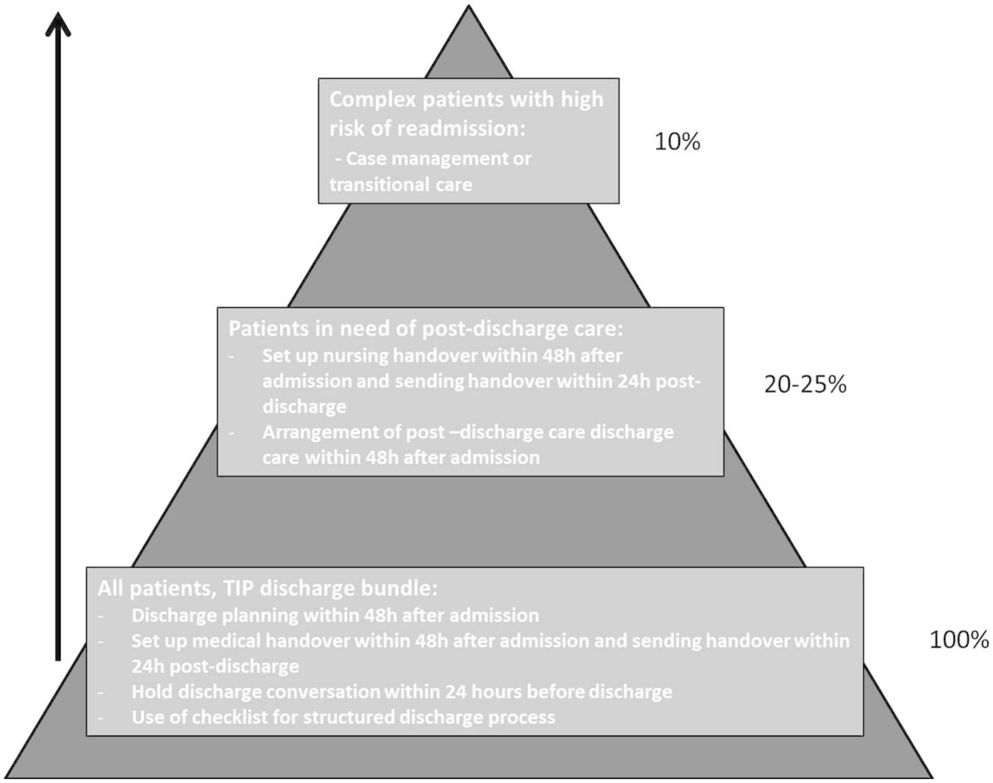

Figure 1. Pyramid for post-discharge care A structured discharge process such as the TIP procedure should form the basis for every patient. For patients discharged with post-discharge care (20-25%), nursing handovers should be set up within 48h after admission and be sent within 24h post-discharge. Complex patients with a high readmission risk (10%) require a (nurse) case manager or transitional care in the transition from hospital to home.

116x92mm (300 x 300 DPI)

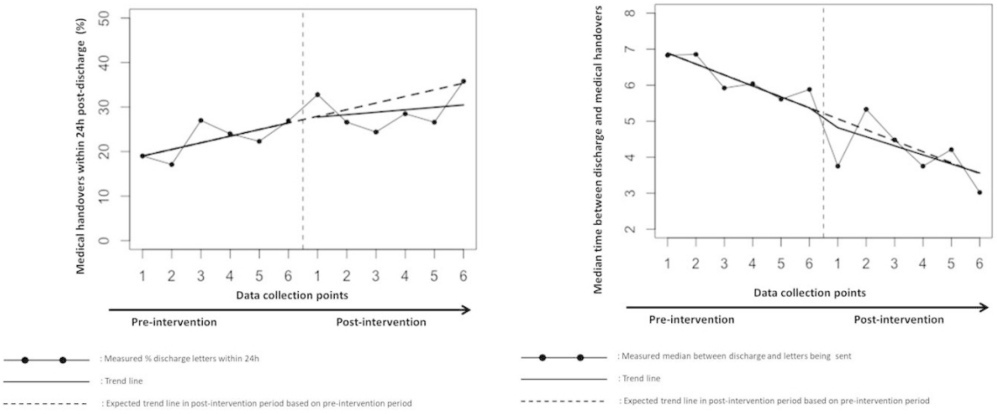

Figure 2.
Panel A The number of medical handovers sent within 24 hours.
Panel B median time in days between discharge and the medical handovers.

113x50mm (300 x 300 DPI)

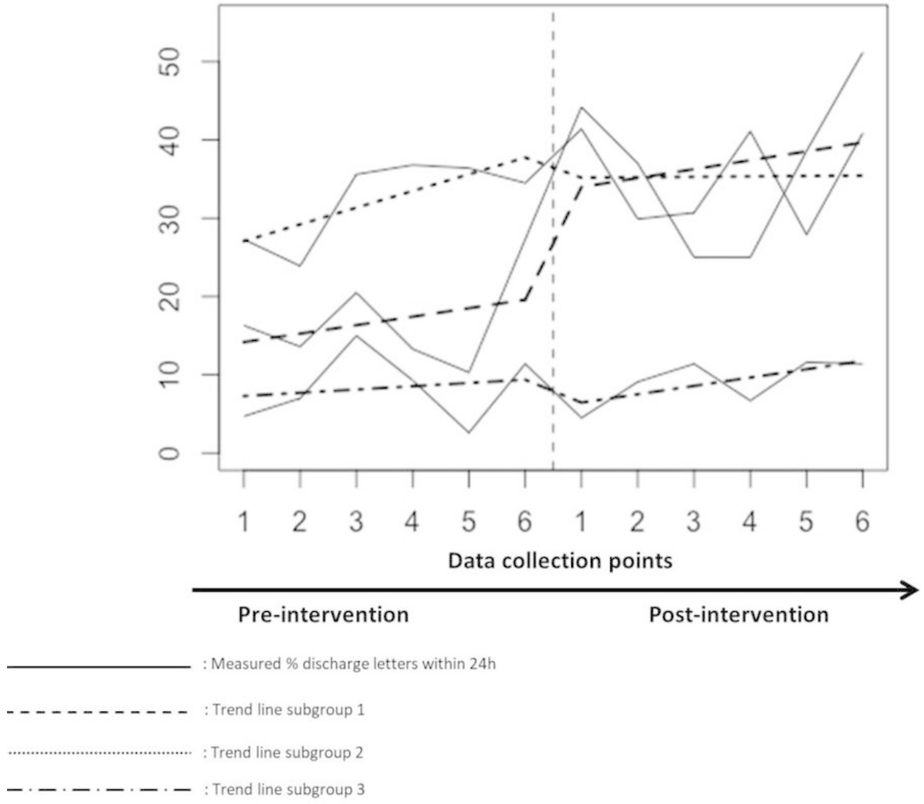

Figure 3. Hospital differences based on implementation score.
The inter-hospital differences in rates of medical discharge letters being sent within 24h in the pre- and post-intervention based on the extent of implementation and used implementation strategies. Group 1 received >30 feedback implementation points, group 2 received 20-30 implementation points, group 3 received <20 points.

82x69mm (300 x 300 DPI)

Supplement Table 1. Adherence to the Intervention Protocol

	Hospital 1	Hospital 2	Hospital 3	Hospital 4	Hospital 5	Hospital 6	Hospital 7	Hospital 8
Pre-intervention<br>Implementation<br>Post-intervention	Sep '15 - Feb '16<br>March '16 - April '16<br>May '16 – Oct '16	Oct '15 - March '16<br>April '16 - May '16<br>June '16 - Nov '16	Jan '16 - June '16<br>July '16 - Aug '16<br>Sep '16 – Feb '17	Dec '15 - May '16<br>June '16 - July '16<br>Aug '16 - Jan '16	March '16 – Aug '16<br>Sep '16 - Oct '16<br>Nov '16 – April '17	April '16 – Sep '16<br>Oct '16 - Nov '16<br>Dec '16 – May '17	May '16 – Oct '16<br>Nov '16 - Dec '16<br>Jan '17 – June '17	April '16 – Sep '16<br>Oct '16 - Nov '16<br>Dec '16 – May '17
**Leadership and education of project leaders**								
Who were present at the kick off meeting February, 2016?	Hospital president; local project leader; 2 team leaders (nurses); 2 physicians; nurse; pharmacists	Local project leader; geriatrician; head of the liaison department; physician	2 local project leaders; head of the liaison department	Chief of staff; local project leader; team leader surgery ward (nurse); head of the liaison department	Local project leader; head of the liaison department; manager patient logistics; 2 team leaders (nurses)	2 local project leaders	Local project leader	Local project leader; senior researcher transitional care; medical specialist,
Who were present at the first feedback session?	Project leader; head of the liaison department	Project leader; pharmacist; communication assistant	2 local project leaders; liaison nurse	Project leader; liaison nurse; nurse geriatrics	Local project leader	2 local project leaders	-	2 project leaders
Who were present at the second feedback session?	Project leader	Project leader	2 project leaders	-	Project leader	-	Project leader	2 project leaders
Implementation points	*10*	*8*	*8*	*7*	*7*	*4*	*2*	*7*
**Project group**								
Was there a local TIP project group, and who participated?	Yes, project leader; 2 senior nurses of participating wards, management assistant	Yes, project leader; geriatrician; head liaison department, physician; pharmacist; communication assistant; manager Security & Services	Yes, 2 project leaders; 2 residents; 2 medical specialists; nurse; liaison nurse; pharmacist; manager	Yes, chief of staff; project leader; 2 team leaders (nurses); head liaison department; orthopedist	Yes, project leader; head liaison department; 2 medical specialists, geriatrician	No	No	Yes, local project leader; 3 medical specialists; 2 residents, manager quality and safety; manager process optimization; medical director
How often did the local project group meet?<br>1 point per meeting	Monthly for 2 months, during pilot period every week (2 months).	Every five weeks during pre-intervention and pilot period	2 times, before pilot period.	Every two weeks during pre-intervention period	Every six weeks, during pre-intervention and pilot period	-	-	Monthly during pre-intervention, pilot period and first two months of post-

For peer review only

								intervention period
Implementation points	*10*	*12*	*10*	*18*	*10*	*0*	*0*	*17*
**Implementation of TIP elements**								
Was it policy to set a discharge date within 48h after admission?	Yes	Yes	Yes	Yes	Yes	Yes	Yes	Yes
Was the planned discharge date communicated to patients?	Yes	Yes	Yes	Yes	Yes	No	Yes	Yes
Was it policy to start with arrangement of required post-discharge care within 48h after admission?	No, liaison nurse has to wait for final discharge date	No, liaison nurse has to wait for final discharge date	Yes	Yes	No, liaison department is overloaded	Yes	No, liaison nurse has to wait for final discharge date	Yes
Was it policy to set up patient handovers within 48h after admission?	No	No	No	No	No	No	No	No
Did physicians hold discharge conversations, using a checklist during the pilot period?	No	No	No	No	No	No	No	No
Does the nurse holds a discharge conversation, using a checklist?	Yes	No	Yes	Yes	Yes	No	Yes	Yes
Was the patient discharge letter implemented?	Yes, but only for some diagnosis at internal medicine ward.	No	No	No	No	Yes , but only at internal medicine ward for frail older patients.	No	No, a discharge summary was implemented instead.
Implementation points	*3.5*	*2*	*4*	*4*	*3*	*2.5*	*3*	*4.5*
**Education of physicians and nurses**								

For peer review only

How were physicians and nurses informed about the TIP and how often?		Kick-off meeting at participating wards; during morning report; working instructions were sent by email to all physicians		Kick-off meeting; meeting at participating wards; E-learning; 1 feedback meeting		During morning reports; project leader informed every physician separately; intranet; email		During morning reports; intranet; email; posters & pocket cards; and project leaders went to participating wards to inform physicians and nurses		Email and project leader went to participating wards		Kick-off meeting, during several morning reports		Physicians were not educated with regard to the intervention		During several morning report; email; project leaders went to participating wards to inform physicians and nurses; medical specialists from project group informed physicians in person	
Did physicians and/or nurses receive feedback with regard to their discharge letters and if yes, how often?		No		No		No		No		No		Only for nurses		No		Yes, daily on internal medicine and monthly on surgery ward, via email.	
Implementation points		*3*		*4*		*4*		*5*		*2*		*2.5*		*0*		*5*	
**Total implementation points**		**26.5**		**26**		**26**		**34**		**22**		**9**		**5**		**33.5**	
**Pre-intervention vs. post-intervention period scores**																	
**Pre-intervention period**																	
median	% letters within 24h	8.15	9.0	0.90	47.3	6.71	23.5	10.48	13.1	0.79	50.0	6.79	9.2	14.21	7.6	5.83	20.9
**Post-intervention**																	
median	% letters within 24h	9.08	19.5	1.0	48.5	5.48	24.2	5.79	19.7	0.29	49.6	7.98	16.7	22.44	1.5	0.83	53.8

Revised Standards for Quality Improvement Reporting Excellence (SQUIRE 2.0)
September 15, 2015

Text Section and Item Name	Section or Item Description
**Notes to authors**	• The SQUIRE guidelines provide a framework for reporting new knowledge about how to improve healthcare<br>• The SQUIRE guidelines are intended for reports that describe system level work to improve the quality, safety, and value of healthcare, and used methods to establish that observed outcomes were due to the intervention(s).<br>• A range of approaches exists for improving healthcare. SQUIRE may be adapted for reporting any of these.<br>• Authors should consider every SQUIRE item, but it may be inappropriate or unnecessary to include every SQUIRE element in a particular manuscript.<br>• The SQUIRE Glossary contains definitions of many of the key words in SQUIRE.<br>• The Explanation and Elaboration document provides specific examples of well-written SQUIRE items, and an in-depth explanation of each item.<br>• Please cite SQUIRE when it is used to write a manuscript.
**Title and Abstract**	
**1. Title**	Indicate that the manuscript concerns an initiative to improve healthcare (broadly defined to include the quality, safety, effectiveness, patient-centeredness, timeliness, cost, efficiency, and equity of healthcare)
**2. Abstract**	a. Provide adequate information to aid in searching and indexing<br>b. Summarize all key information from various sections of the text using the abstract format of the intended publication or a structured summary such as: background, local problem, methods, interventions, results, conclusions
**Introduction**	*Why did you start?*
**3. Problem Description**	Nature and significance of the local problem
**4. Available knowledge**	Summary of what is currently known about the problem, including relevant previous studies

**5. Rationale**	Informal or formal frameworks, models, concepts, and/or theories used to explain the problem, any reasons or assumptions that were used to develop the intervention(s), and reasons why the intervention(s) was expected to work
**6. Specific aims**	Purpose of the project and of this report
**Methods**	*What did you do?*
**7. Context**	Contextual elements considered important at the outset of introducing the intervention(s)
**8. Intervention(s)**	a. Description of the intervention(s) in sufficient detail that others could reproduce it<br>b. Specifics of the team involved in the work
**9. Study of the Intervention(s)**	a. Approach chosen for assessing the impact of the intervention(s)<br>b. Approach used to establish whether the observed outcomes were due to the intervention(s)
**10. Measures**	a. Measures chosen for studying processes and outcomes of the intervention(s), including rationale for choosing them, their operational definitions, and their validity and reliability<br>b. Description of the approach to the ongoing assessment of contextual elements that contributed to the success, failure, efficiency, and cost<br>c. Methods employed for assessing completeness and accuracy of data
**11. Analysis**	a. Qualitative and quantitative methods used to draw inferences from the data<br>b. Methods for understanding variation within the data, including the effects of time as a variable
**12. Ethical Considerations**	Ethical aspects of implementing and studying the intervention(s) and how they were addressed, including, but not limited to, formal ethics review and potential conflict(s) of interest
**Results**	*What did you find?*
**13. Results**	a. Initial steps of the intervention(s) and their evolution over time (*e.g.*, time-line diagram, flow chart, or table), including modifications made to the intervention during the project<br>b. Details of the process measures and outcome<br>c. Contextual elements that interacted with the intervention(s)<br>d. Observed associations between outcomes, interventions, and relevant contextual elements<br>e. Unintended consequences such as unexpected benefits, problems, failures, or costs associated with the intervention(s).<br>f. Details about missing data
**Discussion**	*What does it mean?*
**14. Summary**	a. Key findings, including relevance to the rationale and specific aims<br>b. Particular strengths of the project

For peer review only

**15. Interpretation**	a. Nature of the association between the intervention(s) and the outcomes<br>b. Comparison of results with findings from other publications<br>c. Impact of the project on people and systems<br>d. Reasons for any differences between observed and anticipated outcomes, including the influence of context<br>e. Costs and strategic trade-offs, including opportunity costs
**16. Limitations**	a. Limits to the generalizability of the work<br>b. Factors that might have limited internal validity such as confounding, bias, or imprecision in the design, methods, measurement, or analysis<br>c. Efforts made to minimize and adjust for limitations
**17. Conclusions**	a. Usefulness of the work<br>b. Sustainability<br>c. Potential for spread to other contexts<br>d. Implications for practice and for further study in the field<br>e. Suggested next steps
**Other information**	
**18. Funding**	Sources of funding that supported this work. Role, if any, of the funding organization in the design, implementation, interpretation, and reporting

For peer review only

Table 2. Glossary of key terms used in SQUIRE 2.0. This Glossary provides the intended meaning of selected words and phrases as they are used in the SQUIRE 2.0 Guidelines. They may, and often do, have different meanings in other disciplines, situations, and settings.

Assumptions
Reasons for choosing the activities and tools used to bring about changes in healthcare services at the system level.

Context
Physical and sociocultural makeup of the local environment (for example, external environmental factors, organizational dynamics, collaboration, resources, leadership, and the like), and the interpretation of these factors ("sense-making") by the healthcare delivery professionals, patients, and caregivers that can affect the effectiveness and generalizability of intervention(s).

Ethical aspects
The value of system-level initiatives relative to their potential for harm, burden, and cost to the stakeholders. Potential harms particularly associated with efforts to improve the quality, safety, and value of healthcare services include opportunity costs, invasion of privacy, and staff distress resulting from disclosure of poor performance.

Generalizability
The likelihood that the intervention(s) in a particular report would produce similar results in other settings, situations, or environments (also referred to as external validity).

Healthcare improvement
Any systematic effort intended to raise the quality, safety, and value of healthcare services, usually done at the system level. We encourage the use of this phrase rather than "quality improvement," which often refers to more narrowly defined approaches.

Inferences
The meaning of findings or data, as interpreted by the stakeholders in healthcare services – improvers, healthcare delivery professionals, and/or patients and families

Initiative
A broad term that can refer to organization-wide programs, narrowly focused projects, or the details of specific interventions (for example, planning, execution, and assessment)

Internal validity
Demonstrable, credible evidence for efficacy (meaningful impact or change) resulting from introduction of a specific intervention into a particular healthcare system.

Intervention(s)
The specific activities and tools introduced into a healthcare system with the aim of changing its performance for the better. Complete description of an intervention includes its inputs, internal activities, and outputs (in the form of a logic model, for example), and the mechanism(s) by which these components are expected to produce changes in a system's performance.

Opportunity costs

For peer review only

Loss of the ability to perform other tasks or meet other responsibilities resulting from the diversion of resources needed to introduce, test, or sustain a particular improvement initiative

Problem
Meaningful disruption, failure, inadequacy, distress, confusion or other dysfunction in a healthcare service delivery system that adversely affects patients, staff, or the system as a whole, or that prevents care from reaching its full potential

Process
The routines and other activities through which healthcare services are delivered

Rationale
Explanation of why particular intervention(s) were chosen and why it was expected to work, be sustainable, and be replicable elsewhere.

Systems
The interrelated structures, people, processes, and activities that together create healthcare services for and with individual patients and populations. For example, systems exist from the personal self-care system of a patient, to the individual provider-patient dyad system, to the microsystem, to the macrosystem, and all the way to the market/social/insurance system. These levels are nested within each other.

Theory or theories
Any "reason-giving" account that asserts causal relationships between variables (causal theory) or that makes sense of an otherwise obscure process or situation (explanatory theory). Theories come in many forms, and serve different purposes in the phases of improvement work. It is important to be explicit and well-founded about any informal and formal theory (or theories) that are used.

For peer review only

BMJ Open

A safe handover for every patient: an interrupted time series analysis to test the effect of a structured discharge bundle in Dutch hospitals

Journal:	*BMJ Open*
Manuscript ID	bmjopen-2018-023446.R3
Article Type:	Research
Date Submitted by the Author:	06-Mar-2019
Complete List of Authors:	van Seben, Rosanne; AMC, Internal Medicine, section of Geriatric Medicine<br>Geerlings, Suzanne; AMC, Internal Medicine, Division of Infectious Diseases<br>Maaskant, Jolanda; AMC, Emma Children's Hospital<br>Buurman, Bianca; Academic Medical Center, Internal Medicine, section of Geriatric Medicine
**Primary Subject Heading**:	Health services research
Secondary Subject Heading:	Communication, Health services research
Keywords:	Discharge Bundle, Patient Handovers, Discharge Letter, Patient Safety, Interrupted Time Series, Quality Improvement

SCHOLARONE™
Manuscripts

A safe handover for every patient: an interrupted time series analysis to test the effect of a structured discharge bundle in Dutch hospitals

Rosanne van Seben (corresponding author)

Amsterdam UMC, University of Amsterdam, Department of Internal Medicine, Section of Geriatric Medicine, Amsterdam Public Health research institute, Meibergdreef 9, Amsterdam, The Netherlands

r.vanseben@amc.uva.nl, +312 56 61647

Suzanne E. Geerlings

Amsterdam UMC, University of Amsterdam, Department of Internal Medicine, Section of Infectious Diseases, Meibergdreef 9, Amsterdam, The Netherlands

Jolanda M. Maaskant

Amsterdam UMC, University of Amsterdam, Emma Children's Hospital, Meibergdreef 9, Amsterdam, The Netherlands

Bianca M. Buurman

Amsterdam UMC, University of Amsterdam, Department of Internal Medicine, Section of Geriatric Medicine, Amsterdam Public Health research institute, Meibergdreef 9, Amsterdam, The Netherlands & ACHIEVE Centre of Expertise, Faculty of Health, Amsterdam University of Applied Sciences, Amsterdam, the Netherlands

On behalf of the TIP study group: Hanneke Pullens, Barbara van Munster, Bianka Mennema, Alie Haze-Visser, Agnes van 't Hof, Mariët Dirkzwager, Stella de Regt, Lisette Bruns, Suzan Vroomen, Jolanda Maaskant, Rosanne van Seben, Suzanne Geerlings and Bianca Buurman

Key words: Discharge Bundle, Patient Handovers, Patient Safety, Interrupted Time Series, Quality Improvement

Word count: 3214

For peer review only - http://bmjopen.bmj.com/site/about/guidelines.xhtml

Abstract

Objective Patient handovers are often delayed, patients are hardly involved in their discharge process and hospital-wide standardized discharge procedures are lacking. The aim of this study was to implement a structured discharge bundle and to test the effect on timeliness of medical and nursing handovers, length of hospital stay (LOS) and unplanned readmissions.

Design Interrupted time series with six pre-intervention and six post-intervention data collection points (September 2015 through June 2017).

Setting Internal medicine and surgical wards.

Participants Patients (≥18 years) admitted for more than 48h to surgical or internal medicine wards.

Intervention The Transfer Intervention Procedure (TIP), containing four elements: planning the discharge date within 48h post-admission, arrangements for postdischarge care, preparing handovers and personalized patient discharge letter; and a discharge conversation 12-24h before discharge.

Outcome measures The number of medical and nursing handovers sent within 24h. Secondary outcomes were median time between discharge and medical handovers, LOS and unplanned readmissions.

Results Pre-intervention 1039 and post-intervention 1052 patient records were reviewed. No significant change was observed in the number of medical and nursing handovers sent within 24h. The median (interquartile range) time between discharge and medical handovers decreased from 6.15 (0.96-15.96) to 4.08 (0.33-13.67) days, but no significant difference was found. No intervention-effect was observed for LOS and readmission. In subgroup analyses, a reduction of 5.6 days in the median time between discharge and medical handovers was observed in hospitals with high protocol adherence and much attention for implementation.

Conclusion Implementation of a structured discharge bundle did not lead to improved timeliness of patient handovers. However, large inter-hospital variation was observed and an intervention effect

For peer review only - http://bmjopen.bmj.com/site/about/guidelines.xhtml

on the median time between discharge and medical handovers was seen in hospitals with high protocol adherence. Future interventions should continue to create awareness of the importance of timely handovers.

Trial Registration: Dutch Trial Registry: NTR5951

Strengths and limitations of this study

- The study design, i.e. interrupted time series analysis, provides a strong quasi-experimental design to evaluate the impact of an intervention aimed at quality improvement.
- The study design, i.e. interrupted time series analysis, provided valuable information on pre-intervention trends, which strengthens the results.
- Sensitivity analysis provided important insight into the inter-hospital variation and differences in intervention effects among hospitals.
- Only the date of sending patient handovers were recorded. Knowing whether the next care provider received information would have been informative.
- It was not possible to evaluate percentages of compliance with the study protocol and the process evaluation with the project leaders might have been an overestimation.

For peer review only

For peer review only - http://bmjopen.bmj.com/site/about/guidelines.xhtml

Introduction

As hospital stays have become shorter and full recovery often takes place at home,[1] a safe transition from hospital to home or nursing home has become more and more important. Besides, a rising number of older chronically ill patients who move within the health care system, requires continuity of care[2 3]. However, transitions from hospital to primary care settings are still considered a high-risk process. Patients are discharged with little coordination or follow-up and are hardly involved in their own discharge process[4 5].

Inadequate transitions may have serious implications for patient safety and quality of care. Postdischarge adverse events such as medication errors, can be the consequence of insufficient or lacking communication between hospital and primary care providers, thereby contributing to higher resource use and unplanned readmission rates[6-11]. In fact, unplanned readmission rates in the first month postdischarge are as high as 20%[12] and a recent study shows that half of them are deemed preventable[11].

The root of a safe transition from hospital to home or nursing home is a timely transfer of the medical handover, that is a letter containing accurate medical discharge information for the next care provider[8 13]. The general practitioner (GP) can only take over responsibility for a patient safely, when receiving a medical handover containing accurate information on, e.g., medications, and follow-up[13]. Nonetheless, a review of Kripalani et al. showed that medical handovers are often not available, lack important information or are not sent in a timely manner[8]. Also, a more recent study performed in 20 Dutch hospitals showed that in 10% of cases medical handover were missing and the remainder was on average sent after one week,[14] even though unplanned readmissions most frequently occur within the first week postdischarge[15].

Previous studies that aimed to improve patient handovers, mainly focused on specific high risk populations and targeted patient-related factors[16-18]. Although such interventions on individualized discharge planning or transitional care have been effective in reducing readmission[16 17]

For peer review only - http://bmjopen.bmj.com/site/about/guidelines.xhtml

and postdischarge mortality rates,[18-20] organizational factors that form the basis of a safe handover should also be optimally arranged[13 21]. In fact, in order to ensure patient safety and continuity of care, early discharge planning, a structured discharge process and timely handovers might be essential[13 21 22]. Besides, given that patients are often unprepared at time of discharge and uncertainties about aspects such as treatment or medication may exist,[5] patient education, e.g., in terms of a proper discharge conversation, should also be an important aspect of the discharge process[6 7].

The aim of this study was, therefore, to implement a structured discharge process, the Transfer Intervention Procedure (TIP), in eight hospitals. The TIP contains four elements: planning the discharge date within 48h after admission, arrangements for required postdischarge care, preparing medical, medication, and nursing handovers and a personalized discharge letter for the patient (PPDL) within 48h after admission; and holding a discharge conversation 12 to 24h before discharge. We tested whether the TIP improved timeliness of medical and nursing handovers and investigated the effect of the TIP procedure on length of hospital stay and unplanned readmissions within 30-days postdischarge.

Methods

Study design and setting

We evaluated the implementation of the TIP discharge bundle in an interrupted time series (ITS), which is the strongest design when a randomized controlled trial is not feasible[23 24]. The trial protocol[25] was based on the recommendations for ITS studies,[23] and we adhered to the SQUIRE guidelines for quality improvement reporting[26]. The current study was part of a large national program, initiated by the Dutch Ministry of Health, Welfare, and Sport (abbreviated in Dutch: VWS): 'Addressing Waste in Health Care'. This program was set up in order to reduce inefficiencies in the provision of health care. As part of this program, a TIP study group was established, comprising a study coordinator, two supervisors, one clinical epidemiologist, a policy officer from the Ministry of

For peer review only - http://bmjopen.bmj.com/site/about/guidelines.xhtml

VWS and local project leaders from the eight participating hospitals (one university and seven regional teaching throughout the Netherlands) that implemented the TIP bundle at one of their surgical and one of their internal medicine wards.

Within an interrupted time series, repeated observations are collected over time and divided into two segments, one before and one after implementation. Therefore, at six pre-intervention data collection points, measurements were conducted before implementation of the TIP and at six post-intervention data collection points measurements were conducted after implementation. During the implementation period of two months no measurements were conducted. February 2016, a kick off meeting was held. Between March 2016 and November 2016, hospitals started with implementation. Data collection started September 2015 and ended June 2017 (Supplement Table 1). All patients (aged ≥18 years) admitted for more than 48h were eligible for inclusion. The Medical Ethics Research Committee (METC) confirmed that the Medical Research Involving Human Subjects Act did not apply to this research project and official approval was not required. Since the study involved a quality improvement intervention with negligible risk of harming patients, individual informed consent was waived for all participating hospitals by the legal department research support of the Amsterdam UMC, location AMC. This trial was registered with the Dutch Trial Registry number NTR5951.

The discharge process in the Netherlands

In the Netherlands, primary care standards are relatively high and basically every person has a general practitioner (GP). When a person is hospitalized, responsibility is taken over from the GP by the medical specialist. After discharge, patient care becomes the responsibility of the GP again. It is policy for hospitals to provide patient handovers to the GP. However, there are no clear guidelines for hospitals how to arrange their discharge process. The Dutch healthcare inspectorate,[27] indicated that standardized discharge processes are lacking and errors that occur during handovers are often resolved informally.

For peer review only - http://bmjopen.bmj.com/site/about/guidelines.xhtml

After discharge from the hospital, the hospital physician sends a medical handover to the primary care provider for every patient (e.g., nursing home physician or the GP). Medical handovers include information on the reason for admission, diagnosis, comorbidity, the course of admission, medical examinations, treatment, medication, the health status of the patient at discharge, and instructions on follow-up[28]. Nursing handovers are only provided when the patient is discharged to a nursing home or with postdischarge care at the patient's own home. Nursing handovers include information on the care provided during hospitalization, current nursing care problems, the reason why (nursing) home care is initiated, and the intended outcomes of the care that will be provided[29].

Intervention

Figure 1 (adapted from van Seben et al.[30]) illustrates how the TIP bundle forms the basis of a safe handover from hospital to primary care for every patient, and if applicable, for patients discharged with postdischarge care (e.g., home care or a nursing home) or for complex patients who require a case manager or transitional care. As described in two previous studies,[25 31] the TIP bundle was developed using input from focus group meetings with professionals, patient surveys and literature. The TIP discharge bundle consists of four elements: 1) planning the discharge date within 48h after admission and communication of the discharge date with the patient, 2) starting with arrangements for required postdischarge care within 48h after admission; 3) preparing patient handovers (medical, medication, nurse) and personalized patient discharge letter (PPDL[32]) within 48h after admission, 4) planning a discharge conversation with the patient to explain information from the PPDL 12-24h before discharge. The PPDL is a standardized document, containing understandable information for the patient on the reason for admission, hospital treatment, course of the disease, possible sustained consequences or complications, and information on medication. We constructed checklists based on the TIP, which served as remembering tool for nurses and physicians in the electronic system or on pocket cards.

For peer review only - http://bmjopen.bmj.com/site/about/guidelines.xhtml

Patient and public Involvement

Our research question was developed from the perspective that patients are discharged with little coordination or follow-up and that they are often unprepared at time of discharge[4 5]. Patients were involved as participants in the construction of the TIP discharge bundle, which was based on, among others, patient satisfaction surveys[25 31]. Further, in a previous study in which the PPDL was developed and implemented, patient satisfaction with the PPDL was also assessed[32].

Protocol adherence

To enhance intervention fidelity and protocol adherence in the different hospitals, regular meetings were held with the TIP study group to report results and provide feedback, to discuss implementation, share experience and learn from each other's practices. A process evaluation was conducted with the project leaders to investigate protocol adherence, implementation strategies and attention paid to implementation. Elements that were considered included leadership and education of project leaders, projects group, extent of implementation of the discharge bundle, and education of physicians and nurses. Feedback points were awarded for all elements and for the extent to which the hospital complied to a certain element, e.g., for every person present at the kick off meeting or for every project meeting that was held. When a hospital partly complied to an element, e.g. automatically generated discharge summaries were provided to the patient instead of a PPDL or feedback on timely handovers was only provided to nurses, 0.5 feedback points were awarded. It was not possible to evaluate percentages of compliance with discharge conversations, planning discharge dates and arrangement of postdischarge care within 48h since these aspects were not reported in patient records. Hospital policies regarding these elements were assessed.

Outcome measures

For peer review only - http://bmjopen.bmj.com/site/about/guidelines.xhtml

Our primary outcome was the number of medical and nursing handovers sent within 24h. This time-frame was based on a report of the Dutch healthcare inspectorate (In Dutch: Inspectie voor de Gezondheidszorg en Jeugd (IGJ)) on the discharge process and handovers, in which it is stated that accurate information needs to be available as quick as possible, but certainly within 24h, for the next care provider[27]. Medical handovers also include medication handovers and we considered the time that these handovers were sent to the GP. The median time between discharge and the medical handover was considered as secondary outcome. Further, secondary outcomes were length of hospital stay (LOS) and rates of unplanned readmission within 30 days.

Baseline data collection

Data regarding patient characteristics included: demographics, admission ward and medical data (i.e. presence of polypharmacy, comorbidity,[33] number of hospitalization in the six months prior to current hospitalization). Variables were all collected from patient files. All data were reported and analyzed anonymously.

Sample size calculation

Based on the findings of a previous study[31] we expected to find a reduction of 78% in the time between discharge and medical handovers sent. We conducted a power analysis with a number of patients based on the number of hospital beds at the participating wards and feasibility with regards to data collection, which was set at 11 patients. In a simulation study with 16 wards, each contributing 65 patients, we estimated the power to be approximate 91% to demonstrate a reduction of 78% in time until sending the medical handover, assuming that the intraclass correlation coefficient does not exceed 0.05.

Statistical analysis

For peer review only - http://bmjopen.bmj.com/site/about/guidelines.xhtml

Descriptive characteristics of patients were calculated using proportions, means and standard deviations (SD), or medians and interquartile ranges (IQR), as appropriate. Chi-squared analysis and the Mann Whitney test were used to compare pre-intervention and post-intervention patient characteristics. Our time series was divided into two segments, one before and one after implementation of the TIP and we used segmented regression analysis to detect post-intervention level changes (i.e., an immediate change in the observed outcome after implementation) and changes in post-intervention trends relative to pre-intervention trends (i.e., a change in slopes of the regression lines after implementation). A least square regression line was fitted to the two segments of the continuous time variable. The segmented regression helped us to estimate the change in the intercept and the slope coefficients between the pre-intervention and post-intervention period using the following model: $Y_t=\alpha+\beta_1\ time_t+\beta_2\ intervention_t+\beta_3\ time\ after\ intervention_t+\varepsilon_t$. Since observations over time are correlated, we explored models with no, a first order autoregressive correlation between consecutive data collection periods, and longer autocorrelation structures.[24] We used the Akaike Information Criterion (AIC) as an estimator of the relative quality of a model and we report the results from the best fitting model. Correction for baseline imbalances as potential confounders led to results with similar estimates and identical interpretation. Based on the extent of protocol adherence and the feedback points awarded, subgroup analyses were performed to assess the intervention effect on the number of medical handovers within 24h and the median time between discharge and medical handovers. Statistical analyses were performed using SPSS Statistics ©, version 24.0, and Rstudio, version 1.0.136 (© 2009 – 2016 Rstudion, Inc).

Results

A total of 2091 patient records (1039 pre- and 1052 post-intervention) were reviewed in order to investigate the effect of the Transfer Intervention Procedure (TIP) on the timeliness of medical and nursing handovers, length of hospital stay (LOS) and unplanned readmission within 30 days. Overall

For peer review only - http://bmjopen.bmj.com/site/about/guidelines.xhtml

patients had a mean age (SD) of 68.1 (16.6) and 46.4% were male (table 1). There were significant differences between the pre-, and post-intervention group with regard to polypharmacy and the ratio of acute/elective hospitalizations and these variables were considered as potential confounders. However, correction for these potential confounders did not provide better models than the presented models.

Protocol adherence

Implementation strategies and protocol adherence are summarized in Supplement Table 1. Based on the process evaluation, three subgroups were identified. Subgroup 1 (hospitals 4 and 8), >30 feedback points, paid considerable attention to implementation and there was relatively high protocol adherence. In subgroup 2 (hospitals 1-3, and 5), 20-30 feedback points, there was relatively high protocol adherence but moderate attention to implementation. In subgroup 3 (hospitals 6 and 7), <10 feedback points, nearly no attention was brought to implementation and there was low compliance.

Medical and nursing handovers

In the total study population, no intervention effect was found on the percentage of medical handovers being sent within 24h after hospital discharge to the GP: 22.7% medical handovers were sent within 24h pre-intervention, 29.1% post-intervention and no significant difference was observed in the levels and trends between the pre-intervention and post-intervention period. The median (interquartile range, IQR) time between discharge and medical handovers decreased from 6.15 (0.96-15.96) days, pre-intervention to 4.08 (0.33-13.67) days post-intervention. An absolute effect directly after the implementation of the intervention of -0.25 days was found (i.e., de difference in time between discharge and medical handovers between the sixth pre-intervention data collection point and first post-intervention data collection point). We observed no significant difference in the levels

For peer review only - http://bmjopen.bmj.com/site/about/guidelines.xhtml

and trends. The number of nursing handovers sent within 24h postdischarge was 92.8% pre-intervention and 93.1% post-intervention and no significant difference was observed between levels and trends. The results are presented in Figure 2 and the parameters estimates are summarized in Table 2.

Length of hospital stay and unplanned readmission rates

No significant decline in the levels and trends between the pre-, and post-intervention was found with regard to LOS (β 0.08, 95% CI -0.12 to 0.29 p=0.45) and unplanned readmission rates (β 1.11, 95% CI -2.55 to 0.33 p=0.17). Median (IQR) LOS was 8.17 (4.75-15.13) and 8.56 (4.88-15.91) days and readmissions rates were as high as 11.1% and 12.3% pre-intervention and post-intervention, respectively. With regard to LOS, the results are adjusted for autocorrelation (AIC 22.64 versus 33.75, p=0.01), but not for potential confounders (AIC 43.08 versus 33.75, p=0.07). With regard to unplanned readmission rates, the results are unadjusted for autocorrelation (AIC 57.18 versus 54.45, p=0.10) and potential confounders (AIC 57.47 versus 54.45, p=0.61).

Subgroup analysis

In subgroup 1 (>30 feedback points), an absolute effect of 13.3% more medical handovers sent within 24h postdischarge was observed but this did not result in significant changes in level or trends (Figure 3). A reduction of 5.6 days in the median time between discharge and handovers with a significant change in level directly after the intervention was observed in subgroup 1 (β -5.29, 95% CI -8.70 to 1.87 p=0.02). Pre-intervention, group 2 (20-30 feedback points) had the highest rate of medical handovers sent within 24h and the lowest median time between discharge and medical handovers but no intervention effect was observed. Both pre- and post-intervention, subgroup 3 (<10 points) had the lowest rates of medical handovers sent within 24h, and the highest median time. We observed no intervention effect in subgroup 3.

For peer review only - http://bmjopen.bmj.com/site/about/guidelines.xhtml

Discussion

In the total study population, a structured discharge bundle, the Transfer Intervention Procedure (TIP), did not lead to improved timeliness of medical and nursing handovers. Although medical handovers were sent faster post-intervention (pre-intervention median 6.15; post-intervention 4.08 days), we were unable to show significant differences in level and trends, both with regard to the median time and the number of medical handovers sent within 24h. However, large inter-hospital variation was observed and a significant intervention effect on the median time between discharge and medical handovers was seen in those hospitals with relatively high protocol adherence and attention for implementation. Rates of nursing handovers sent within 24h were both pre- and post-intervention above 90%. No intervention effect was found for length of hospital stay (LOS) and readmissions.

Extensive research has been conducted to improve patient handovers from hospital to home[7 16]. Summarizing findings of earlier discharge interventions that aimed to improve coordination of care and communication between hospital and primary care providers, Hesselink et al.,[7] and Kripalani et al.,[8] showed that some studies were able to improve timeliness of medical handovers. These interventions, however, were based on the introduction of fax, email or web-based transfers of information, which is increasingly becoming standard practice in Dutch hospitals. Yet, further improvement may lie in electronic sending systems that support the use of standardized formats that pull information from patient files into (medical) handovers or that send information to the next care provider automatically.

Although a before-after design would probably have led to a significant intervention-effect, the ITS analysis provided valuable information on pre-intervention trends. The observed median time between discharge and sending medical handovers at our first pre-intervention measurement point was consistent with a recent Dutch study[14], but a trend towards sending handovers faster was

For peer review only - http://bmjopen.bmj.com/site/about/guidelines.xhtml

already observed along the pre-intervention period. During the pre-intervention period, no interventions were implemented and the TIP was introduced and implemented during a two-month implementation period during which no measurements were conducted. However, in the pre-intervention period, attention was already brought to the discharge process, e.g. by establishing project groups and the kick-off meeting. Although these activities were not intended as implementation strategies, in hindsight they might explain why improvements were already observed during the pre-intervention period, particularly since education on the importance of the intervention is an important aspect of implementation[13 34 35].

Although positive trends in the pre-intervention period were less pronounced in the subgroup analysis, results of the separate analyses support the idea that attention is important. Whereas a significant reduction of six days in the median time between discharge and medical handovers was observed in hospitals that paid much attention to implementation, no intervention effect was observed in hospitals that paid moderate to nearly no attention. It should be noted that the hospitals that paid moderate attention had relatively good pre-intervention scores. A smaller window for improvement in these hospitals might also explain a lack of intervention effect[36].

Implementation of the TIP procedure did not reveal a reduction of LOS. Although a possible explanation can be low overall compliance with our study protocol, it is also plausible that over the past years, average LOS has decreased to a minimum[37]. Given current pressure on availability of hospital beds, patients are discharged as soon as possible. This may account for inadequate discharge processes, since physicians are forced to prioritize acute health care over discharge-related tasks[38 39].

Given increasingly shorter LOS[37] and the often complex care needs patient face, patient preparation should be an important aspect of the discharge process. In fact, the most effective discharge interventions seem to have educational components[40]. Unfortunately, given the workload among residents, implementation of a personalized patient discharge letter was unsuccessful. E.g., posing the question “do you feel ready to go home”[41] or postdischarge telephone contact,[7] might be

For peer review only - http://bmjopen.bmj.com/site/about/guidelines.xhtml

less time-consuming ways to involve patients. However, to prevent readmissions more effort might be necessary. Previous interventions that revealed a reduction in readmission rates, consist of individualized discharge planning or continue postdischarge[16 42]. However, we believe that a structured discharge process such as the TIP should form the basis for a safe handover for every patient (Figure 1).

Implications for further research

Our study shed light on the difficulties that come along with implementation of quality improvement collaboratives[43]. Given the positive pre-intervention trends and significant reduction in the median time between discharge and medical handovers in hospitals that paid much attention to implementation, further improvements may lie in interventions that create more awareness of the importance of timely handovers and hospital physicians' crucial role in the provision of continuity of care. This might stimulate physicians' intrinsic motivation to provide a structured discharge process and thereby timely handovers[7 39]. Furthermore, we might also want to focus on local factors that lead to insufficient discharge processes. A comprehensive exploration of local barriers for each step in the TIP discharge process might be helpful in order to develop tailor made interventions on a local or department level to improve the discharge process[44].

Limitations

An interrupted time series provides a strong quasi-experimental design to evaluate the impact of an intervention aimed at quality improvement. However, this study design also has limitations. First of all, a positive trend towards sending handovers faster along the pre-intervention period, which was probably due to the attention that was already brought to the discharge process before implementation of the discharge bundle. In fact, an important limitation of ITS is that it is more difficult to determine whether the observed effect is a direct effect of the intervention, in contrast to

For peer review only - http://bmjopen.bmj.com/site/about/guidelines.xhtml

e.g., clustered trials. Second, medical staff was not blinded for the outcome measure, that is timely discharge letters. Knowing that timeliness of discharge letters was monitored might have altered our results. However, in most hospitals timeliness of discharge letters was already monitored before we started with our research project and the effect is likely to be minimal. Third, we only recorded the date of sending medical handovers. Knowing whether they were received by GPs would also have provided valuable information. Fourth, we did not look at the content of handovers, while this might have given us important insights. Lastly, it was not possible to evaluate percentages of protocol adherence and the process evaluation with the project leaders might have been an overestimation. However, the process evaluation was in line with the efforts observed during implementation.

Conclusion

Implementation of a structured discharge bundle, the Transfer Intervention Procedure (TIP), did not lead to more medical and nursing handovers sent within 24h postdischarge. Large inter-hospital variation was observed however, and a significant intervention effect on the median time between discharge and medical handovers was seen in those hospitals with high protocol adherence and that brought much attention to implementation. We believe that future interventions should continue to create awareness of the importance of timely handovers and we hope that our study contributes to this, stimulating hospitals to further structure and improve their discharge process.

Contributors: BB, SG, and RVS designed the study. BB and SG, conceived the study and obtained funding. RVS collected data and JM and RVS performed statistical analysis. All authors contributed to drafting the manuscript or revised it critically and gave final approval for publication. The authors would like to acknowledge the contribution of the TIP study group, consisting in addition to the authors, of the following members Hanneke Pullens (Catharina Hospital Eindhoven), Barbara van Munster (Gelre Hospitals Apeldoorn), Annemarie van der Lugt (Haven Hospital Rotterdam), Alie

For peer review only - http://bmjopen.bmj.com/site/about/guidelines.xhtml

Haze-Visser (Lange Land Hospital Zoetermeer), Agnes van ‘t Hof (Maxima Medical Center Veldhoven), Mariët Dirkzwager (OLVG Amsterdam), Stella de Regt (Reinier de Graaf Hospital Delft), Suzan Vroomen (Amsterdam UMC, location AMC) and Lisette Bruns (Dutch Ministry of Health, Welfare, and Sport). Furthermore, we thank Rachel de Vries en Vera van Miltenburg for their assistance with data collection.

Funding sources: This work was supported by the Dutch Ministry of Health, Welfare and Sport [grant number: 324798].

Conflicts of interest: None declared.

Data sharing statement: All data relevant to the study are included in the article or uploaded as supplementary information.

For peer review only

For peer review only - http://bmjopen.bmj.com/site/about/guidelines.xhtml

References

1. Eurostat Statistics Explained. Hospital discharges and length of stay statistics 2016 [Available from: http://ec.europa.eu/eurostat/statistics-explained/index.php/Hospital_discharges_and_length_of_stay_statistics accessed October 17 2017.
2. Jean Hall M, DeFrances CJ, Williams SN, Golonsinskiy MS, Schwartzman A. National Health Statistics Reports. *US Department of Health and Guman Services Centers for Disease Control and Prevention National Center for Health Statistics* 2010;29
3. Boyd CM, Darer J, Boult C, Fried LP, Boult L, Wu AW. Clinical practice guidelines and quality of care for older patients with multiple comorbid diseases: implications for pay for performance. *Jama* 2005;294(6):716-24. doi: 10.1001/jama.294.6.716 [published Online First: 2005/08/11]
4. Henderson A, Zernike W. A study of the impact of discharge information for surgical patients. *Journal of advanced nursing* 2001;35(3):435-41. [published Online First: 2001/08/08]
5. Holland DE, Mistiaen P, Bowles KH. Problems and unmet needs of patients discharged "home to self-care". *Professional case management* 2011;16(5):240-50; quiz 51-2. doi: 10.1097/NCM.0b013e31822361d8 [published Online First: 2011/08/19]
6. Greenwald JL, Jack BW. Preventing the preventable: reducing rehospitalizations through coordinated, patient-centered discharge processes. *Professional case management* 2009;14(3):135-40; quiz 41-2. doi: 10.1097/NCM.0b013e318198d4e1 [published Online First: 2009/05/29]
7. Hesselink G, Schoonhoven L, Barach P, et al. Improving patient handovers from hospital to primary care: a systematic review. *Annals of internal medicine* 2012;157(6):417-28. doi: 10.7326/0003-4819-157-6-201209180-00006 [published Online First: 2012/09/19]
8. Kripalani S, LeFevre F, Phillips CO, Williams MV, Basaviah P, Baker DW. Deficits in communication and information transfer between hospital-based and primary care physicians: implications for patient safety and continuity of care. *Jama* 2007;297(8):831-41. doi: 10.1001/jama.297.8.831 [published Online First: 2007/03/01]
9. Forster AJ, Murff HJ, Peterson JF, Gandhi TK, Bates DW. The incidence and severity of adverse events affecting patients after discharge from the hospital. *Annals of internal medicine* 2003;138(3):161-7. [published Online First: 2003/02/01]
10. Moore C, Wisnivesky J, Williams S, McGinn T. Medical errors related to discontinuity of care from an inpatient to an outpatient setting. *Journal of general internal medicine* 2003;18(8):646-51. [published Online First: 2003/08/13]
11. Fluitman KS, van Galen LS, Merten H, et al. Exploring the preventable causes of unplanned readmissions using root cause analysis: Coordination of care is the weakest link. *European journal of internal medicine* 2016;30:18-24. doi: 10.1016/j.ejim.2015.12.021 [published Online First: 2016/01/18]
12. Kansagara D, Englander H, Salanitro A, et al. Risk prediction models for hospital readmission: a systematic review. *Jama* 2011;306(15):1688-98. doi: 10.1001/jama.2011.1515 [published Online First: 2011/10/20]
13. Merten H, van Galen LS, Wagner C. Safe handover. *BMJ (Clinical research ed)* 2017;359:j4328. doi: 10.1136/bmj.j4328 [published Online First: 2017/10/11]
14. Langelaan M, Baines RJ, de Bruijne MC, Wagner C. Association of admission and patient characteristics with quality of discharge letters: posthoc analysis of a retrospective study. *BMC health services research* 2017;17(1):225. doi: 10.1186/s12913-017-2149-8 [published Online First: 2017/03/23]
15. Dharmarajan K, Hsieh AF, Lin Z, et al. Diagnoses and timing of 30-day readmissions after hospitalization for heart failure, acute myocardial infarction, or pneumonia. *Jama* 2013;309(4):355-63. doi: 10.1001/jama.2012.216476 [published Online First: 2013/01/24]

For peer review only - http://bmjopen.bmj.com/site/about/guidelines.xhtml

16. Verhaegh KJ, MacNeil-Vroomen JL, Eslami S, Geerlings SE, de Rooij SE, Buurman BM. Transitional care interventions prevent hospital readmissions for adults with chronic illnesses. *Health affairs (Project Hope)* 2014;33(9):1531-9. doi: 10.1377/hlthaff.2014.0160 [published Online First: 2014/09/10]
17. Prvu Bettger J, Alexander KP, Dolor RJ, et al. Transitional care after hospitalization for acute stroke or myocardial infarction: a systematic review. *Annals of internal medicine* 2012;157(6):407-16. doi: 10.7326/0003-4819-157-6-201209180-00004 [published Online First: 2012/09/19]
18. Shepperd S, Lannin NA, Clemson LM, McCluskey A, Cameron ID, Barras SL. Discharge planning from hospital to home. *The Cochrane database of systematic reviews* 2013;1:Cd000313. doi: 10.1002/14651858.CD000313.pub4 [published Online First: 2013/02/27]
19. Buurman BM, Parlevliet JL, Allore HG, et al. Comprehensive Geriatric Assessment and Transitional Care in Acutely Hospitalized Patients: The Transitional Care Bridge Randomized Clinical Trial. *JAMA internal medicine* 2016;176(3):302-9. doi: 10.1001/jamainternmed.2015.8042 [published Online First: 2016/02/18]
20. Naylor MD, Brooten DA, Campbell RL, Maislin G, McCauley KM, Schwartz JS. Transitional care of older adults hospitalized with heart failure: a randomized, controlled trial. *Journal of the American Geriatrics Society* 2004;52(5):675-84. doi: 10.1111/j.1532-5415.2004.52202.x [published Online First: 2004/04/17]
21. The Joint Commission. Hot Topics in Health Care. Transitions of Care: The need for a more effective approach to continuing patient care. *The Joint Commission* 2012
22. Kripalani S, Jackson AT, Schnipper JL, Coleman EA. Promoting effective transitions of care at hospital discharge: a review of key issues for hospitalists. *Journal of hospital medicine* 2007;2(5):314-23. doi: 10.1002/jhm.228 [published Online First: 2007/10/16]
23. Jandoc R, Burden AM, Mamdani M, Levesque LE, Cadarette SM. Interrupted time series analysis in drug utilization research is increasing: systematic review and recommendations. *Journal of clinical epidemiology* 2015;68(8):950-6. doi: 10.1016/j.jclinepi.2014.12.018 [published Online First: 2015/04/22]
24. Penfold RB, Zhang F. Use of interrupted time series analysis in evaluating health care quality improvements. *Academic pediatrics* 2013;13(6 Suppl):S38-44. doi: 10.1016/j.acap.2013.08.002 [published Online First: 2013/12/07]
25. van Seben R, Geerlings SE, Verhaegh KJ, Hilders CG, Buurman BM. Implementation of a Transfer Intervention Procedure (TIP) to improve handovers from hospital to home: interrupted time series analysis. *BMC health services research* 2016;16:479. doi: 10.1186/s12913-016-1730-x [published Online First: 2016/09/09]
26. Davidoff F, Batalden P, Stevens D, Ogrinc G, Mooney SE. Publication guidelines for quality improvement studies in health care: evolution of the SQUIRE project. *BMJ (Clinical research ed)* 2009;338:a3152. doi: 10.1136/bmj.a3152 [published Online First: 2009/01/21]
27. Dutch Health Care Inspectorate (IGZ). Continuïteit van zorg voor kwetsbare ouderen vanuit het ziekenhuis naar verpleeg- en verzorgingshuizen, thuiszorg en huisartsen niet gewaarborgd. . 2015
28. Joint Commission on Accreditation of Healthcare Organizations. Accreditation Standards for Hospitals. Standard ACC.3 Oakbrook Terrace, IL: The Joint Commission 2010.
29. V&VN. Richtlijn Verpleegkundige en verzorgende verslaglegging September 2011 [
30. van Seben R, Geerlings SE, Buurman BM. [10 tips for improving patient handovers from hospital to other health care settings]. *Ned Tijdschr Geneeskd* 2017;161(0):D1031. [published Online First: 2017/04/27]
31. Verhaegh KJ, Buurman BM, Veenboer GC, de Rooij SE, Geerlings SE. The implementation of a comprehensive discharge bundle to improve the discharge process: a quasi-experimental

For peer review only - http://bmjopen.bmj.com/site/about/guidelines.xhtml

study. *The Netherlands journal of medicine* 2014;72(6):318-25. [published Online First: 2014/10/17]
32. Buurman BM, Verhaegh KJ, Smeulers M, et al. Improving handoff communication from hospital to home: the development, implementation and evaluation of a personalized patient discharge letter. *International journal for quality in health care : journal of the International Society for Quality in Health Care / ISQua* 2016;28(3):384-90. doi: 10.1093/intqhc/mzw046 [published Online First: 2016/05/27]
33. Charlson ME, Pompei P, Ales KL, MacKenzie CR. A new method of classifying prognostic comorbidity in longitudinal studies: development and validation. *Journal of chronic diseases* 1987;40(5):373-83. [published Online First: 1987/01/01]
34. Michie S, van Stralen MM, West R. The behaviour change wheel: a new method for characterising and designing behaviour change interventions. *Implementation science : IS* 2011;6:42. doi: 10.1186/1748-5908-6-42 [published Online First: 2011/04/26]
35. Spoorenberg V, Geerlings SE, Geskus RB, de Reijke TM, Prins JM, Hulscher ME. Appropriate antibiotic use for patients with complicated urinary tract infections in 38 Dutch Hospital Departments: a retrospective study of variation and determinants. *BMC infectious diseases* 2015;15:505. doi: 10.1186/s12879-015-1257-5 [published Online First: 2015/11/11]
36. Ivers N, Jamtvedt G, Flottorp S, et al. Audit and feedback: effects on professional practice and healthcare outcomes. *The Cochrane database of systematic reviews* 2012(6):Cd000259. doi: 10.1002/14651858.CD000259.pub3 [published Online First: 2012/06/15]
37. Coppa Consultancy. Ligduurmonitor Nederlandse ziekenhuizen 2013 & 2014. Trendbreuk in de ligduur. 2015 [Available from: http://www.coppa.nl/wp-content/uploads/2016/01/Ligduurmonitor-2013-2014.pdf accessed November, 2017.
38. Greysen SR, Schiliro D, Horwitz LI, Curry L, Bradley EH. "Out of sight, out of mind": housestaff perceptions of quality-limiting factors in discharge care at teaching hospitals. *Journal of hospital medicine* 2012;7(5):376-81. doi: 10.1002/jhm.1928 [published Online First: 2012/03/02]
39. Hesselink G, Vernooij-Dassen M, Pijnenborg L, et al. Organizational culture: an important context for addressing and improving hospital to community patient discharge. *Medical care* 2013;51(1):90-8. doi: 10.1097/MLR.0b013e31827632ec [published Online First: 2012/11/08]
40. Mistiaen P, Francke AL, Poot E. Interventions aimed at reducing problems in adult patients discharged from hospital to home: a systematic meta-review. *BMC health services research* 2007;7:47. doi: 10.1186/1472-6963-7-47 [published Online First: 2007/04/06]
41. van Galen LS, Brabrand M, Cooksley T, et al. Patients' and providers' perceptions of the preventability of hospital readmission: a prospective, observational study in four European countries. *BMJ quality & safety* 2017 doi: 10.1136/bmjqs-2017-006645 [published Online First: 2017/06/24]
42. Leppin AL, Gionfriddo MR, Kessler M, et al. Preventing 30-day hospital readmissions: a systematic review and meta-analysis of randomized trials. *JAMA internal medicine* 2014;174(7):1095-107. doi: 10.1001/jamainternmed.2014.1608 [published Online First: 2014/05/14]
43. Hulscher ME, Schouten LM, Grol RP, Buchan H. Determinants of success of quality improvement collaboratives: what does the literature show? *BMJ quality & safety* 2013;22(1):19-31. doi: 10.1136/bmjqs-2011-000651 [published Online First: 2012/08/11]
44. Flottorp SA, Oxman AD, Krause J, et al. A checklist for identifying determinants of practice: a systematic review and synthesis of frameworks and taxonomies of factors that prevent or enable improvements in healthcare professional practice. *Implementation science : IS* 2013;8:35. doi: 10.1186/1748-5908-8-35 [published Online First: 2013/03/26]

For peer review only - http://bmjopen.bmj.com/site/about/guidelines.xhtml

Table 1. Baseline characteristics

**Variable**	**Overall (N=2091)**	**Pre-intervention (N=1039)**	**Post-intervention (N=1052)**
**Age in years,** mean (SD)[a]	68.07 (16.57)	67.66 (16.70)	68.48 (16.45)
**Male,** No. (%)	971 (46.4)	493 (47.4)	478 (45.4)
**Living arrangements before admission,** No.(%)			
Independent	1814 (86.7)	883 (84.9)	931 (88.5)
Nursing home	49 (2.3)	27 (2.6)	22 (2.1)
Senior residence/Assisted living	168 (8.1)	91 (8.8)	77 (7.3)
Missing	60 (2.9)	38 (3.7)	22 (2.1)
**Marital status,** No. **(%)**			
Married or living together	1125 (53.8)	556 (53.5)	569 (54.1)
Single or divorced	456 (21.8)	212 (20.4)	244 (23.2)
Widow/widower	435 (20.8)	224 (21.6)	211 (20.1)
Missing	75 (3.6)	47 (4.5)	28 (2.7)
**Charlson Comorbidity Index** [b] (mean, SD [a])	2.05 (2.05)	2.10 (2.08)	2.01 (2.03)
**Polypharmacy,** No. (%)[c, d, e]	1247 (59.6)	586 (56.4)	661 (62.8)
Missing	12 (.6)	8 (.8)	4 (.4)
**Hospitalization in past 6 months,** No. (%)	705 (33.7)	339 (32.6)	336 (34.8)
**Acute hospitalization,** No. (%)[c, f]	73.0 (73.0)	725 (69.8)	801 (76.1)
**Admission ward, internal medicine** No. (%)	1051 (50.3)	524 (50.4)	527 (50.1)
**Discharge destination,** No. (%)			
Home	1551 (74.2)	770 (74.1)	781 (74.2)
Other health care setting, of which	482 (23.1)	238 (23.0)	244 (23.2)
Rehabilitation center	268 (12.8)	120 (11.5)	148 (14.1)
Nursing home	158 (7.6)	80 (7.7)	78 (7.4)
Assisted living	34 (1.6)	26 (2.5)	8 (0.8)
Other hospital	22 (1.1)	12 (1.2)	10 (1.0)
Missing	58 (2.8)	31 (3.0)	27 (2.6)

[a] Standard Deviation, [b] Range of 0 to 31, with a higher score indicating more or more severe comorbidity[33], [c] Use of 5 or more different medications, [d] Chi-Square, [e] P-value = 0.004, [f] P-value = 0.001

For peer review only

For peer review only - http://bmjopen.bmj.com/site/about/guidelines.xhtml

Table 2. Interrupted time series analysis; medical and nursing handovers

	Medical handovers <24 hrs after discharge (%) [a]			***Time between discharge and medical letter (days)*** [b]			***Nursing handovers <24 hrs after discharge (%)*** [c]		
	**β (SE)**	**95% CI**	**p-value**	**β (SE)**	**95% CI**	**p-value**	**β (SE)**	**95% CI**	**p-value**
Intercept	17.51 (3.79)	10.08 to 24.93	<0.01	7.20 (0.29)	6.63 to 7.76	<0.01	91.85 (2.71)	86.53 to 97.16	<0.01
Trend pre-intervention (β1)	1.49 (0.97)	-0.42 to 3.40	0.16	-0.30 (0.07)	-0.45 to -0.16	<0.01	0.28 (0.70)	-1.09 to 1.64	0.70
Level change directly after intervention (β2)	6.43 (10.13)	-13.43 to 26.28	0.54	-0.62 (0.74)	-2.07 to 0.84	0.43	6.32 (7.25)	-7.89 to 20.53	0.41
Trend differences (β3)	-0.94 (1.38)	-3.64 to 1.75	0.51	0.05 (0.10)	-0.14 to 0.25	0.61	-0.81 (0.99)	-2.74 to 1.12	0.43
	Absolute effect directly after intervention: -0.17%			Absolute effect directly after intervention: -0.25 days			Absolute effect directly after intervention: 0.62%		

β1 estimates the pre-intervention trend.

β2 estimates the difference between the observed level just after the intervention started and that predicted by the pre-intervention trend.

β3 estimates the difference in trend between the pre-intervention and post-intervention period.

SE: Standard Error, CI: Confidence Interval

[a] Correction for autocorrelation did not provide a better model compared to the presented model (AIC 74.17 versus 72.88, p=0.40), nor did correction for potential confounders ('polypharmacy' and 'acute admission') (AIC 74.98 versus 72.88, p=0.39). All models led to results with similar estimates and identical interpretation.

[b] The results are adjusted for autocorrelation, but not for potential confounders. Correction for autocorrelation (AR1) provided a better model compared to the presented model (AIC 21.52 versus 25.72, p=0.01). Correction for potential confounders ('polypharmacy' and 'acute admission') did not provide a better model compared to the presented model (AIC 29.23 versus 25.72, p=0.78). Correction for autocorrelation (AR1) changed β1 into a significant result. Correction for potential confounders did not alter the results.

[c] Correction for autocorrelation did not provide a better model compared to the presented model (AIC 66.05 versus 59.13, p=0.02), nor did correction for potential confounders ('polypharmacy' and 'acute admission') (AIC 59.03 versus 59.13, p=0.13). All models led to results with similar estimates and identical interpretation.

For peer review only - http://bmjopen.bmj.com/site/about/guidelines.xhtml

Figure 1. Pyramid for postdischarge care

A structured discharge process such as the TIP procedure should form the basis for every patient. For patients discharged with postdischarge care (20-25%), nursing handovers should be set up within 48h after admission and be sent within 24h postdischarge. Complex patients with a high readmission risk (10%) require a (nurse) case manager or transitional care in the transition from hospital to home.

Figure 2.

Panel A The number of medical handovers sent within 24 hours.

Panel B median time in days between discharge and the medical handovers.

Figure 3. Hospital differences based on implementation score.

The inter-hospital differences in rates of medical handovers being sent within 24h in the pre- and post-intervention based on the extent of implementation and used implementation strategies. Group 1 received >30 feedback implementation points, group 2 received 20-30 implementation points, group 3 received <20 points.

For peer review only

For peer review only - http://bmjopen.bmj.com/site/about/guidelines.xhtml

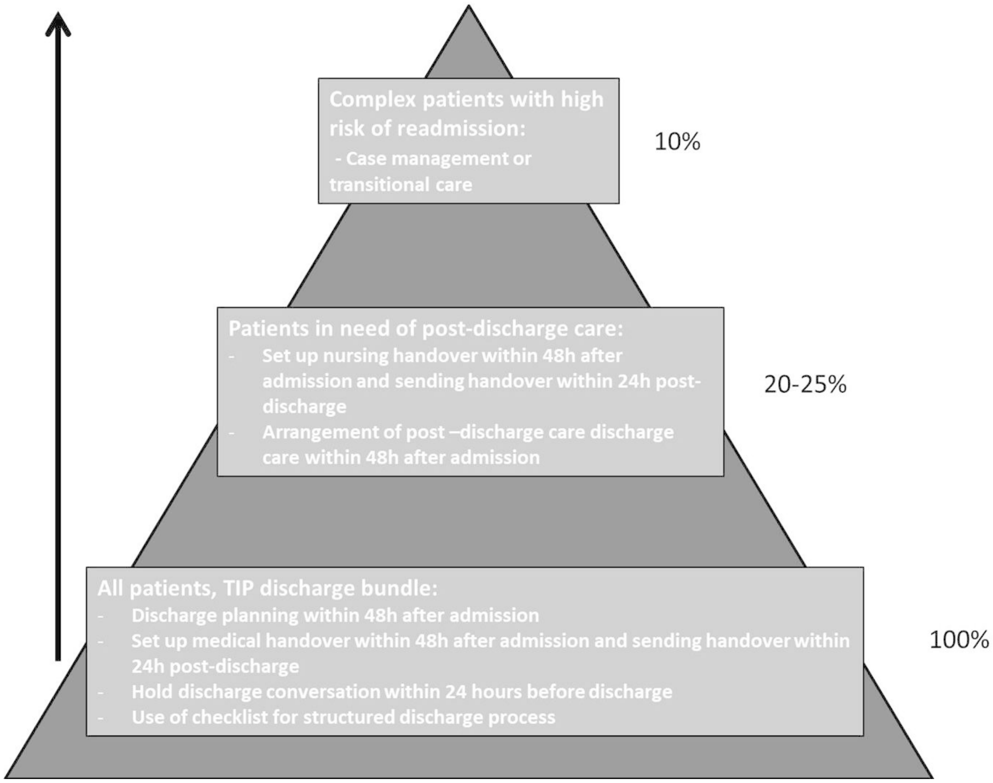

Figure 1. Pyramid for post-discharge care A structured discharge process such as the TIP procedure should form the basis for every patient. For patients discharged with post-discharge care (20-25%), nursing handovers should be set up within 48h after admission and be sent within 24h post-discharge. Complex patients with a high readmission risk (10%) require a (nurse) case manager or transitional care in the transition from hospital to home.

116x92mm (300 x 300 DPI)

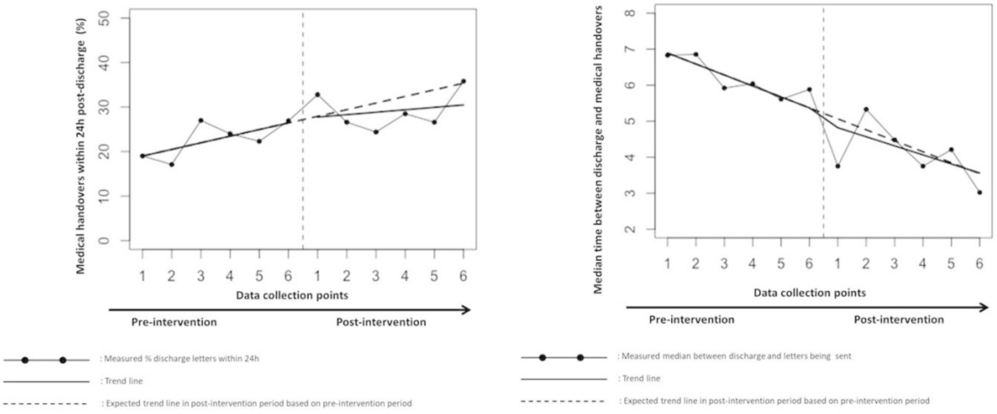

Figure 2.
Panel A The number of medical handovers sent within 24 hours.
Panel B median time in days between discharge and the medical handovers.

113x50mm (300 x 300 DPI)

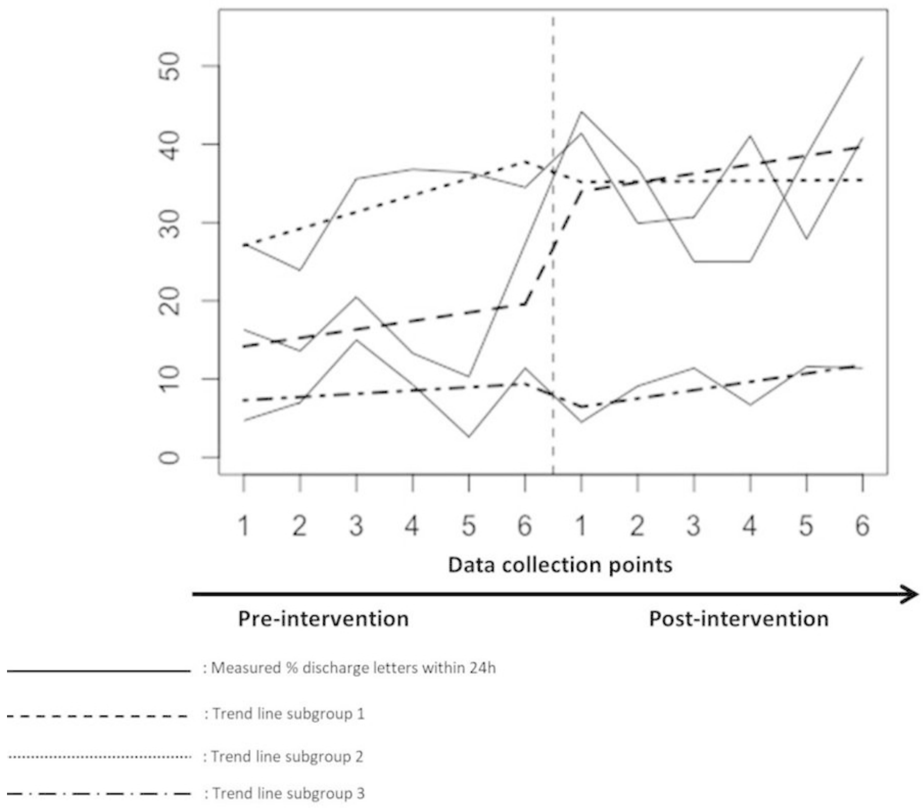

Figure 3. Hospital differences based on implementation score.
The inter-hospital differences in rates of medical discharge letters being sent within 24h in the pre- and post-intervention based on the extent of implementation and used implementation strategies. Group 1 received >30 feedback implementation points, group 2 received 20-30 implementation points, group 3 received <20 points.

82x69mm (300 x 300 DPI)

Supplement Table 1. Adherence to the Intervention Protocol

	Hospital 1	Hospital 2	Hospital 3	Hospital 4	Hospital 5	Hospital 6	Hospital 7	Hospital 8
Pre-intervention<br>Implementation<br>Post-intervention	Sep '15 - Feb '16<br>March '16 - April '16<br>May '16 – Oct '16	Oct '15 - March '16<br>April '16 - May '16<br>June '16 - Nov '16	Jan '16 - June '16<br>July '16 - Aug '16<br>Sep '16 – Feb '17	Dec '15 - May '16<br>June '16 - July '16<br>Aug '16 - Jan '16	March '16 – Aug '16<br>Sep '16 - Oct '16<br>Nov '16 – April '17	April '16 – Sep '16<br>Oct '16 - Nov '16<br>Dec '16 – May '17	May '16 – Oct '16<br>Nov '16 - Dec '16<br>Jan '17 – June '17	April '16 – Sep '16<br>Oct '16 - Nov '16<br>Dec '16 – May '17
**Leadership and education of project leaders**								
Who were present at the kick off meeting February, 2016?	Hospital president; local project leader; 2 team leaders (nurses); 2 physicians; nurse; pharmacists	Local project leader; geriatrician; head of the liaison department; physician	2 local project leaders; head of the liaison department	Chief of staff; local project leader; team leader surgery ward (nurse); head of the liaison department	Local project leader; head of the liaison department; manager patient logistics; 2 team leaders (nurses)	2 local project leaders	Local project leader	Local project leader; senior researcher transitional care; medical specialist,
Who were present at the first feedback session?	Project leader; head of the liaison department	Project leader; pharmacist; communication assistant	2 local project leaders; liaison nurse	Project leader; liaison nurse; nurse geriatrics	Local project leader	2 local project leaders	-	2 project leaders
Who were present at the second feedback session?	Project leader	Project leader	2 project leaders	-	Project leader	-	Project leader	2 project leaders
Implementation points	*10*	*8*	*8*	*7*	*7*	*4*	*2*	*7*
**Project group**								
Was there a local TIP project group, and who participated?	Yes, project leader; 2 senior nurses of participating wards, management assistant	Yes, project leader; geriatrician; head liaison department, physician; pharmacist; communication assistant; manager Security & Services	Yes, 2 project leaders; 2 residents; 2 medical specialists; nurse; liaison nurse; pharmacist; manager	Yes, chief of staff; project leader; 2 team leaders (nurses); head liaison department; orthopedist	Yes, project leader; head liaison department; 2 medical specialists, geriatrician	No	No	Yes, local project leader; 3 medical specialists; 2 residents, manager quality and safety; manager process optimization; medical director
How often did the local project group meet?<br>1 point per meeting	Monthly for 2 months, during pilot period every week (2 months).	Every five weeks during pre-intervention and pilot period	2 times, before pilot period.	Every two weeks during pre-intervention period	Every six weeks, during pre-intervention and pilot period	-	-	Monthly during pre-intervention, pilot period and first two months of post-

For peer review only

								intervention period
Implementation points	*10*	*12*	*10*	*18*	*10*	*0*	*0*	*17*
**Implementation of TIP elements**								
Was it policy to set a discharge date within 48h after admission?	Yes	Yes	Yes	Yes	Yes	Yes	Yes	Yes
Was the planned discharge date communicated to patients?	Yes	Yes	Yes	Yes	Yes	No	Yes	Yes
Was it policy to start with arrangement of required post-discharge care within 48h after admission?	No, liaison nurse has to wait for final discharge date	No, liaison nurse has to wait for final discharge date	Yes	Yes	No, liaison department is overloaded	Yes	No, liaison nurse has to wait for final discharge date	Yes
Was it policy to set up patient handovers within 48h after admission?	No	No	No	No	No	No	No	No
Did physicians hold discharge conversations, using a checklist during the pilot period?	No	No	No	No	No	No	No	No
Does the nurse holds a discharge conversation, using a checklist?	Yes	No	Yes	Yes	Yes	No	Yes	Yes
Was the patient discharge letter implemented?	Yes, but only for some diagnosis at internal medicine ward.	No	No	No	No	Yes , but only at internal medicine ward for frail older patients.	No	No, a discharge summary was implemented instead.
Implementation points	*3.5*	*2*	*4*	*4*	*3*	*2.5*	*3*	*4.5*
**Education of physicians and nurses**								

For peer review only

How were physicians and nurses informed about the TIP and how often?		Kick-off meeting at participating wards; during morning report; working instructions were sent by email to all physicians		Kick-off meeting; meeting at participating wards; E-learning; 1 feedback meeting		During morning reports; project leader informed every physician separately; intranet; email		During morning reports; intranet; email; posters & pocket cards; and project leaders went to participating wards to inform physicians and nurses		Email and project leader went to participating wards		Kick-off meeting, during several morning reports		Physicians were not educated with regard to the intervention		During several morning report; email; project leaders went to participating wards to inform physicians and nurses; medical specialists from project group informed physicians in person	
Did physicians and/or nurses receive feedback with regard to their discharge letters and if yes, how often?		No		No		No		No		No		Only for nurses		No		Yes, daily on internal medicine and monthly on surgery ward, via email.	
Implementation points		*3*		*4*		*4*		*5*		*2*		*2.5*		*0*		*5*	
**Total implementation points**		**26.5**		**26**		**26**		**34**		**22**		**9**		**5**		**33.5**	
**Pre-intervention vs. post-intervention period scores**																	
**Pre-intervention period**																	
median	% letters within 24h	8.15	9.0	0.90	47.3	6.71	23.5	10.48	13.1	0.79	50.0	6.79	9.2	14.21	7.6	5.83	20.9
**Post-intervention**																	
median	% letters within 24h	9.08	19.5	1.0	48.5	5.48	24.2	5.79	19.7	0.29	49.6	7.98	16.7	22.44	1.5	0.83	53.8

For peer review only

Revised Standards for Quality Improvement Reporting Excellence (SQUIRE 2.0)
September 15, 2015

Text Section and Item Name	Section or Item Description
**Notes to authors**	• The SQUIRE guidelines provide a framework for reporting new knowledge about how to improve healthcare<br>• The SQUIRE guidelines are intended for reports that describe system level work to improve the quality, safety, and value of healthcare, and used methods to establish that observed outcomes were due to the intervention(s).<br>• A range of approaches exists for improving healthcare. SQUIRE may be adapted for reporting any of these.<br>• Authors should consider every SQUIRE item, but it may be inappropriate or unnecessary to include every SQUIRE element in a particular manuscript.<br>• The SQUIRE Glossary contains definitions of many of the key words in SQUIRE.<br>• The Explanation and Elaboration document provides specific examples of well-written SQUIRE items, and an in-depth explanation of each item.<br>• Please cite SQUIRE when it is used to write a manuscript.
**Title and Abstract**	
**1. Title**	Indicate that the manuscript concerns an initiative to improve healthcare (broadly defined to include the quality, safety, effectiveness, patient-centeredness, timeliness, cost, efficiency, and equity of healthcare)
**2. Abstract**	a. Provide adequate information to aid in searching and indexing<br>b. Summarize all key information from various sections of the text using the abstract format of the intended publication or a structured summary such as: background, local problem, methods, interventions, results, conclusions
**Introduction**	*Why did you start?*
**3. Problem Description**	Nature and significance of the local problem
**4. Available knowledge**	Summary of what is currently known about the problem, including relevant previous studies

5. Rationale	Informal or formal frameworks, models, concepts, and/or theories used to explain the problem, any reasons or assumptions that were used to develop the intervention(s), and reasons why the intervention(s) was expected to work
6. Specific aims	Purpose of the project and of this report
**Methods**	*What did you do?*
7. Context	Contextual elements considered important at the outset of introducing the intervention(s)
8. Intervention(s)	a. Description of the intervention(s) in sufficient detail that others could reproduce it<br>b. Specifics of the team involved in the work
9. Study of the Intervention(s)	a. Approach chosen for assessing the impact of the intervention(s)<br>b. Approach used to establish whether the observed outcomes were due to the intervention(s)
10. Measures	a. Measures chosen for studying processes and outcomes of the intervention(s), including rationale for choosing them, their operational definitions, and their validity and reliability<br>b. Description of the approach to the ongoing assessment of contextual elements that contributed to the success, failure, efficiency, and cost<br>c. Methods employed for assessing completeness and accuracy of data
11. Analysis	a. Qualitative and quantitative methods used to draw inferences from the data<br>b. Methods for understanding variation within the data, including the effects of time as a variable
12. Ethical Considerations	Ethical aspects of implementing and studying the intervention(s) and how they were addressed, including, but not limited to, formal ethics review and potential conflict(s) of interest
**Results**	*What did you find?*
13. Results	a. Initial steps of the intervention(s) and their evolution over time (*e.g.*, time-line diagram, flow chart, or table), including modifications made to the intervention during the project<br>b. Details of the process measures and outcome<br>c. Contextual elements that interacted with the intervention(s)<br>d. Observed associations between outcomes, interventions, and relevant contextual elements<br>e. Unintended consequences such as unexpected benefits, problems, failures, or costs associated with the intervention(s).<br>f. Details about missing data
**Discussion**	*What does it mean?*
14. Summary	a. Key findings, including relevance to the rationale and specific aims<br>b. Particular strengths of the project

For peer review only

**15. Interpretation**	a. Nature of the association between the intervention(s) and the outcomes<br>b. Comparison of results with findings from other publications<br>c. Impact of the project on people and systems<br>d. Reasons for any differences between observed and anticipated outcomes, including the influence of context<br>e. Costs and strategic trade-offs, including opportunity costs
**16. Limitations**	a. Limits to the generalizability of the work<br>b. Factors that might have limited internal validity such as confounding, bias, or imprecision in the design, methods, measurement, or analysis<br>c. Efforts made to minimize and adjust for limitations
**17. Conclusions**	a. Usefulness of the work<br>b. Sustainability<br>c. Potential for spread to other contexts<br>d. Implications for practice and for further study in the field<br>e. Suggested next steps
**Other information**	
**18. Funding**	Sources of funding that supported this work. Role, if any, of the funding organization in the design, implementation, interpretation, and reporting

For peer review only

Table 2. Glossary of key terms used in SQUIRE 2.0. This Glossary provides the intended meaning of selected words and phrases as they are used in the SQUIRE 2.0 Guidelines. They may, and often do, have different meanings in other disciplines, situations, and settings.

Assumptions
Reasons for choosing the activities and tools used to bring about changes in healthcare services at the system level.

Context
Physical and sociocultural makeup of the local environment (for example, external environmental factors, organizational dynamics, collaboration, resources, leadership, and the like), and the interpretation of these factors ("sense-making") by the healthcare delivery professionals, patients, and caregivers that can affect the effectiveness and generalizability of intervention(s).

Ethical aspects
The value of system-level initiatives relative to their potential for harm, burden, and cost to the stakeholders. Potential harms particularly associated with efforts to improve the quality, safety, and value of healthcare services include opportunity costs, invasion of privacy, and staff distress resulting from disclosure of poor performance.

Generalizability
The likelihood that the intervention(s) in a particular report would produce similar results in other settings, situations, or environments (also referred to as external validity).

Healthcare improvement
Any systematic effort intended to raise the quality, safety, and value of healthcare services, usually done at the system level. We encourage the use of this phrase rather than "quality improvement," which often refers to more narrowly defined approaches.

Inferences
The meaning of findings or data, as interpreted by the stakeholders in healthcare services – improvers, healthcare delivery professionals, and/or patients and families

Initiative
A broad term that can refer to organization-wide programs, narrowly focused projects, or the details of specific interventions (for example, planning, execution, and assessment)

Internal validity
Demonstrable, credible evidence for efficacy (meaningful impact or change) resulting from introduction of a specific intervention into a particular healthcare system.

Intervention(s)
The specific activities and tools introduced into a healthcare system with the aim of changing its performance for the better. Complete description of an intervention includes its inputs, internal activities, and outputs (in the form of a logic model, for example), and the mechanism(s) by which these components are expected to produce changes in a system's performance.

Opportunity costs

For peer review only

Loss of the ability to perform other tasks or meet other responsibilities resulting from the diversion of resources needed to introduce, test, or sustain a particular improvement initiative

Problem
Meaningful disruption, failure, inadequacy, distress, confusion or other dysfunction in a healthcare service delivery system that adversely affects patients, staff, or the system as a whole, or that prevents care from reaching its full potential

Process
The routines and other activities through which healthcare services are delivered

Rationale
Explanation of why particular intervention(s) were chosen and why it was expected to work, be sustainable, and be replicable elsewhere.

Systems
The interrelated structures, people, processes, and activities that together create healthcare services for and with individual patients and populations. For example, systems exist from the personal self-care system of a patient, to the individual provider-patient dyad system, to the microsystem, to the macrosystem, and all the way to the market/social/insurance system. These levels are nested within each other.

Theory or theories
Any "reason-giving" account that asserts causal relationships between variables (causal theory) or that makes sense of an otherwise obscure process or situation (explanatory theory). Theories come in many forms, and serve different purposes in the phases of improvement work. It is important to be explicit and well-founded about any informal and formal theory (or theories) that are used.

For peer review only
